# Isolation and Structure-Activity Relationship of Subergorgic Acid and Synthesis of Its Derivatives as Antifouling Agent

**DOI:** 10.3390/md17020101

**Published:** 2019-02-06

**Authors:** Jun Zhang, Wei Ling, Zhiqiang Yang, Yan Liang, Linyan Zhang, Can Guo, Kailing Wang, Balian Zhong, Shihai Xu, Ying Xu

**Affiliations:** 1 National Engineering Research Center of Navel Orange, Gannan Normal University, Ganzhou 341000, China; lw791010@163.com (W.L.); 15707975909@163.com (Z.Y.); zjzzf2011@gmail.com (Y.L.); 18573488362@139.com (L.Z.); 15225960215@139.com (C.G.); bal.zh@163.com (B.Z.); 2 Department of Chemistry, Jinan University, Guangzhou 510000, China; 3 College of Life Science and Oceanography, Shenzhen University, Shenzhen 518000, China; 15216101485@163.com

**Keywords:** subergorgic acid, antifouling, *Balanus amphitrite*, structure-activity relationship

## Abstract

In this study, as part of our continuous search for environmentally-friendly antifoulants from natural resources, subergorgic acid (SA) was identified from the gorgonian coral *Subergorgia suberosa*, demonstrating non-toxic, significant inhibitory effects (EC_50_ 1.25 μg/mL, LC_50_ > 25 μg/mL) against the settlement of *Balanus amphitrite*. To further explore the bioactive functional groups of SA and synthesize more potent antifouling compounds based on the lead SA, the structure-activity relationships of SA were studied, followed by rational design and synthesis of two series of SA derivatives (one being benzyl esters of SA and another being SA derivatives containing methylene chains of various lengths). Our results indicated that (1) both the double bond and ketone carbonyl are essential elements responsible for the antifouling effect of SA, while the acid group is not absolutely necessary for maintaining the antifouling effect; (2) all benzyl esters of SA displayed good antifouling effects (EC_50_ ranged from 0.30 to 2.50 μg/mL) with the most potent compound being **5** (EC_50_ 0.30 μg/mL, LC_50_ > 25 μg/mL), which was over four-fold more potent than SA; and (3) the introduction of a methylene chain into SA reduces the antifouling potency while the length of the methylene chain may differently influence the antifouling effect, depending on the functional group at the opposite site of the methylene chain. Not only has this study successfully revealed the bioactive functional groups of SA, contributing to the mechanism of SA against the settlement of *B. amphitrite*, but it has also resulted in the identification of a more potent compound **5**, which might represent a non-toxic, high-efficiency antifoulant.

## 1. Introduction

Marine biofouling, formed by the settlement of microfoulers (marine bacteria, protozoans, and microalgae) and macrofoulers (invertebrates and macroalgae) on the underwater surface of artificial substrates [1,2], have caused huge material and economic losses in the maintenance of mariculture facilities, shipping facilities, vessels, and seawater pipelines [3,4,5]. Coatings containing metal-based antifoulants, such as organotins, have been widely applied on the surface of facilities to control marine biofouling for decades. Although highly potent against targeted foulers, these kinds of metal-based materials are significantly toxic toward many non-targeted marine organisms [6,7,8,9], leading the International Maritime Organization (IMO) to prohibit their application to ships effective from 17 September 2008 [10]. Hence, there is a very urgent need to develop highly effective but environmentally-friendly antifouling alternatives.

**Figure 1:**
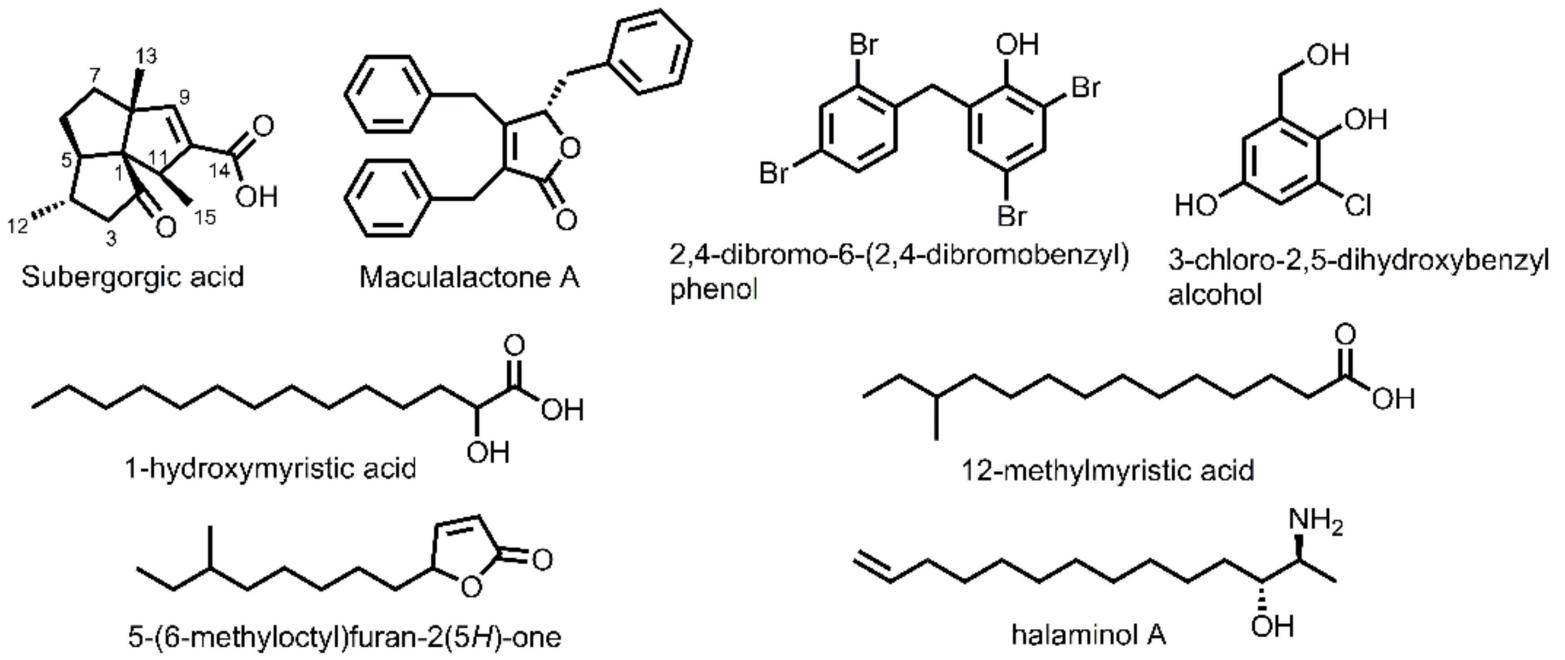
Chemical structures of subergorgic acid and some natural antifouling compounds reported in the literature.

The fact that some sessile marine invertebrates such as corals are capable of maintaining their body surface free of fouling inspires researchers to suspect that these invertebrates might adopt a chemical defense strategy by secreting secondary metabolites as potent antifouling agents. Indeed, during past years a lot of antifouling metabolites have been identified from varieties of sessile marine invertebrates [11,12,13,14,15,16,17]. As part of our continuous searching for environmentally beneficial antifouling compounds from marine resources, the gorgonian coral *S. suberosa* has been systematically investigated, resulting in the isolation of many antifouling compounds involving one novel butenolide [18] and two unusual cholestane derivatives [19]. This current study reports the isolation and structure-activity relationship of subergorgic acid (SA) from *S. suberosa* and the synthesis of its derivatives as antifouling agents. SA was first isolated by Long et al. from the gorgonian coral *S. suberosa*, whose structure has been established as an unusual tricyclic sesquiterpene (Figure 1) [20]. Interestingly, our biological test indicated SA exhibited a significantly non-toxic inhibitory effect against the settlement of *B. amphitrite* with EC_50_ being 1.25 ± 0.14 μg/mL and LC_50_ > 25 μg/mL, which is consistent with data (EC_50_ 1.2 μg/mL, LC_50_ > 200 μg/mL) reported by Qi et al. [21]. In addition to its excellent antifouling effect, there was a huge amount of SA present in the gorgonian coral *S. suberosa*, strongly prompting our research interest in SA. Thus, the structure-activity relationship of SA as a potent antifouling compound was studied to identify the bioactive functional groups, followed by the design and synthesis of SA derivatives.

A literature survey indicated that benzyl groups are often present in the antifouling compounds as a bioactive moiety. Maculalactone A (Figure 1), which contains three benzyl groups, was highly effective against the naupliar larvae of several barnacles, with EC_50_ values of 1.1–5.2 μg/mL [22]. Similarly, 2,4-dibromo-6-(2,4-dibromobenzyl)phenol [23] and 3-chloro-2,5-dihydroxybenzylalcohol [24], each containing one benzyl group, demonstrated significant settlement inhibition against barnacle larvae with EC_50_ values of 1.26 μM and 3.19–3.81 μg/mL, respectively. Meanwhile, many compounds bearing methylene chains, such as 1-hydroxymyristic acid [25], 12-methylmyristic acid [26], 5-(6-methyloctyl)furan-2(5H)-one [27], and halaminol A [28], as depicted in Figure 1, were reported to display excellent antifouling effects, indicative of the importance of methylene chains as an antifouling functional group. Therefore, based on our literature review, two series of SA derivatives, one containing benzyl functional groups and the other involving the methylene chain of various lengths, were designed and synthesized in this study, aiming to identify more potent antifouling compounds using SA as a lead.

## 2. Results and Discussion

### 2.1. Isolation of SA from the Gorgonian Coral S. suberosa

It was discovered that there was high amount of SA in *S. suberosa* (10.5 mg/g dry weight of crude extract), allowing us to hypothesize that the SA might be the primary defensive chemical produced by the gorgonian as a natural antifoulant. Meanwhile, the procedure required for the SA isolation was relatively easy with only simple extractions and one-time column chromatography (CC) purification needed. In view of the rich presence and relatively easy preparation of SA from *S. suberosa*, the potential of SA or its derivatives as novel antifoulants was attractive.

### 2.2. Structure-Activity Relationship Studies of SA

**Scheme 1:**
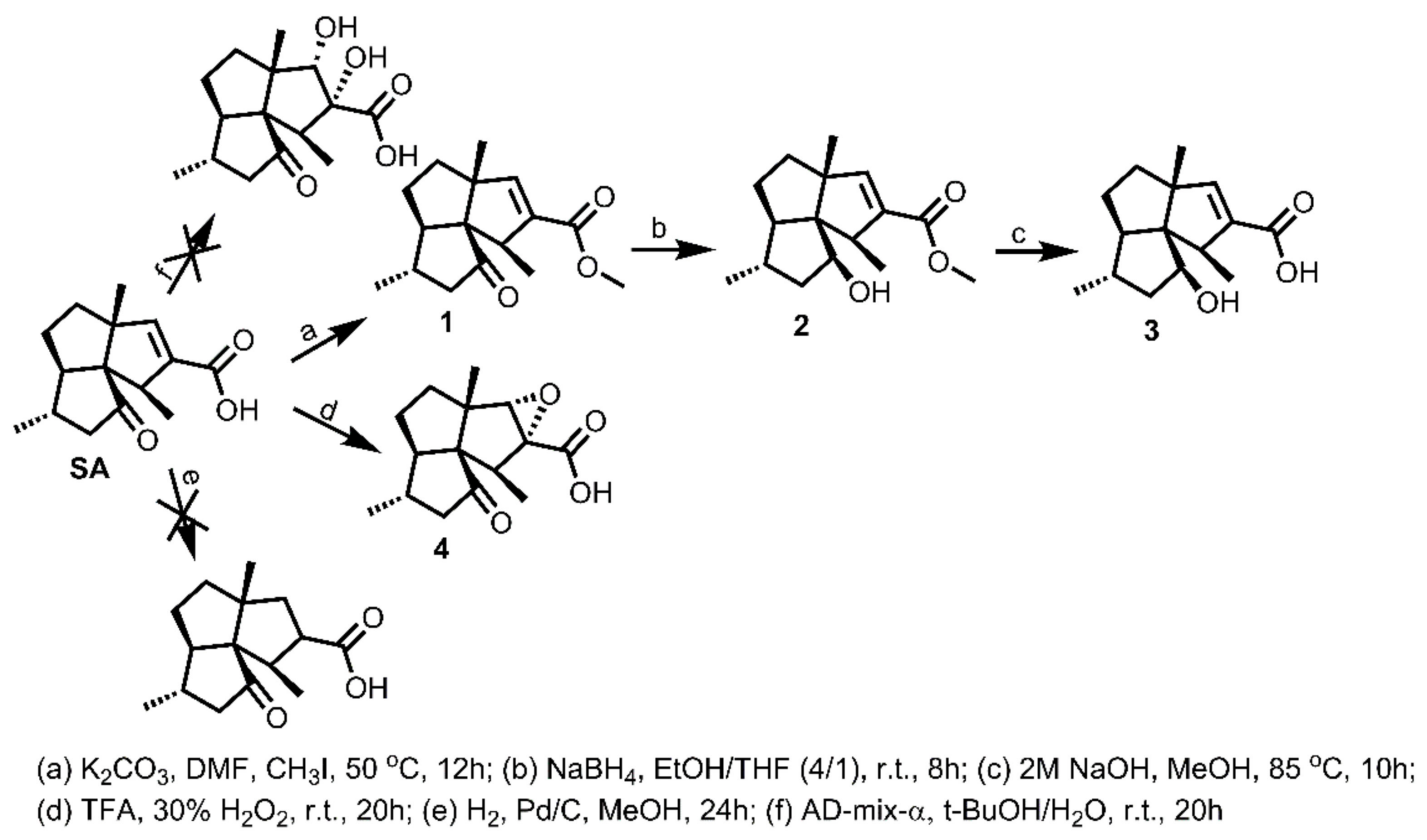
Identification of bioactive functional groups of supergorgic acid (SA).

Primarily, there are three functional groups present in SA: an acid, a ketone carbonyl, and a double bond. The modification procedures of these functional groups are depicted in Scheme 1. The esterification product **1** was readily obtained from SA using CH_3_I as a methylating reagent and K_2_CO_3_ as a base. We then planned to directly reduce the ketone carbonyl of SA to a hydroxyl group with NaBH_4_ or LiAlH_4_. Unfortunately, the reaction products were very complex, as indicated by TLC (Thin Layer Chromatography) analysis, which might be attributable to the presence of the acid group. Just as we suspected, when starting from ester **1**, the reductive reaction went very well with NaBH_4_ to give **2**. Furthermore, LiOH (3eq)/TFH/H_2_O was used, trying to remove the protecting group methyl to give **3**. However, this reaction failed to go forward at 50 °C overnight with high amounts of **2** left, making us turn to stronger reaction conditions and using 2 M NaOH at 85 °C overnight, which successfully furnished **3** in 94% yield. Interestingly, compound **3** has been isolated from the Indian ocean gorgonian coral *S. suberosa* previously [29]. However, this is the first report regarding its synthesis from SA. As for the modification of the double bond of SA, the Pd/C catalyzed hydrogenation was adopted, trying to saturate the double bond; this reaction, by contrast, failed to give the desired product with the starting material remaining unchanged. The Sharpless dihydroxylation reaction was then employed, attempting to modify the double bond into two hydroxyl groups, which also encountered failure, leaving SA unchanged. Fortunately, when using the strong oxidative reagent 30% H_2_O_2_/TFA, the double bond of SA was successfully epoxidized to furnish **4** in good yield. The relative stereochemistry of the epoxide ring of **4** was determined as shown in Scheme 1, based on the NOESY (Nuclear Overhauser Effect Spectroscopy) spectrum where H-9 was clearly correlated with the proton signals of the two methyls CH_3_-13 and CH_3_-15 (Supplementary Figures S1 and S13). With compounds **1**–**4** at hand, their antifouling effects against the settlement of B. amphitrite were tested and compared with those of SA. As shown in Table 1, ester **1** maintained most of the antifouling activity of SA, with an EC_50_ of 3.16 ± 0.18 μg/mL, which was slightly less than that of SA (EC_50_ 1.25 ± 0.14 μg/mL), indicating that the acid group is not absolutely necessary for maintaining the antifouling effect of SA. Meanwhile, similar to SA, compound **1** exhibited non-toxic effects toward tested larva with LC_50_ > 25 μg/mL. Surprisingly, after either the ketone carbonyl or the double bond group was modified, none of the obtained compounds **3** or **4** still exhibited antifouling activity at concentrations up to 25 μg/mL, clearly demonstrating that both the ketone carbonyl and the double bond groups are essential elements responsible for the antifouling effect. Based on these preliminary structure-activity relationship studies, the key pharmacophores of SA as a potent antifouling compound were experimentally determined, providing a basis for the rational design of more potent SA derivatives.

### 2.3. Synthesis and Antifouling Activities of Benzyl Esters of SA

**Scheme 2:**
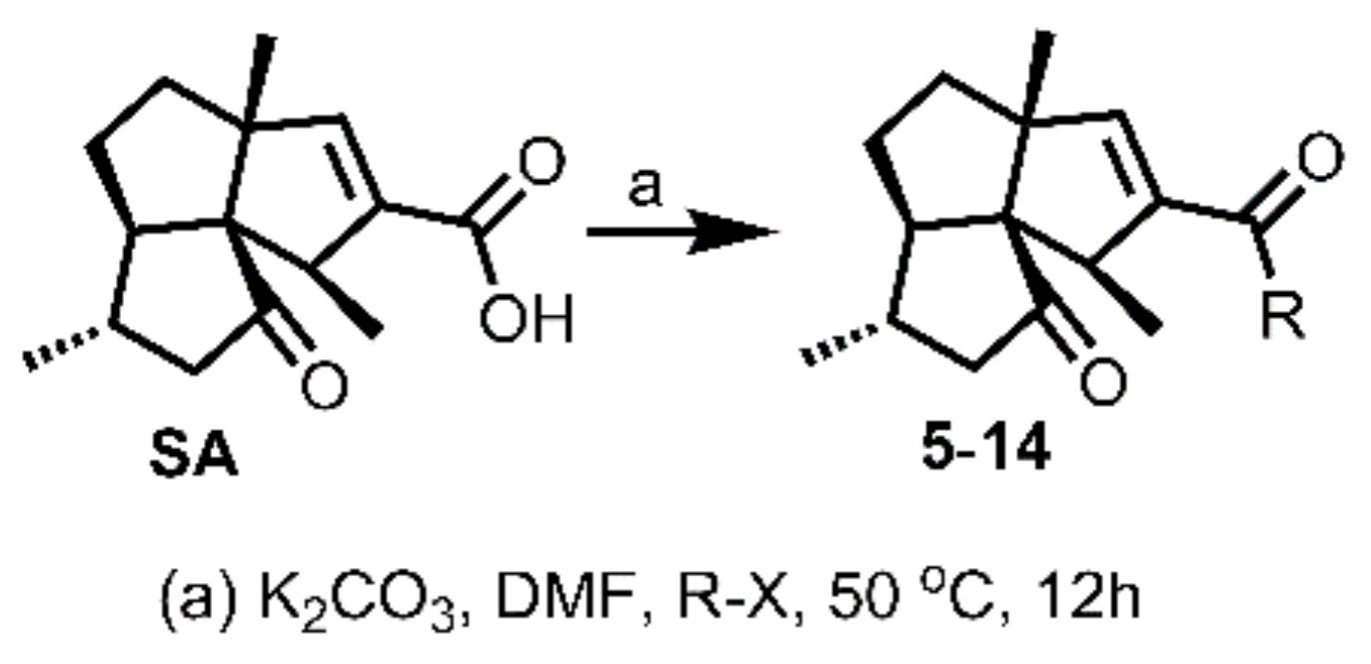
Synthesis of SA derivatives **5**–**14**.

The preliminary structure-activity relationship studies revealed that both the ketone carbonyl and double bond were essential groups responsible for antifouling effects with the methyl esterification of the acid group having negligible influence on the antifouling activity, focusing the following design and synthesis of SA derivatives on the modification of the acid group. According to the methods depicted in Scheme 2, using dry DMF as a solvent and K_2_CO_3_ as a base, SA could react smoothly with varieties of benzyl halides to give desired benzyl esters with almost quantitive yields. Interestingly, as listed in Table 2, all benzyl esters exhibited good antifouling effects against the settlement of B. amphitrite with EC_50_ ranging from 0.30 to 2.50 μg/mL. The most effective compound was **5** (EC_50_ 0.30 ± 0.10 μg/mL), which contained a non-substituted benzyl group, indicative of the substituents around the benzene ring reducing the antifouling effect. Another interesting observation was that the substituted position on the benzene ring might influence the antifouling effect with the o or m substitution being less influential than p substitution. This observation was tentatively supported by the EC_50_ values listed in Table 2, in which compounds **6** (EC_50_ 1.20 ± 0.17 μg/mL), **7** (EC_50_ 1.26 ± 0.11 μg/mL), and **12** (EC_50_ 2.50 ± 0.18 μg/mL), all having p substitution, demonstrated much lower antifouling effects compared to the o or m substituted compounds **8** (EC_50_ 0.40 ± 0.12 μg/mL), **10** (EC_50_ 0.65 ± 0.13 μg/mL), and **11** (EC_50_ 0.61 ± 0.13 μg/mL). Furthermore, it looked like the type of substituents around the benzene ring might exhibit limited influence on antifouling activity, evidenced from the biological effects of pairs between **6** with **7** and between **10** with **11**, with each pair containing different substituents while displaying almost the same antifouling potency. Notably, compound **9** (EC_50_ 1.28 ± 0.17 μg/mL), however, while having an o substituent on the benzene ring, displayed much lower potency compared to other o or m substituted compounds **8**, **10**, and **11**, which might be due to steric effects induced by the bulky benzene ring. Therefore, based on these experimental results, we tentatively assumed that steric effects might highly influence the antifouling activity of benzyl esters of SA. Compound **5**, which bears a non-substituted benzene ring, occupies the least space and has the least steric effects and thus shows the best antifouling effect. To further support this assumption, compounds **13** and **14**, which are very close to **5** in molecular size, were synthesized by replacement of starting material benzyl bromide with 2-(bromomethyl)pyridine and 4-(bromomethyl)pyridine, respectively. In good agreement with our assumption, these two pyridine-containing SA derivatives demonstrated excellent antifouling effects, with EC_50_ being 0.32 ± 0.11 and 0.63 ± 0.12 μg/mL for **13** and **14**, respectively. Worthy of note, as shown in Table 2, is that compounds **5**–**14** were non-toxic toward the larva B. amphitrite at concentrations up to 25 μg/mL, strongly supporting the potency of SA derivatives as environmentally-friendly antifoulants.

### 2.4. Synthesis and Antifouling Activities of SA Derivatives Containing Methylene Chain of Various Lengths

**Scheme 3:**
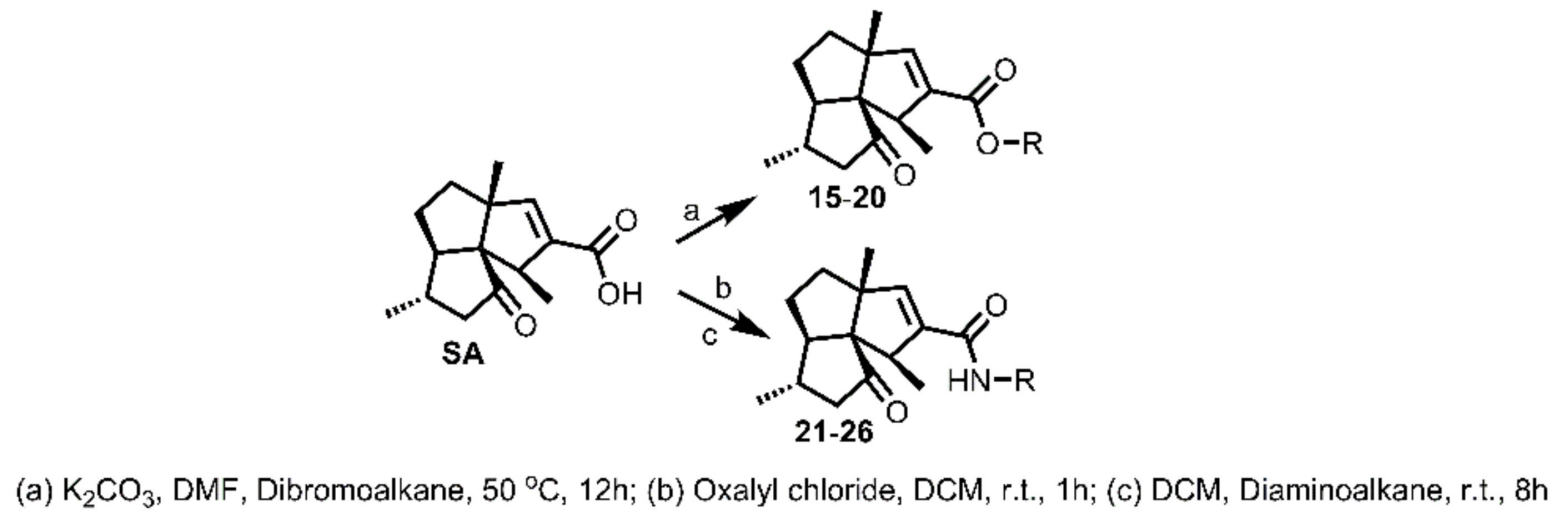
Synthesis of SA derivatives **15**–**26**.

Methylene chains have been found within many antifouling compounds as a bioactive moiety [25,26,27,28]. Additionally, the functional groups -Br and -NH_2_ have been found to be present in some potent antifoulants such as 2,4-dibromo-6-(2,4-dibromobenzyl)phenol [23] and halaminol A [28] (Figure 1). Hence, a spectrum of SA derivatives containing methylene chains of various lengths and -Br or -NH_2_ groups were designed and synthesized to identify their influence on the antifouling activity of SA derivatives. As shown in Scheme 3, using SA and dibromoalkanes of various lengths as starting materials, compounds **15**–**20** were easily synthesized in good yields. In order to avoid the formation of dimers of SA, over four equivalents of dibromoalkane was used for each reaction. Compounds **15**–**20** were then subjected to antifouling screening. Unfortunately, as revealed in Table 3, none of these compounds exhibited better or comparable effects when compared with SA, with the activity following an order of **15** (EC_50_ 2.05 ± 0.16 μg/mL) > **16** (EC_50_ 2.48 ± 0.11 μg/mL) > **17** (EC_50_ 2.75 ± 0.13 μg/mL) > **18** (EC_50_ 7.51 ± 0.22 μg/mL) > **19** (EC_50_ >25 μg/mL) > **20** (EC_50_ >25 μg/mL). This order was correlated with the lengths of the methylene chains, with greater length resulting in less potency. Furthermore, another kind of methylene chain-containing compounds **21**–**26**, which differ from **15**–**20** mainly in the replacement of the -Br with the -NH_2_ group, were designed and synthesized according to the procedure briefly depicted in Scheme 3. Firstly, SA was reacted with oxalyl chloride to give SA acid chloride, which was then directly reacted with series of diaminoalkanes of various lengths to give SA amides **21**–**26**. The antifouling effects of **21**–**26** were then screened, with results being reported in Table 3. Interestingly, all compounds **21**–**26**, in addition to being less potent than SA, displayed antifouling effects in the order **26** (EC_50_ 4.50 ± 0.10 μg/mL) > **25** (EC_50_ 20.00 ± 0.15 μg/mL) > **21**–**24** (EC_50_ > 25 μg/mL), which is exactly opposite to the trend of compounds **15**–**20**, with **26**, which contains the longest methylene chain, exhibiting the best antifouling effect. The length of the methylene chain differently influenced the antifouling effect of these two kinds of SA derivatives, the assumed reason for which might be that -NH_2_ was more potent than -Br in interacting with the bonding protein of larva, including the formation of hydrogen bonds. Therefore, based on these experimental results, it might be deduced that the introduction of a methylene chain into SA would result in a reduction in activity, while the length of the methylene chain might differently influence the antifouling effect depending on the functional group at the opposite site of the methylene chain.

## 3. Materials and Methods

### 3.1. General

All reactions were carried out using commercially available starting materials or reagents unless otherwise stated. Optical rotations were measured on a WZZ-3A automatic polarimeter (Shanghai YiCe Apparatus & Equipment co. LTD, Shanghai, China), NMR spectra were recorded on a Varian Mercury VX 300 spectrometer (Varian, Tokyo, Japan) or Bruker 400 MHz spectrometer (Bruker, Berlin, Germany) using TMS as an internal standard, and the chemical shifts were reported in ppm relative to CDCl_3_ (δ_H_ 7.26 and δ_C_ 77.16). High resolution mass spectra (HRMS) were performed on a QSTAR XL mass spectrometry system (Applied Biosystem Co., Foster City, CA, USA) or on a TripleTOF 6600 mass spectrometer (ABSciex, Redwood City, CA, USA). Column chromatography (CC) was performed with silica gel (230–400 mesh, Merck, Darmstadt, Germany). TLC was carried out with an aluminum sheet precoated silica gel G_60_ (Merck, Darmstadt, Germany). Spots were visualized under UV light or by spraying with 5% H_2_SO_4_ in EtOH followed by heating.

### 3.2. Isolation of SA

#### 3.2.1. Animal Material

Gorgonian coral S. suberosa was collected at Naozhou Island, in Zhanjiang, South China Sea, China, in May 2011, and was identified by Dr. Rob van Soest (University of Amsterdam, The Netherlands). A voucher specimen (2011-05) has been deposited in the Laboratory of Natural Products of Department of Chemistry, Jinan University, Guangzhou, China.

#### 3.2.2. Extraction and Isolation

The gorgonian corals *S. suberosa* (6.5 kg, wet weight) were extracted with 95% ethanol (EtOH, 20 L × 3) three times (each time for 30 days) at room temperature. The EtOH extracts were concentrated under vacuum to give a brown gum (200 g, dried crude extract), which was partitioned between ethyl acetate (EtOAc, 1.5 L × 3) and H_2_O (1.5 L). The EtOAc fraction (140 g) obtained was then subjected to CC on silica gel (700 g; 9.0 cm diam × 100.0 cm height), eluted with a step gradient of EtOAc in petroleum ether (PE) (10–100%), and then with MeOH-EtOAc (10–30%), to give 11 fractions (Frs. A-K) and yield the pure compound SA (2.1 g, 1.05% isolated yield from the dried crude extract, mainly from the 30% EtOAc/PE gradient). SA was obtained as white powder whose chemical structure was determined by spectroscopic methods (^1^H NMR, ^13^C NMR, ^1^H-^1^H COSY, HSQC, and HMBC). ^1^H NMR (400 MHz, CDCl_3_) δ_H_: 6.43 (s, 1H, H-9), 3.00 (q, *J* = 7.2 Hz, 1H, H-11), 2.36 (dd, *J* = 16.8, 6.8 Hz, 1H, H_a_-3), 2.08 (m, 1H, H-5), 2.00 (dd, *J* = 16.8, 12.8 Hz, 1H, H_b_-3), 1.79 (m, 1H, H_a_-7), 1.56–1.67 (m, 4H, H-4, H-6, H_b_-7), 1.21 (s, 3H, H-13), 1.12 (d, *J* = 7.2 Hz, 3H, H-15), 1.11 (d, *J* = 6.4 Hz, 3H, H-12); ^13^C NMR (100 MHz, CDCl_3_) δ_C_: 217.9 (C-2), 169.5 (C-14), 152.5 (C-9), 136.7 (C-10), 68.7 (C-1), 62.8 (C-5), 61.9 (C-8), 51.8 (C-11), 50.0 (C-3), 38.4 (C-7), 33.5 (C-4), 28.5 (C-6), 23.6 (C-13), 20.0 (C-12), 17.9 (C-15). The NMR data of SA was in good agreement with that reported in the literature [21].

### 3.3. Synthesis Experiments

#### 3.3.1. Synthesis of **1**–**4**

To a solution of SA (50 mg, 0.20 mmol) in DMF (2 mL, freshly distilled from CaH_2_), was added K_2_CO_3_ (80 mg, 0.58 mmol), followed by the addition of iodomethane (26 μL, 0.41 mmol). The obtained mixture was then stirred under N_2_ atmosphere at 50 °C for 12 h. Afterwards, the reaction mixture was cooled to room temperature and quenched by brine (10 mL). The mixture was then extracted with DCM (8 mL × 3), washed successively with brine (10 mL × 3) and water (10 mL × 3), dried over Na_2_SO_4_, filtered, and concentrated in vacuo. The residue was purified by silica gel CC (EtOAc/PE = 20:80) to yield **1** (49.8 mg, 95% yield) as an off-yellow oil. ${\left[\alpha \right]}_{D}^{23}$ − 30.5 (c 0.09, DCM); ^1^H NMR (300 MHz, CDCl_3_) δ_H_: 6.27 (s, 1H), 3.72 (s, 3H), 3.00 (q, *J* = 6.9 Hz, 1H), 2.34 (dd, *J* = 16.8, 6.9 Hz, 1H), 2.08 (m, 1H), 1.98 (dd, *J* = 16.8, 12.6 Hz, 1H), 1.75–1.79 (m, 1H), 1.54–1.67 (m, 4H), 1.19 (s, 3H), 1.10 (d, *J* = 6.9 Hz, 6H); ^13^C NMR (75 MHz, CDCl_3_) δ_C_: 217.9, 165.0, 149.7, 137.1, 68.6, 62.8, 61.8, 52.1, 51.6, 50.1, 38.4, 33.5, 28.4, 23.7, 20.0, 18.0; HRMS *m*/*z* 263.1650 [M+H]^+^ (calcd. for C_16_H_23_O_3_ at *m*/*z* 263.1647).

To a solution of **1** (30 mg, 0.11 mmol) in EtOH/TFH (*v*/*v* = 4/1, 5 mL), was added NaBH_4_ (10 mg, 0.26 mmol). The obtained mixture was then stirred under N_2_ atmosphere at room temperature for 8 h. Afterwards, the reaction mixture was quenched by addition of acetone (2 mL), followed by stirring for half an hour. The mixture was then evaporated to remove all solvent. Water (5 mL) was added into the residue to form a suspension, which was then extracted with DCM (5 mL × 3). The combined DCM extract was washed successively with brine (10 mL × 3) and water (10 mL × 3), dried over Na_2_SO_4_, filtered, and concentrated in vacuo. The residue was purified by silica gel CC (EtOAc/PE = 10:90) to yield **2** (24.6 mg, 86% yield) as an off-white powder. ${\left[\alpha \right]}_{D}^{23}$ + 93.1 (c 0.41, DCM); ^1^H NMR (300 MHz, CDCl_3_) δ_H_: 6.29 (s, 1H), 4.15 (m, 1H), 3.73 (s, 3H), 3.09 (q, *J* = 6.9 Hz, 1H), 1.92 (m, 1H), 1.84–1.87 (m, 1H), 1.61–1.65 (m, 2H), 1.47–1.54 (m, 4H), 1.54–1.67 (m, 4H), 1.35 (d, *J* = 6.9 Hz, 3H), 1.20 (s, 3H), 1.13 (d, *J* = 6.9 Hz, 3H); ^13^C NMR (75 MHz, CDCl_3_) δ_C_: 165.4, 150.1, 139.2, 77.8, 66.4, 62.6, 57.8, 51.5, 49.7, 43.4, 40.0, 37.8, 31.6, 23.0, 20.1, 19.9; HRMS *m*/*z* 265.1799 [M + H]^+^ (calcd. for C_16_H_25_O_3_ at *m*/*z* 265.1804), 247.1694 [M − H_2_O + H]^+^ (calcd. for C_16_H_23_O_2_ at *m*/*z* 247.1698).

Compound **2** (20 mg, 0.076 mmol) was added into the flask containing NaOH aqueous solution (2 mol/L, 2 mL), followed by addition of methanol (0.2 mL) to form a clear solution. A condenser was connected to the flask. The solution was then stirred under N_2_ atmosphere at 85 °C for 10 h. Afterwards, the reaction mixture was cooled to room temperature and quenched by dropwise addition of HCl aqueous solution (1 mol/L, 5 mL). The mixture was then extracted with DCM (6 mL × 3), washed successively with brine (10 mL × 3) and water (10 mL × 3), dried over Na_2_SO_4_, filtered, and concentrated in vacuo. The residue was purified by silica gel CC (MeOH/DCM = 4:96) to yield **3** (17.9 mg, 94% yield) as a white amorphous powder. ${\left[\alpha \right]}_{D}^{23}$ + 110.9 (c 0.39, DCM); ^1^H NMR (400 MHz, CDCl_3_) δ_H_: 6.45 (s, 1H), 4.17 (m, 1H), 3.09 (q, *J* = 7.2 Hz, 1H), 1.92 (m, 1H), 1.85–1.87 (m, 1H), 1.65 (m, 1H), 1.48–1.52 (m, 4H), 1.37 (d, *J* = 6.8 Hz, 3H), 1.28 (m, 1H), 1.22 (s, 3H), 1.13 (d, *J* = 6.4 Hz, 3H); ^13^C NMR (100 MHz, CDCl_3_) δ_C_: 169.7, 152.8, 138.8, 77.8, 66.5, 62.6, 58.0, 49.4, 43.7, 40.0, 37.8, 31.6, 22.9, 20.9, 19.8; HRMS *m*/*z* 249.1499 [M − H]^+^ (calcd. for C_15_H_21_O_3_ at *m*/*z* 249.1491).

To a solution of SA (15 mg, 0.06 mmol) in TFA (trifluoroacetic acid, 1 mL), was added dropwise the 30% H_2_O_2_ (1.5 mL) at 0 °C. The obtained mixture was then stirred under N_2_ atmosphere from 0 °C to room temperature for 20 h. Afterwards, the reaction mixture was quenched by Na_2_SO_3_ aqueous solution (4 mL) at 0 °C and diluted with H_2_O (3 mL) to a pH around 1.0. The mixture was then extracted with DCM (8 mL × 3), washed successively with brine (15 mL × 3) and water (15 mL × 3), dried over Na_2_SO_4_, filtered, and concentrated in vacuo. The residue was purified by silica gel CC (MeOH/DCM = 10:90) to yield **4** (13.5 mg, 85% yield) as a white amorphous powder. ${\left[\alpha \right]}_{D}^{23}$ + 46.7 (c 0.28, DCM); ^1^H NMR (400 MHz, CDCl_3_) δ_H_: 3.56 (s, 1H), 2.74 (q, *J* = 6.9 Hz, 1H), 2.39 (dd, *J* = 16.8, 6.4 Hz, 1H), 2.00 (m, 3H), 1.78–1.86 (m, 4H), 1.11 (s, 3H), 1.06 (d, *J* = 6.8 Hz, 3H), 1.05 (d, *J* = 6.8 Hz, 3H); ^13^C NMR (75 MHz, CDCl_3_) δ_C_: 216.5, 170.9, 70.8, 70.8, 67.9, 61.1, 55.9, 49.8, 44.2, 35.7, 32.2, 27.6, 22.6, 19.3, 10.9; HRMS *m*/*z* 246.1260 [M − H_2_O]^−^ (calcd. for C_15_H_18_O_3_ at m/z 246.1256), 263.1246 [M − H]^−^ (calcd. for C_15_H_19_O_4_ at *m*/*z* 263.1283).

#### 3.3.2. General Procedure for Synthesis of **5**–**14**

To a solution of SA (15 mg, 0.06 mmol) in DMF (1 mL, freshly distilled from CaH_2_), was added K_2_CO_3_ (25 mg, 0.18 mmol), followed by the addition of benzyl halide derivative (0.12 mmol) for **5**–**12**, or 2-(bromomethyl)pyridine, 4-(bromomethyl)pyridine, for **13**–**14**, respectively. The obtained mixture was then stirred under N_2_ atmosphere at 50 °C for 12 h. Afterwards, the reaction mixture was cooled to room temperature and quenched by brine (5 mL). The mixture was then extracted with DCM (5 mL × 3), washed successively with brine (10 mL × 3) and water (10 mL × 3), dried over Na_2_SO_4_, filtered, and concentrated in vacuo. The residue was purified by silica gel CC (EtOAc/PE ranged from 10:90 to 30:70 depending on the TLC analysis) to yield **5**–**14**.

Compound **5** (19.5 mg), a colorless oil, was purified by silica gel CC (EtOAc/PE 10:90) from the reaction residue with 96% yield. ${\left[\alpha \right]}_{D}^{23}$ − 93.1 (c 0.18, DCM); ^1^H NMR (400 MHz, CDCl_3_) δ_H_: 7.33–7.39 (m, 5H), 6.33 (s, 1H), 5.19 (d, *J* = 12.4 Hz, 1H ), 5.14 (d, *J* = 12.4 Hz, 1H ), 3.03 (q, *J* = 6.8 Hz, 1H), 2.34 (dd, *J* = 16.8, 6.8 Hz, 1H), 2.07 (m, 1H), 1.98 (dd, *J* = 16.8, 12.8 Hz, 1H), 1.78 (m, 1H), 1.62–1.67 (m, 2H), 1.54–1.57 (m, 2H), 1.20 (s, 3H), 1.12 (d, *J* = 7.2 Hz, 3H), 1.10 (d, *J* = 6.4 Hz, 3H); ^13^C NMR (100 MHz, CDCl_3_) δ_C_: 217.9, 164.4, 150.1, 137.1, 136.1, 128.7, 128.7, 128.4, 128.3, 128.3, 68.6, 66.3, 62.8, 61.8, 52.0, 50.1, 38.4, 33.5, 28.4, 23.7, 20.0, 18.0; HRMS *m*/*z* 337.1811 [M − H]^−^ (calcd. for C_22_H_25_O_3_ at *m*/*z* 337.1804).

Compound **6** (23.5 mg), an off-white powder, was purified by silica gel CC (EtOAc/PE 10:90) from the reaction residue with 98% yield. ${\left[\alpha \right]}_{D}^{23}$ − 95.0 (c 0.89, DCM); ^1^H NMR (300 MHz, CDCl_3_) δ_H_: 7.40 (d, J = 8.1 Hz, 2H), 7.31 (d, J = 8.1 Hz, 2H), 6.32 (s, 1H), 5.16 (d, J = 12.3 Hz, 1H), 5.12 (d, J = 12.3 Hz, 1H), 3.04 (q, *J* = 7.2 Hz, 1H), 2.34 (dd, *J* = 16.8, 6.6 Hz, 1H), 1.94–2.07 (m, 2H), 1.75–1.78 (m, 1H), 1.53–1.66 (m, 4H), 1.32 (s, 9H), 1.19 (s, 3H), 1.12 (d, *J* = 6.6 Hz, 3H), 1.10 (d, *J* = 5.7 Hz, 3H); ^13^C NMR (75 MHz, CDCl_3_) δ_C_: 217.9, 164.5, 151.3, 149.9, 137.1, 133.1, 128.2, 128.2, 125.6, 125.6, 68.6, 66.1, 62.8, 61.8, 52.0, 50.1, 38.4, 34.7, 33.5, 31.5, 31.5, 31.5, 28.4, 23.7, 20.0, 18.0; HRMS *m*/*z* 395.2586 [M + H]^+^ (calcd. for C_26_H_35_O_3_ at *m*/*z* 395.2586).

Compound **7** (24.0 mg), a colorless oil, was purified by silica gel CC (EtOAc/PE 10:90) from the reaction residue with 96% yield. ${\left[\alpha \right]}_{D}^{23}$ − 76.1 (c 0.91, DCM); ^1^H NMR (300 MHz, CDCl_3_) δ_H_: 7.50 (d, *J* = 8.1 Hz, 2H), 7.25 (d, *J* = 8.1 Hz, 2H), 6.32 (s, 1H), 5.14 (d, *J* = 12.6 Hz, 1H), 5.08 (d, *J* = 12.6 Hz, 1H), 3.03 (q, *J* = 7.2 Hz, 1H), 2.35 (dd, *J* = 16.8, 6.6 Hz, 1H), 2.07 (m, 1H), 1.98 (dd, *J* = 16.5, 12.6 Hz, 1H), 1.77 (m, 1H), 1.54–1.68 (m, 4H), 1.20 (s, 3H), 1.11 (d, *J* = 6.3 Hz, 6H ); ^13^C NMR (75 MHz, CDCl_3_) δ_C_: 217.8, 164.2, 150.3, 136.9, 135.1, 131.8, 131.8, 130.0, 130.0, 122.4, 68.6, 65.4, 62.8, 61.8, 52.0, 50.0, 38.4, 33.5, 28.4, 23.7, 20.0, 18.0; HRMS *m*/*z* 417.1069 [M + H]^+^ (calcd. for C_22_H_26_BrO_3_ at *m*/*z* 417.1065).

Compound **8** (23.5 mg), a colorless oil, was purified by silica gel CC (EtOAc/PE 10:90) from the reaction residue with 97% yield. ${\left[\alpha \right]}_{D}^{23}$ − 68.6 (c 0.08, DCM); ^1^H NMR (300 MHz, CDCl_3_) δ_H_: 7.34 (d, *J* = 7.5 Hz, 2H), 7.23 (m, 1H), 6.29 (s, 1H), 5.43 (s, 2H), 2.99 (q, *J* = 6.9 Hz, 1H), 2.33 (dd, *J* = 16.5, 6.6 Hz, 1H), 2.06 (m, 1H), 1.97 (dd, *J* = 16.5, 12.6 Hz, 1H), 1.72–1.78 (m, 1H), 1.51–1.66 (m, 4H), 1.17 (s, 3H), 1.09 (d, *J* = 7.2 Hz, 3H), 1.09 (d, *J* = 7.2 Hz, 3H); ^13^C NMR (75 MHz, CDCl_3_) δ_C_: 217.9, 164.3, 150.3, 137.1, 136.7, 131.6, 130.6, 130.6, 128.6, 128.6, 68.6, 65.4, 62.7, 61.7, 61.2, 52.0, 50.1, 38.4, 33.5, 28.4, 23.7, 20.0, 17.9; HRMS m/z 370.1357 [M − HCl]^−^, 405.1028 [M − H]^−^ (calcd. for C_22_H_23_ClO_3_ and C_22_H_23_Cl_2_O_3_ at *m*/*z* 370.1336 and 405.1025, respectively).

Compound **9** (22.4 mg), an off-yellow oil, was purified by silica gel CC (EtOAc/PE 10:90) from the reaction residue with 90% yield. ${\left[\alpha \right]}_{D}^{23}$ − 91.4 (c 0.17, DCM); ^1^H NMR (300 MHz, CDCl_3_) δ_H_: 7.49–7.52 (m, 1H), 7.30–7.43 (m, 8H), 6.22 (s, 1H), 5.12 (s, 2H), 2.98 (q, *J* = 7.2 Hz, 1H), 2.34 (dd, *J* = 16.5, 6.6 Hz, 1H), 2.03–2.08 (m, 1H), 1.98 (dd, *J* = 16.5, 12.6 Hz, 1H), 1.72–1.76 (m, 1H), 1.54–1.66 (m, 4H), 1.19 (s, 3H), 1.11 (d, *J* = 6.6 Hz, 3H ), 1.08 (d, *J* = 7.2 Hz, 3H ); ^13^C NMR (75 MHz, CDCl_3_) δ_C_: 217.9, 164.2, 149.8, 142.4, 140.6, 137.0, 133.4, 130.4, 129.6, 129.2, 129.2, 128.4, 128.3, 128.3, 127.7, 127.5, 68.5, 64.5, 62.8, 61.8, 52.0, 50.1, 38.4, 33.5, 28.4, 23.7, 20.0, 18.0; HRMS *m*/*z* 415.2269 [M + H]^+^ (calcd. for C_28_H_31_O_3_ at *m*/*z* 415.2273).

Compound **10** (21.7 mg), an off-yellow oil, was purified by silica gel CC (EtOAc/PE 15:85) from the reaction residue with 97% yield. ${\left[\alpha \right]}_{D}^{23}$ − 126.2 (c 0.11, DCM); ^1^H NMR (300 MHz, CDCl_3_) δ_H_: 7.36 (s, 1H), 7.30–7.32 (m, 2H), 7.23–7.26 (m, 1H), 6.34 (s, 1H), 5.16 (d, J = 12.6 Hz, 1H), 5.10 (d, J = 12.6 Hz, 1H), 3.04 (q, *J* = 7.2 Hz, 1H), 2.36 (dd, *J* = 16.5, 6.6 Hz, 1H), 2.06–2.11 (m, 1H), 2.00 (dd, *J* = 16.5, 12.6 Hz, 1H), 1.78–1.81 (m, 1H), 1.55–1.65 (m, 4H), 1.21 (s, 3H), 1.12 (d, *J* = 6.6 Hz, 3H ), 1.10 (d, *J* = 6.3 Hz, 3H ); ^13^C NMR (75 MHz, CDCl_3_) δ_C_: 217.8, 164.2, 150.4, 138.1, 136.8, 134.5, 130.0, 128.5, 128.3, 126.3, 68.6, 65.4, 62.8, 61.8, 52.0, 50.1, 38.4, 33.5, 28.4, 23.7, 20.0, 18.0; HRMS *m*/*z* 371.1425 [M − H]^−^ (calcd. for C_22_H_24_ClO_3_ at *m*/*z* 371.1414).

Compound **11** (20.9 mg), a colorless oil, was purified by silica gel CC (EtOAc/PE 15:85) from the reaction residue with 95% yield. ${\left[\alpha \right]}_{D}^{23}$ − 83.0 (c 0.09, DCM); ^1^H NMR (300 MHz, CDCl_3_) δ_H_: 7.29 (m, 1H), 6.95 (d, *J* = 7.5 Hz, 1H), 6.85–6.90 (m, 2H), 6.33 (s, 1H), 5.17 (d, *J* = 12.6 Hz, 1H), 5.11 (d, *J* = 12.6 Hz, 1H), 3.81 (s, 3H), 3.04 (q, *J* = 7.2 Hz, 1H), 2.34 (dd, *J* = 16.5, 6.9 Hz, 1H), 2.05–2.10 (m, 1H), 2.00 (dd, *J* = 16.5, 12.6 Hz, 1H), 1.76–1.78 (m, 1H), 1.53–1.69 (m, 4H), 1.20 (s, 3H), 1.12 (d, *J* = 6.9 Hz, 3H ), 1.10 (d, *J* = 6.3 Hz, 3H ); ^13^C NMR (75 MHz, CDCl_3_) δ_C_: 217.9, 164.3, 159.8, 150.1, 137.6, 137.1, 129.8, 120.4, 113.8, 113.7, 68.6, 66.1, 62.8, 61.8, 55.4, 52.0, 50.1, 38.4, 33.5, 28.4, 23.7, 20.0, 18.0; HRMS *m*/*z* 367.1913 [M − H]^−^ (calcd. for C_23_H_27_O_4_ at *m*/*z* 367.1910).

Compound **12** (21.1 mg), an off-yellow oil, was purified by silica gel CC (EtOAc/PE 20:80) from the reaction residue with 96% yield. ${\left[\alpha \right]}_{D}^{23}$ − 68.3 (c 0.16, DCM); ^1^H NMR (300 MHz, CDCl_3_) δ_H_: 7.31 (d, *J* = 8.4 Hz, 2H), 6.89 (d, *J* = 8.4 Hz, 2H), 6.29 (s, 1H), 5.13 (d, *J* = 12.0 Hz, 1H), 5.07 (d, *J* = 12.0 Hz, 1H), 3.81 (s, 3H), 3.01 (q, *J* = 6.9 Hz, 1H), 2.34 (dd, *J* = 16.5, 6.9 Hz, 1H), 2.04–2.09 (m, 1H), 1.98 (dd, *J* = 16.5, 12.6 Hz, 1H), 1.74–1.78 (m, 1H), 1.56–1.66 (m, 4H), 1.18 (s, 3H), 1.10 (d, *J* = 7.2 Hz, 6H); ^13^C NMR (75 MHz, CDCl_3_) δ_C_: 217.9, 164.5, 159.7, 149.9, 137.1, 130.2, 130.2, 128.2, 114.0, 114.0, 68.6, 66.1, 62.8, 61.8, 55.4, 52.0, 50.1, 38.4, 33.5, 28.4, 23.7, 20.0, 18.0; HRMS *m*/*z* 386.2336 [M + NH_4_]^+^ (calcd. for C_23_H_32_NO_4_ at *m*/*z* 386.2331).

Compound **13** (23.8 mg), a colorless oil, was purified by silica gel CC (EtOAc/PE 25:75) from the reaction residue with 95% yield. ${\left[\alpha \right]}_{D}^{23}$ − 96.0 (c 0.17, DCM); ^1^H NMR (300 MHz, CDCl_3_) δ_H_: 8.59–8.61 (m, 1H), 7.69–7.75 (m, 1H), 7.36 (d, *J* = 7.8 Hz, 1H), 7.22–7.26 (m, 1H), 6.39 (s, 1H), 5.29 (s, 2H), 3.07 (q, *J* = 6.8 Hz, 1H), 2.36 (dd, *J* = 16.8, 6.8 Hz, 1H), 2.07–2.11 (m, 1H), 2.00 (dd, *J* = 16.8, 12.8 Hz, 1H), 1.78–1.83 (m, 1H), 1.55–1.70 (m, 4H), 1.20 (s, 3H), 1.14 (d, *J* = 7.5 Hz, 3H), 1.12 (d, *J* = 6.9 Hz, 3H); ^13^C NMR (75 MHz, CDCl_3_) δ_C_: 217.8, 164.1, 156.0, 150.4, 149.6, 136.9, 136.8, 123.0, 121.9, 68.6, 66.9, 62.8, 61.9, 52.0, 50.1, 38.4, 33.5, 28.4, 23.7, 20.0, 18.0; HRMS *m*/*z* 338.1754 [M − H]^−^ (calcd. for C_21_H_24_NO_3_ at *m*/*z* 338.1756).

Compound **14** (24.0 mg), an off-yellow oil, was purified by silica gel CC (EtOAc/PE 30:70) from the reaction residue with 96% yield. ${\left[\alpha \right]}_{D}^{23}$ − 106.8 (c 0.09, DCM); ^1^H NMR (300 MHz, CDCl_3_) δ_H_: 8.52 (d, *J* = 5.7 Hz, 2H), 7.26 (d, *J* = 5.4 Hz, 2H), 6.40 (s, 1H), 5.22 (d, *J* = 13.8 Hz, 1H), 5.16 (d, *J* = 13.8 Hz, 1H), 3.06 (q, *J* = 6.8 Hz, 1H), 2.37 (dd, *J* = 16.8, 6.8 Hz, 1H), 2.07–2.12 (m, 1H), 2.00 (dd, *J* = 16.8, 12.8 Hz, 1H), 1.78–1.82 (m, 1H), 1.57–1.70 (m, 4H), 1.23 (s, 3H), 1.14 (d, *J* = 6.9 Hz, 3H), 1.12 (d, *J* = 6.6 Hz, 3H); ^13^C NMR (75 MHz, CDCl_3_) δ_C_: 217.7, 163.9, 150.8, 150.2, 150.2, 145.1, 136.6, 122.0, 122.0, 68.6, 64.3, 62.8, 61.9, 52.0, 50.0, 38.4, 33.5, 28.4, 23.7, 20.0, 18.0; HRMS *m*/*z* 338.1759 [M − H]^−^ (calcd. for C_21_H_24_NO_3_ at *m*/*z* 338.1756).

#### 3.3.3. General Procedure for Synthesis of **15**–**20**

To a solution of SA (15 mg, 0.06 mmol) in DMF (1 mL, freshly distilled from CaH_2_), was added K_2_CO_3_ (25 mg, 0.18 mmol), followed by the addition of dibromoalkane (0.24 mmol). The obtained mixture was then stirred under N_2_ atmosphere at 50 °C for 12 h. Afterwards, the reaction mixture was cooled to room temperature and quenched by brine (5 mL). The mixture was then extracted with DCM (5 mL × 3), washed successively with brine (10 mL × 3) and water (10 mL × 3), dried over Na_2_SO_4_, filtered, and concentrated in vacuo. The residue was purified by silica gel CC (EtOAc/PE ranged from 5:95 to 10:90 depending on the TLC analysis) to yield **15**–**20**.

Compound **15** (21.1 mg), an off-yellow oil, was purified by silica gel CC (EtOAc/PE 10:90) from the reaction residue with 92% yield. ${\left[\alpha \right]}_{D}^{23}$ − 68.4 (c 0.07, DCM); ^1^H NMR (300 MHz, CDCl_3_) δ_H_: 6.27 (s, 1H), 4.07–4.25 (m, 2H), 3.45 (t, *J* = 6.3 Hz, 2H), 3.00 (q, *J* = 6.9 Hz, 1H), 2.35 (dd, *J* = 16.5, 6.6 Hz, 1H), 2.02–2.20 (m, 1H), 1.94–2.00 (m, 2H), 1.76–1.88 (m, 3H), 1.57–1.67 (m, 5H), 1.20 (s, 3H), 1.05–1.15 (m, 6H); ^13^C NMR (75 MHz, CDCl_3_) δ_C_: 217.9, 164.6, 149.8, 137.1, 68.6, 63.5, 62.8, 61.8, 52.0, 50.1, 38.4, 33.5, 33.3, 29.5, 28.4, 27.4, 23.7, 20.0, 18.0; HRMS *m*/*z* 400.1477 [M + NH_4_]^+^ (calcd. for C_19_H_31_BrNO_3_ at *m*/*z* 400.1487).

Compound **16** (21.4 mg), a colorless oil, was purified by silica gel CC (EtOAc/PE 10:90) from the reaction residue with 90% yield. ${\left[\alpha \right]}_{D}^{23}$ − 48.2 (c 0.14, DCM); ^1^H NMR (400 MHz, CDCl_3_) δ_H_: 6.27 (s, 1H), 4.09–4.17 (m, 2H), 3.42 (t, *J* = 6.8 Hz, 2H), 3.00 (q, *J* = 7.2 Hz, 1H), 2.35 (dd, *J* = 16.8, 6.8 Hz, 1H), 2.05–2.07 (m, 1H), 2.00 (dd, *J* = 16.8, 12.8 Hz, 1H), 1.84–1.94 (m, 3H), 1.65–1.73 (m, 5H), 1.50–1.53 (m, 3H), 1.20 (s, 3H), 1.11 (d, *J* = 6.4 Hz, 3H), 1.10 (d, *J* = 7.2 Hz, 3H); ^13^C NMR (100 MHz, CDCl_3_) δ_C_: 218.0, 164.6, 149.6, 137.3, 68.6, 64.1, 62.8, 61.7, 52.0, 50.1, 38.4, 33.6, 33.5, 32.4, 28.4, 27.9, 24.8, 23.7, 20.0, 18.0; HRMS *m*/*z* 395.1221 [M − H]^+^ (calcd. for C_20_H_28_BrO_3_ at *m*/*z* 395.1222).

Compound **17** (22.9 mg), a colorless oil, was purified by silica gel CC (EtOAc/PE 10:90) from the reaction residue with 93% yield. ${\left[\alpha \right]}_{D}^{23}$ − 59.6 (c 0.09, DCM); ^1^H NMR (400 MHz, CDCl_3_) δ_H_: 6.27 (s, 1H), 4.06–4.18 (m, 2H), 3.41 (t, *J* = 6.8 Hz, 2H), 3.00 (q, *J* = 7.6 Hz, 1H ), 2.35 (dd, *J* = 16.8, 6.8 Hz, 1H ), 2.05–2.09 (m, 1H), 1.99 (dd, *J* = 16.8, 12.8 Hz, 1H), 1.84–1.91 (m, 2H), 1.76–1.80 (m, 1H), 1.61–1.71 (m, 6H), 1.40–1.50 (m, 4H), 1.20 (s, 3H), 1.11 (d, *J* = 6.4 Hz, 3H), 1.10 (d, *J* = 7.2 Hz, 3H); ^13^C NMR (100 MHz, CDCl_3_) δ_C_: 218.0, 164.7, 149.5, 137.3, 68.6, 64.3, 62.8, 61.7, 52.0, 50.1, 38.4, 33.9, 33.5, 32.7, 28.6, 28.4, 27.9, 25.4, 23.7, 20.0, 18.0; HRMS *m*/*z* 409.1381 [M − H]^+^ (calcd. for C_21_H_30_BrO_3_ at *m*/*z* 409.1379).

Compound **18** (23.2 mg), an off-yellow oil, was purified by silica gel CC (EtOAc/PE 5:95) from the reaction residue with 91% yield. ${\left[\alpha \right]}_{D}^{23}$ − 79.1 (c 0.39, DCM); ^1^H NMR (400 MHz, CDCl_3_) δ_H_: 6.27 (s, 1H), 4.07–4.15 (m, 2H), 3.41 (t, *J* = 6.8 Hz, 2H), 3.00 (q, *J* = 6.8 Hz, 1H ), 2.35 (dd, *J* = 16.8, 6.8 Hz, 1H), 2.05–2.09 (m, 1H), 1.99 (dd, *J* = 16.8, 12.8 Hz, 1H), 1.78–1.89 (m, 4H), 1.64–1.68 (m, 5H), 1.50–1.60 (m, 2H), 1.35–1.38 (m, 4H), 1.20 (s, 3H), 1.11 (d, *J* = 6.4 Hz, 3H), 1.10 (d, *J* = 7.2 Hz, 3H); ^13^C NMR (100 MHz, CDCl_3_) δ_C_: 218.0, 164.7, 149.4, 137.4, 68.6, 64.4, 62.8, 61.7, 52.0, 50.1, 38.4, 34.0, 33.5, 32.8, 28.6, 28.5, 28.4, 28.1, 26.0, 23.7, 20.0, 18.0; HRMS m/z 423.1527 [M − H]^+^ (calcd. for C_22_H_32_BrO_3_ at *m*/*z* 423.1535).

Compound **19** (23.4 mg), an off-yellow oil, was purified by silica gel CC (EtOAc/PE 5:95) from the reaction residue with 89% yield. ${\left[\alpha \right]}_{D}^{23}$ − 124.5 (c 0.96, DCM); ^1^H NMR (400 MHz, CDCl_3_) δ_H_: 6.27 (s, 1H), 4.05–4.17 (m, 2H), 3.40 (t, *J* = 6.8 Hz, 2H), 3.00 (q, *J* = 7.2 Hz, 1H ), 2.35 (dd, *J* = 16.4, 6.8 Hz, 1H ), 2.05–2.09 (m, 1H), 1.99 (dd, *J* = 16.4, 12.4, 1H), 180–1.89 (m, 2H), 1.57–1.79 (m, 9H), 1.32–1.41 (m, 6H), 1.20 (s, 3H), 1.11 (d, *J* = 6.4 Hz, 3H), 1.10 (d, *J* = 7.2 Hz, 3H); ^13^C NMR (100 MHz, CDCl_3_) δ_C_: 218.0, 164.8, 149.4, 137.4, 68.6, 64.5, 62.8, 61.7, 52.0, 50.1, 38.4, 34.1, 33.5, 32.9, 29.2, 28.7, 28.7, 28.4, 28.2, 26.0, 23.7, 20.0, 18.0; HRMS *m*/*z* 439.1854 [M + H]^+^ (calcd. for C_23_H_36_BrO_3_ at *m*/*z* 439.1848).

Compound **20** (24.4 mg), an off-yellow oil, was purified by silica gel CC (EtOAc/PE 5:95) from the reaction residue with 90% yield. ${\left[\alpha \right]}_{D}^{23}$ − 108.7 (c 0.08, DCM); ^1^H NMR (400 MHz, CDCl_3_) δ_H_: 6.26 (s, 1H), 4.05–4.16 (m, 2H), 3.40 (t, *J* = 6.8 Hz, 2H), 3.00 (q, *J* = 6.8 Hz, 1H), 2.34 (dd, *J* = 16.8, 6.8 Hz, 1H ), 2.05–2.09 (m, 1H), 1.99 (dd, *J* = 16.8, 12.8 Hz, 1H), 175–1.89 (m, 3H), 1.53–1.67 (m, 7H), 1.30–1.44 (m, 9H), 1.20 (s, 3H), 1.11 (d, *J* = 6.4 Hz, 3H), 1.10 (d, *J* = 7.2 Hz, 3H); ^13^C NMR (100 MHz, CDCl_3_) δ_C_: 218.0, 164.8, 149.4, 137.4, 68.6, 64.6, 62.8, 61.7, 52.0, 50.1, 38.4, 34.2, 33.5, 32.9, 29.4, 29.3, 28.8, 28.7, 28.4, 28.2, 26.1, 23.7, 20.0, 18.0; HRMS *m*/*z* 453.2013 [M + H]^+^ (calcd. for C_24_H_38_BrO_3_ at *m*/*z* 453.2004).

#### 3.3.4. General Procedure for Syntheses of **21**–**26**

To a solution of SA (20 mg, 0.08 mmol) in DCM (1 mL, freshly distilled from CaH_2_), was added oxalyl chloride (17.5 μL, 0.20 mmol). The obtained mixture was then stirred under N_2_ atmosphere at room temperature for 1 h. Afterwards, the reaction mixture was evaporated in vacuo to remove the DCM and left oxalyl chloride to give the SA acid chloride, which was used directly for the next step directly. To the flask containing SA acid chloride was added 1 mL freshly distilled DCM, followed by diaminoalkane (0.4 mmol). The mixture was then stirred under N_2_ atmosphere at room temperature for 10h and quenched with brine (5 mL). The mixture was then extracted with DCM (5 mL x 3), washed successively with brine (10 mL × 3) and water (10 mL × 3), dried over Na_2_SO_4_, filtered, and concentrated in vacuo. The residue was purified by silica gel CC (MeOH/DCM 3:97) to yield **21**–**26**.

Compound **21** (21.6 mg, 89% isolated yield) was obtained as a colorless oil. ${\left[\alpha \right]}_{D}^{23}$ − 55.9 (c 0.07, DCM); ^1^H NMR (300 MHz, CDCl_3_) δ_H_: 6.94 (t, *J* = 5.4 Hz, 1H), 5.80 (s, 1H), 3.39 (m, 2H), 3.05 (q, *J* = 7.2 Hz, 1H), 2.83 (t, *J* = 6.3 Hz, 2H), 2.32 (dd, *J* = 16.8, 6.9 Hz, 1H), 2.08 (m, 1H), 1.97 (dd, *J* = 16.8, 12.3 Hz, 1H), 1.61–1.77 (m, 5H), 1.52–1.59 (m, 2H), 1.16 (s, 3H), 1.09 (d, *J* = 6.6 Hz, 3H), 1.06 (d, *J* = 7.2 Hz, 3H); ^13^C NMR (75 MHz, CDCl_3_) δ_C_: 218.4, 165.3, 141.4, 141.3, 68.7, 62.7, 61.8, 52.0, 50.1, 40.1, 38.6, 38.1, 33.4, 31.5, 28.3, 24.3, 20.0, 17.8; HRMS m/z 305.2228 [M + H]^+^ (calcd. for C_18_H_29_N_2_O_2_ at *m*/*z* 305.2229).

Compound **22** (24.5 mg, 92% isolated yield) was obtained as an off-yellow oil. ${\left[\alpha \right]}_{D}^{23}$ − 35.1 (c 0.13, DCM); ^1^H NMR (300 MHz, CDCl_3_) δ_H_: 5.79 (t, *J* = 6.0, 1H), 5.74 (s, 1H), 3.29 (m, 2H), 3.07 (q, *J* = 6.9 Hz, 1H), 2.68 (t, *J* = 6.9 Hz, 2H), 2.34 (dd, *J* = 16.8, 6.6 Hz, 1H), 2.09 (m, 1H), 1.97 (dd, *J* = 16.8, 12.3 Hz, 1H), 1.49–1.76 (m, 11H), 1.17 (s, 3H), 1.08 (d, *J* = 6.3 Hz, 3H), 1.06 (d, *J* = 7.2 Hz, 3H); ^13^C NMR (75 MHz, CDCl_3_) δ_C_: 218.3, 165.2, 141.5, 140.9, 68.7, 62.7, 61.9, 52.1, 50.1, 42.1, 39.4, 38.6, 33.3, 33.3, 29.6, 28.2, 24.4, 24.3, 19.9, 17.7; HRMS *m*/*z* 333.2532 [M + H]^+^ (calcd. for C_20_H_33_N_2_O_2_ at *m*/*z* 333.2542).

Compound **23** (25.4 mg, 88% isolated yield) was obtained as an off-yellow oil. ${\left[\alpha \right]}_{D}^{23}$ − 97.0 (c 0.29, DCM); ^1^H NMR (300 MHz, CDCl_3_) δ_H_: 5.78 (t, *J* = 5.4 Hz, 1H), 5.74 (s, 1H), 3.26 (m, 2H), 3.06 (q, *J* = 6.9 Hz, 1H), 2.66 (t, *J* = 6.6 Hz, 2H), 2.33 (dd, *J* = 16.8, 6.9 Hz, 1H), 2.08 (m, 1H), 1.96 (dd, *J* = 16.8, 12.6 Hz, 1H), 1.62–1.77 (m, 3H), 1.40–1.59 (m, 6H), 1.30 (br.s, 6H), 1.16 (s, 3H), 1.09 (d, *J* = 6.6 Hz, 3H), 1.05 (d, *J* = 7.2 Hz, 3H); ^13^C NMR (75 MHz, CDCl_3_) δ_C_: 218.3, 165.1, 141.5, 140.8, 68.7, 62.7, 61.9, 52.1, 50.1, 42.1, 39.5, 38.6, 33.5, 33.3, 29.7, 29.2, 28.2, 27.0, 26.8, 24.3, 19.9, 17.7; HRMS *m*/*z* 361.2852 [M + H]^+^ (calcd. for C_22_H_37_N_2_O_2_ at *m*/*z* 361.2855).

Compound **24** (27.8 mg, 93% isolated yield) was obtained as a colorless oil. ${\left[\alpha \right]}_{D}^{23}$ − 110.8 (c 0.53, DCM); ^1^H NMR (300 MHz, CDCl_3_) δ_H_: 5.74 (m, 2H), 3.26 (m, 2H), 3.07 (q, *J* = 7.2 Hz, 1H), 2.68 (t, *J* = 6.6 Hz, 2H), 2.34 (dd, *J* = 16.8, 6.9 Hz, 1H), 2.08 (m, 1H), 1.96–2.02 (m, 1H), 1.63–1.77 (m, 3H), 1.41–1.60 (m, 6H), 1.29 (br.s, 8H), 1.17 (s, 3H), 1.09 (d, *J* = 6.6 Hz, 3H), 1.06 (d, *J* = 7.2 Hz, 3H); ^13^C NMR (75 MHz, CDCl_3_) δ_C_: 218.3, 165.1, 141.6, 140.8, 68.7, 62.7, 61.9, 52.1, 50.1, 42.1, 39.5, 38.6, 33.4, 33.3, 29.7, 29.4, 29.3, 28.2, 27.0, 26.9, 24.4, 19.9, 17.7; HRMS *m*/*z* 375.2997 [M + H]^+^ (calcd. for C_23_H_39_N_2_O_2_ at *m*/*z* 375.3012).

Compound **25** (27.4 mg, 85% isolated yield) was obtained as an off-yellow oil. ${\left[\alpha \right]}_{D}^{23}$ − 128.5 (c 0.38, DCM); ^1^H NMR (300 MHz, CDCl_3_) δ_H_: 5.73 (m, 2H), 3.26 (m, 2H), 3.07 (q, *J* = 7.2 Hz, 1H), 2.67 (t, *J* = 6.9 Hz, 2H), 2.34 (dd, *J* = 16.8, 6.6Hz, 1H), 2.08 (m, 1H), 1.97 (dd, *J* = 16.8, 12.6 Hz, 1H), 1.38–1.70 (m, 9H), 1.26 (br.s, 12H), 1.17 (s, 3H), 1.09 (d, *J* = 6.3 Hz, 3H), 1.06 (d, *J* = 7.2 Hz, 3H); ^13^C NMR (75 MHz, CDCl_3_) δ_C_: 218.3, 165.1, 141.6, 140.8, 68.7, 62.7, 61.9, 52.1, 50.1, 42.3, 39.6, 39.6, 38.6, 33.7, 33.3, 29.7, 29.6, 29.6, 29.4, 28.2, 27.1, 27.0, 24.4, 19.9, 17.7; HRMS m/z 403.3310 [M + H]^+^ (calcd. for C_25_H_43_N_2_O_2_ at *m*/*z* 403.3325).

Compound **26** (32.0 mg, 93% isolated yield) was calculated as an off-yellow oil. ${\left[\alpha \right]}_{D}^{23}$ − 152.1 (c 0.17, DCM); ^1^H NMR (300 MHz, CDCl_3_) δ_H_: 5.69–5.74 (m, 2H), 3.27 (m, 2H), 3.07 (q, *J* = 7.2 Hz, 1H), 2.67 (t, *J* = 6.9 Hz, 2H), 2.34 (dd, *J* = 16.8, 6.9 Hz, 1H), 2.09 (m, 1H), 1.97 (dd, *J* = 16.8, 12.3 Hz, 1H), 1.41–1.73 (m, 9H), 1.25 (br.s, 16H), 1.17 (s, 3H), 1.09 (d, *J* = 6.6 Hz, 3H), 1.06 (d, *J* = 7.2 Hz, 3H); ^13^C NMR (75 MHz, CDCl_3_) δ_C_: 218.3, 165.1, 141.6, 140.8, 68.7, 62.7, 61.9, 52.1, 50.1, 42.3, 39.6, 38.6, 33.7, 33.5, 33.3, 29.8, 29.7, 29.7, 29.6, 29.6, 29.4, 28.2, 27.1, 27.0, 24.4, 19.9, 17.7; HRMS m/z 431.3620 [M + H]^+^ (calcd. for C_27_H_47_N_2_O_2_ at *m*/*z* 431.3638).

### 3.4. Antifouling Assay

The antifouling activity was examined using cyprids of the barnacle *B. amphitrite* Darwin according to the method reported previously [19]. Briefly, adults of *B. amphitrite*, collected from the intertidal zone in Hong Kong, were exposed to air for more than 6 h, and were then placed in a container filled with 0.22 µm filtered seawater (FSW) to release nauplii. The collected nauplii were raised to cyprid stage using the method described by Thiyagarajan et al. [30]. Fresh cyprids were used in the test. Larval settlement assays were examined using 24-well polystyrene plates. The tested samples were first dissolved with small amount of dimethyl sulfoxide (DMSO) and then diluted with FSW to achieve final concentrations at 25.0, 12.5, 6.25, 3.13, 1.57, 0.78, 0.39, 0.19, 0.1, and 0.05 µg/mL. About 15−20 competent larvae were added to each well containing 1 mL of test solution in triplicate, and wells containing only FSW, DMSO, and larvae served as a control. The plates were incubated at 25 °C for 48 h. The effects of the test samples against biofouling were determined by examining the plates under a microscope to count settled, unsettled, and swimming larvae, and where appropriate, potential toxic effects were recorded. The number of settled larvae was expressed as a percentage of the total number of larvae per well. Three replicates of each treatment were performed. A concentration-response curve was then made and a trend line was constructed for each compound. EC_50_ was calculated as the concentration where 50% of the larval individual was inhibited to settle as compared to the control while LC_50_ was calculated as the concentration where 50% of the larval population was dead. In this study, the highest concentration tested for the toxicity of each compound toward the *B. amphitrite* was fixed at 25 µg/mL, partially due to limited amounts of compounds available after being used for the EC_50_ test, together with the unstable status and the limited availability of barnacle *B. amphitrite* which is highly sensitive to climate and environment changes. For the purposes of this study, adults of the barnacle *B. amphitrite* were scraped off from the concrete posts at a pier of Pak Sha Wan, Kowloon, Hong Kong (22°21′45″N, 114°15′35″E), where only limited quantity of *B. amphitrite* could be collected during the period of our experiment because of the relatively hot weather and artificial disturbing of nearby construction work.

## 4. Conclusions

As part of our continuous search for non-toxic yet high efficiency antifoulants from marine resources, the chemical composition of the gorgonian *S. suberosa* has been systematically investigated, leading to the discovery of SA, which not only significantly inhibited the settlement of the barnacle *B. amphitrite* (EC_50_ 1.25 ± 0.14 μg/mL, LC_50_ > 25 μg/mL), but also showed rich abundance among the metabolites. This discovery allowed us to hypothesize that SA might be the primary defense chemical produced by the gorgonian as a natural antifoulant. Therefore, the structure-activity relationships of SA were studied to identify the bioactive functional groups responsible for the antifouling effect, followed by the rational design, synthesis, and antifouling testing of SA derivatives, aiming to obtain more potent antifoulant using SA as a lead. Our results indicated, for the first time, that the ketone carbonyl and the double bond of SA were essential groups accounting for the antifouling effect of SA, while the acid group slightly influenced biological activity. Based on these preliminary structure-activity relationships, two series of SA derivatives (one being benzyl esters of SA, and another being SA derivatives containing methylene chains of various lengths), were then designed and synthesized. Antifouling testing revealed that all benzyl esters of SA exhibited comparable or more potent antifouling effects compared with SA, with compound **5**, which contained a non-substituted benzyl ring, showing the most potent antifouling activity (EC_50_ 0.30 ± 0.10 μg/mL, LC_50_ > 25 μg/mL). Furthermore, the results also indicated that none of the SA derivatives containing a methylene chain exhibited better antifouling effects than SA, while the length of the methylene chain differently influenced the antifouling effects of SA derivatives depending on the functional group at the opposite site of the methylene chain.

## Figures and Tables

**Table 1:** Antifouling activities of SA and **1**–**4** against the settlement of *B. amphitrite*.

Compound	EC_50_ (μg/mL)	LC_50_ (μg/mL)
**SA**	1.25 ± 0.14 ^c^	>25
**1**	3.16 ± 0.18 ^b^	>25
**2**	10.01 ± 0.13 ^a^	>25
**3**	>25	>25
**4**	>25	>25

The data are reported as average of triplicate ± SD; the superscripts a, b, and c are used to distinguish the statistical difference among the values of EC_50_, with the different lowercase letters indicating significant statistical difference (*p* < 0.05).

**Table 2:** Antifouling activities of **5**–**14** against the settlement of *B. amphitrite*.

Compound	R	EC_50_ (μg/mL)	LC_50_ (μg/mL)
**5**	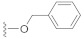	0.30 ± 0.10 ^c^	>25
**6**	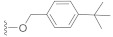	1.20 ± 0.17 ^b^	>25
**7**	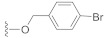	1.26 ± 0.11 ^b^	>25
**8**	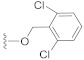	0.40 ± 0.12 ^c^	>25
**9**	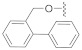	1.28 ± 0.17 ^b^	>25
**10**	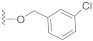	0.65 ± 0.13 ^c^	>25
**11**	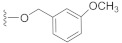	0.61 ± 0.13 ^c^	>25
**12**	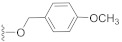	2.50 ± 0.18 ^a^	>25
**13**	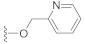	0.32 ± 0.11 ^c^	>25
**14**	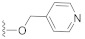	0.63 ± 0.12 ^c^	>25

The data are reported as average of triplicate ± SD; the superscripts a, b, and c are used to distinguish the statistical difference among the values of EC_50_, with the different lowercase letters indicating significant statistical difference (*p* < 0.05).

**Table 3:** Antifouling activities of **15**–**16** against the settlement of *B. amphitrite*.

Compound	R	EC_50_ (μg/mL)	LC_50_ (μg/mL)
**15**	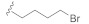	2.05 ± 0.16 ^e^	>25
**16**	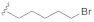	2.48 ± 0.11 ^d^	>25
**17**	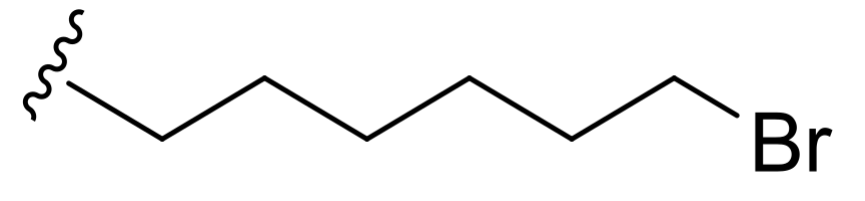	2.75 ± 0.13 ^d^	>25
**18**	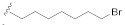	7.51 ± 0.22 ^b^	>25
**19**	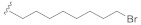	>25	>25
**20**	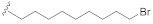	>25	>25
**21**	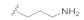	>25	>25
**22**	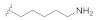	>25	>25
**23**	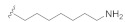	>25	>25
**24**	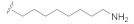	>25	>25
**25**	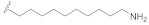	20.00 ± 0.15 ^a^	>25
**26**	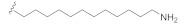	4.50 ± 0.10 ^c^	>25

The data are reported as average of triplicate ± SD; the superscripts a, b, c, d, and e are used to distinguish the statistical difference among the values of EC_50_, with the different lowercase letters indicating significant statistical difference (*p* < 0.05).

## Supplementary Materials

The following are available online at http://www.mdpi.com/1660-3397/17/2/101/s1, Figure S1, the ^1^H, ^13^C NMR spectra of SA, **1**–**26**, HSQC and NOESY spectra of **4**, and the HRMS spectra of **1-26**.
