# Supplementary material for: Isolation and Structure-Activity Relationship of Subergorgic Acid and Synthesis of Its Derivatives as Antifouling Agent
Source: Mar Drugs. 2019 Feb 6;17(2):101. doi: 10.3390/md17020101 (PMC6410164; doi:10.3390/md17020101)

Supporting information for journal *Marine drugs*

Isolation, Structure-activity Relationship of Subergorgic Acid and Synthesis of Its Derivatives as Antifouling Agent

Jun Zhang [1,3*], Wei Ling [1], Zhiqiang Yang [1], Yan Liang [1], Linyan Zhang [1], Can Guo [1], Kailing Wang [2], Balian Zhong [1], Shihai Xu [3,*] and Ying Xu [2,*]

[1] National Engineering Research Center of Navel Orange, Gannan Normal University, Ganzhou, 341000, China; lw791010@163.com (W.L.); 15707975909@163.com (Z.Y.); zjzzf2011@gmail.com (Y.L.); 18573488362@139.com (L.Z.); 15225960215@139.com (C.G.); bal.zh@163.com (B.Z.)

[2] College of Life Science and Oceanography, Shenzhen University, Shenzhen, 518000, China; 15216101485@163.com (K.W.)

[3] Department of Chemistry, Jinan University, Guangzhou, 510000, China.

* Correspondence: bri71527152@outlook.com (J.Z.); boxuying@szu.edu.cn (Y.X.); txush@jnu.edu.cn (S.X.); Tel.: +86-797-8393068 (J.Z.); +86-755-26958849 (Y.X.)

Table of contends

**Figure S1.** Key NOE correlations of **4**.	Page 3	**Figure S43**. ^{13}C NMR (100 MHz, $CDCl_3$) of compound **19**	45
**Figure S2.** ^{1}H NMR (400 MHz, $CDCl_3$) of compound **SA**	4	**Figure S44**. ^{1}H NMR (400 MHz, $CDCl_3$) of compound **20**	46
**Figure S3.** ^{13}C NMR (100 MHz, $CDCl_3$) of compound **SA**	5	**Figure S45**. ^{13}C NMR (100 MHz, $CDCl_3$) of compound **20**	47
**Figure S4.** ^{1}H NMR (300 MHz, $CDCl_3$) of compound **1**	6	**Figure S46**. ^{1}H NMR (300 MHz, $CDCl_3$) of compound **21**	48
**Figure S5.** ^{13}C NMR (75 MHz, $CDCl_3$) of compound **1**	7	**Figure S47**. ^{13}C NMR (75 MHz, $CDCl_3$) of compound **21**	49
**Figure S6.** ^{1}H NMR (300 MHz, $CDCl_3$) of compound **2**	8	**Figure S48**. ^{1}H NMR (300 MHz, $CDCl_3$) of compound **22**	50
**Figure S7.** ^{13}C NMR (75 MHz, $CDCl_3$) of compound **2**	9	**Figure S49**. ^{13}C NMR (75 MHz, $CDCl_3$) of compound **22**	51
**Figure S8.** ^{1}H NMR (400 MHz, $CDCl_3$) of compound **3**	10	**Figure S50**. ^{1}H NMR (300 MHz, $CDCl_3$) of compound **23**	52
**Figure S9.** ^{13}C NMR (100 MHz, $CDCl_3$) of compound **3**	11	**Figure S51**. ^{13}C NMR (75 MHz, $CDCl_3$) of compound **23**	53
**Figure S10.** ^{1}H NMR (300 MHz, $CDCl_3$) of compound **4**	12	**Figure S52**. ^{1}H NMR (300 MHz, $CDCl_3$) of compound **24**	54
**Figure S11.** ^{13}C NMR (75 MHz, $CDCl_3$) of compound **4**	13	**Figure S53**. ^{13}C NMR (75 MHz, $CDCl_3$) of compound **24**	55
**Figure S12.** HSQC spectrum of compound **4**	14	**Figure S54**. ^{1}H NMR (300 MHz, $CDCl_3$) of compound **25**	56
**Figure S13.** NOESY spectrum of compound **4**	15	**Figure S55**. ^{13}C NMR (75 MHz, $CDCl_3$) of compound **25**	57
**Figure S14.** ^{1}H NMR (400 MHz, $CDCl_3$) of compound **5**	16	**Figure S56**. ^{1}H NMR (300 MHz, $CDCl_3$) of compound **26**	58
**Figure S15.** ^{13}C NMR (100 MHz, $CDCl_3$) of compound **5**	17	**Figure S57**. ^{13}C NMR (75 MHz, $CDCl_3$) of compound **26**	59
**Figure S16.** ^{1}H NMR (300 MHz, $CDCl_3$) of compound **6**	18	**Figure S58**. HRMS of compound **4**	60
**Figure S17.** ^{13}C NMR (75 MHz, $CDCl_3$) of compound **6**	19	**Figure S59** HRMS of compound **5**	61
**Figure S18.** ^{1}H NMR (300 MHz, $CDCl_3$) of compound **7**	20	**Figure S60**. HRMS of compound **8**	62
**Figure S19.** ^{13}C NMR (75 MHz, $CDCl_3$) of compound **7**	21	**Figure S61**. HRMS of compound **10**	63
**Figure S20.** ^{1}H NMR (300 MHz, $CDCl_3$) of compound **8**	22	**Figure S62**. HRMS of compound **11**	64
**Figure S21.** ^{13}C NMR (75 MHz, $CDCl_3$) of compound **8**	23	**Figure S63**. HRMS of compound **13**	65
**Figure S22.** ^{1}H NMR (300 MHz, $CDCl_3$) of compound **9**	24	**Figure S64**. HRMS of compound **14**	66
**Figure S23.** ^{13}C NMR (75 MHz, $CDCl_3$) of compound **9**	25	**Figure S65**. HRMS of compound **1**	67
**Figure S24.** ^{1}H NMR (300 MHz, $CDCl_3$) of compound **10**	26	**Figure S66.** HRMS of compound **2**	68
**Figure S25.** ^{13}C NMR (75 MHz, $CDCl_3$) of compound **10**	27	**Figure S67**. HRMS of compound **3**	69
**Figure S26.** ^{1}H NMR (300 MHz, $CDCl_3$) of compound **11**	28	**Figure S68**. HRMS of compound **6**	70
**Figure S27.** ^{13}C NMR (75 MHz, $CDCl_3$) of compound **11**	29	**Figure S69**. HRMS of compound **7**	71
**Figure S28.** ^{1}H NMR (300 MHz, $CDCl_3$) of compound **12**	30	**Figure S70**. HRMS of compound **9**	72
**Figure S29.** ^{13}C NMR (75 MHz, $CDCl_3$) of compound **12**	31	**Figure S71**. HRMS of compound **12**	73
**Figure S30.** ^{1}H NMR (300 MHz, $CDCl_3$) of compound **13**	32	**Figure S72**. HRMS of compound **15**	74
**Figure S31.** ^{13}C NMR (75 MHz, $CDCl_3$) of compound **13**	33	**Figure S73.** HRMS of compound **16**	75
**Figure S32.** ^{1}H NMR (300 MHz, $CDCl_3$) of compound **14**	34	**Figure S74**. HRMS of compound **17**	76

**Figure S33.** ^{13}C NMR (75 MHz, $CDCl_3$) of compound **14**	35	**Figure S75**. HRMS of compound **18**	77
**Figure S34.** ^{1}H NMR (300 MHz, $CDCl_3$) of compound **15**	36	**Figure S76**. HRMS of compound **19**	78
**Figure S35.** ^{13}C NMR (75 MHz, $CDCl_3$) of compound **15**	37	**Figure S77**. HRMS of compound **20**	79
**Figure S36.** ^{1}H NMR (400 MHz, $CDCl_3$) of compound **16**	38	**Figure S78**. HRMS of compound **21**	80
**Figure S37.** ^{13}C NMR (100 MHz, $CDCl_3$) of compound **16**	39	**Figure S79**. HRMS of compound **22**	81
**Figure S38.** ^{1}H NMR (400 MHz, $CDCl_3$) of compound **17**.	40	**Figure S80** HRMS of compound **23**	82
**Figure S39.** ^{13}C NMR (100 MHz, $CDCl_3$) of compound **17**	41	**Figure S81**. HRMS of compound **24**	83
**Figure S40.** ^{1}H NMR (400 MHz, $CDCl_3$) of compound **18**	42	**Figure S82**. HRMS of compound **25**	84
**Figure S41**. ^{13}C NMR (100 MHz, $CDCl_3$) of compound **18**	43	**Figure S83**. HRMS of compound **26**	85
**Figure S42**. ^{1}H NMR (400 MHz, $CDCl_3$) of compound **19**	44		

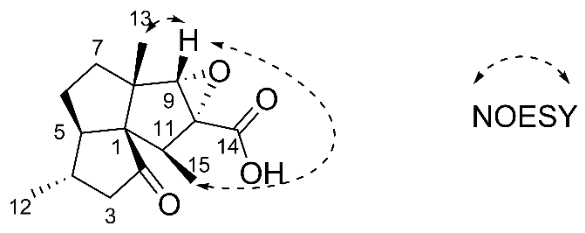

Figure S1. Key NOE correlations of **4**.

Figure S2. ^{1}H NMR (400 MHz, $CDCl_3$) of compound **SA**

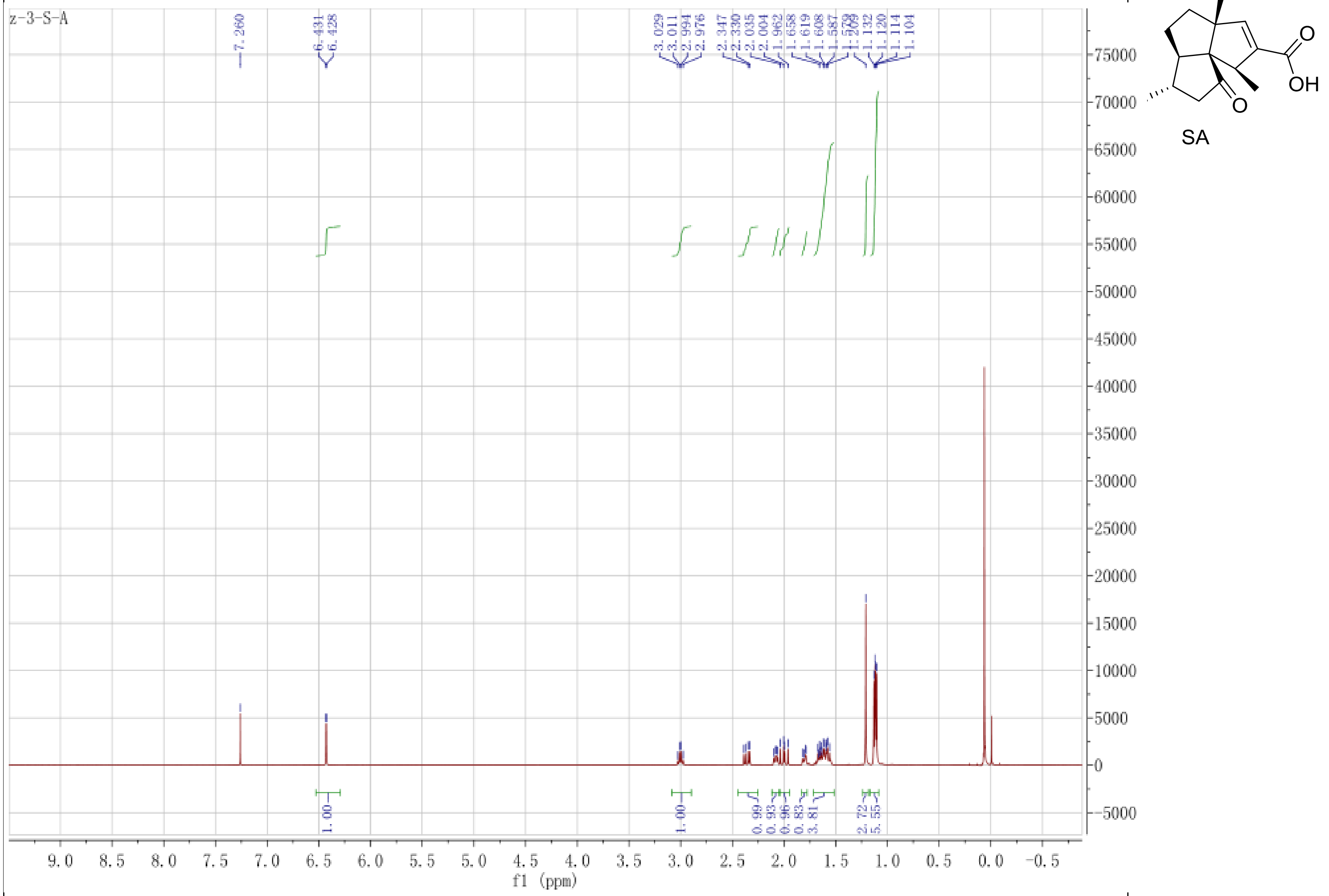

Figure S3. ^{13}C NMR (100 MHz, $CDCl_3$) of compound **SA**

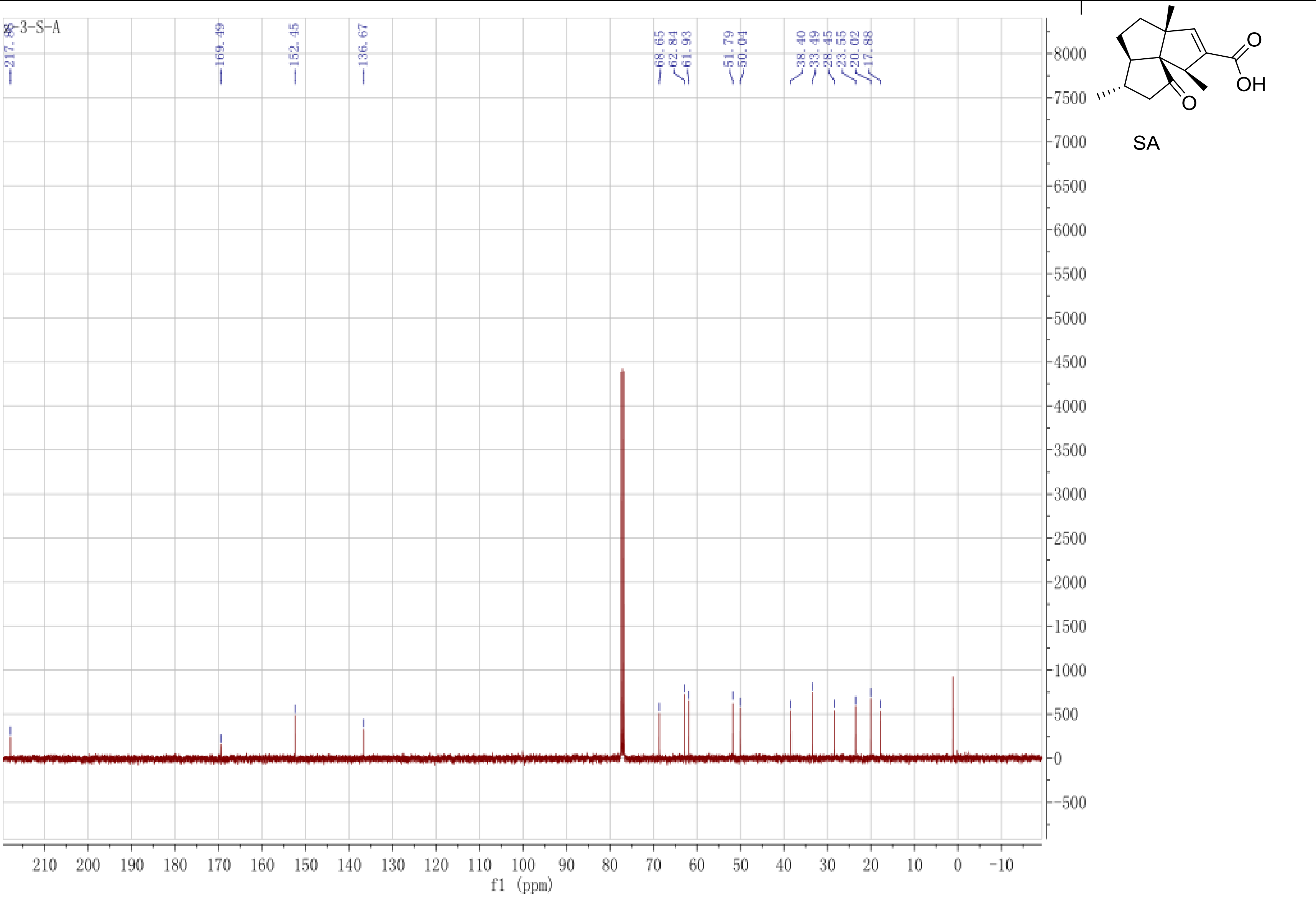

Figure S4. ^{1}H NMR (300 MHz, $CDCl_3$) of compound **1**

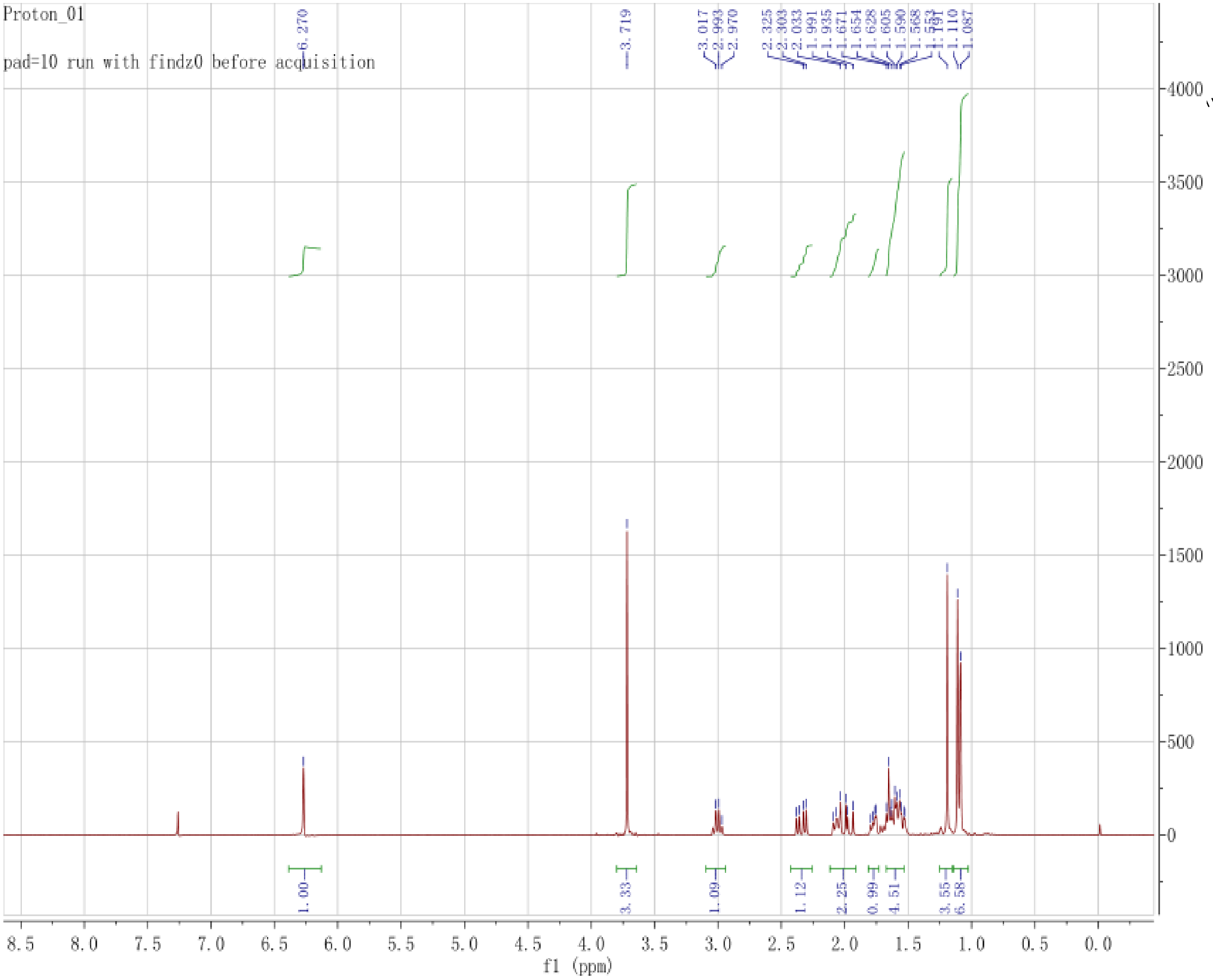

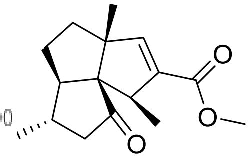

1

Figure S5. ^{13}C NMR (75 MHz, $CDCl_3$) of compound **1**

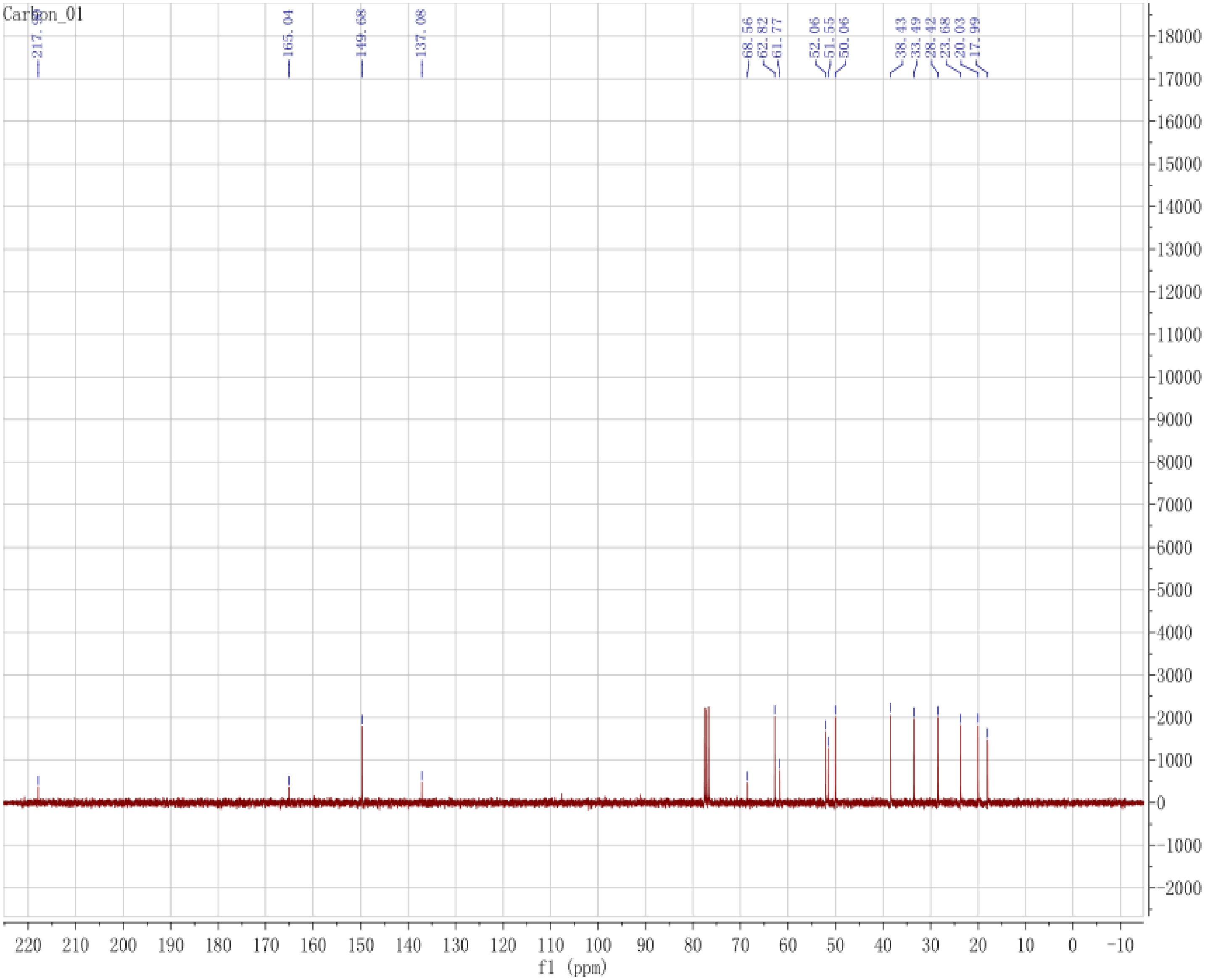

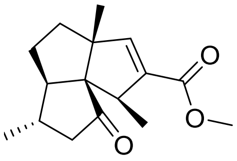

1

Figure S6. ^{1}H NMR (300 MHz, $CDCl_3$) of compound **2**

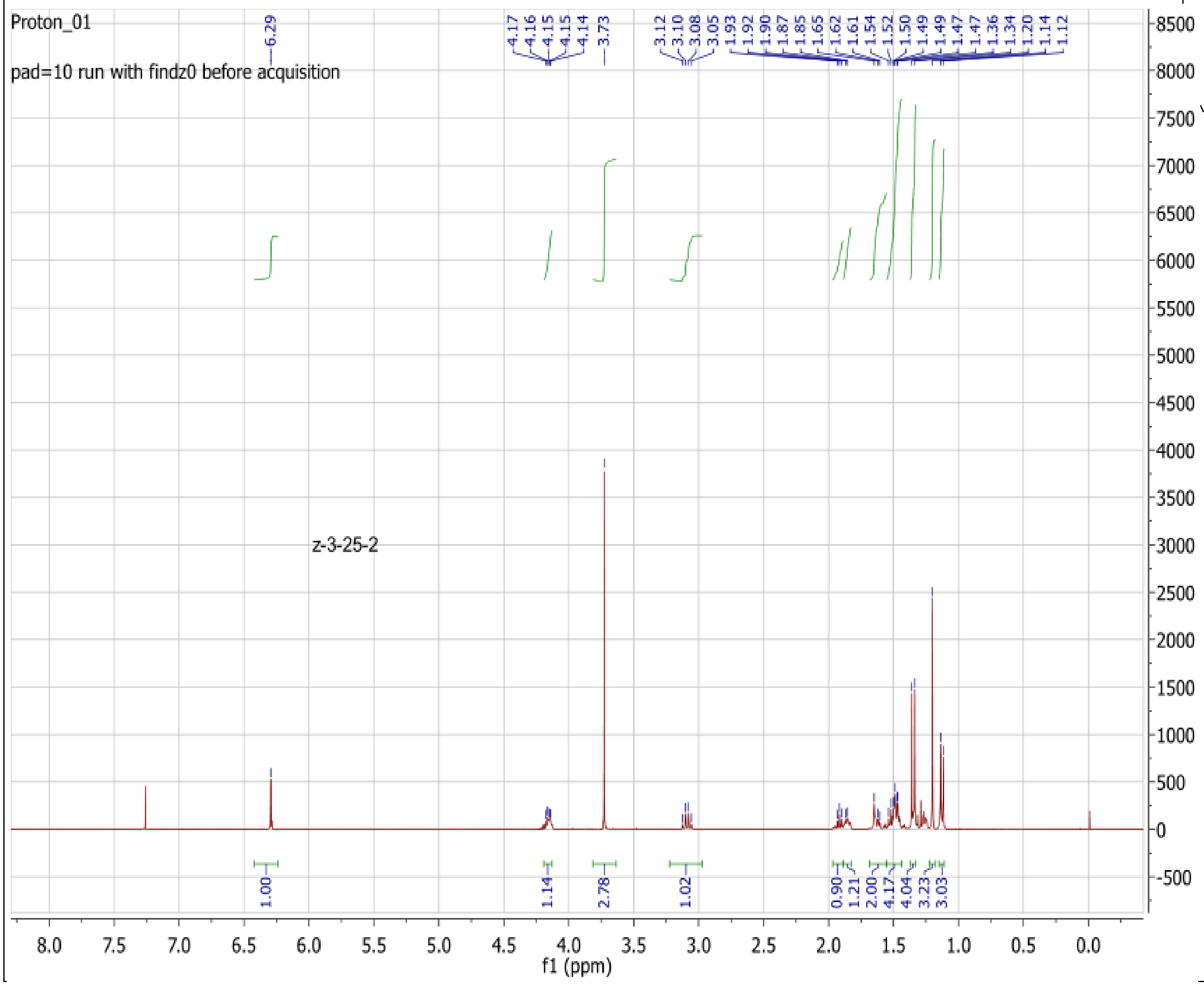

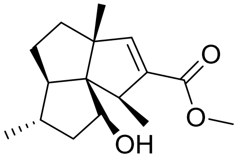

2

Figure S7. ^{13}C NMR (75 MHz, $CDCl_3$) of compound **2**

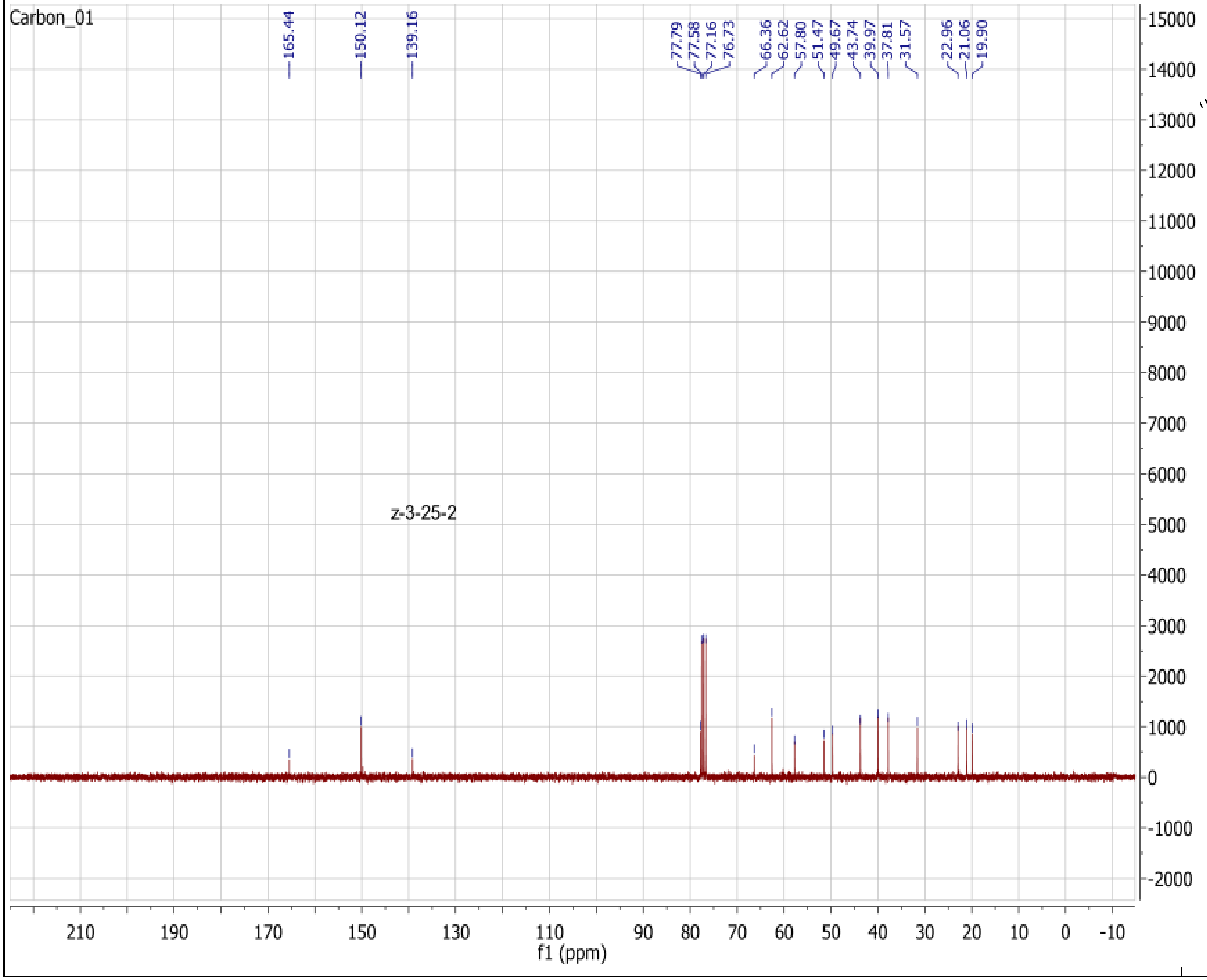

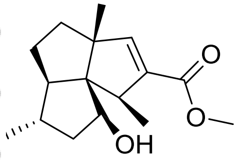

2

Figure S8. ^{1}H NMR (400 MHz, $CDCl_3$) of compound **3**

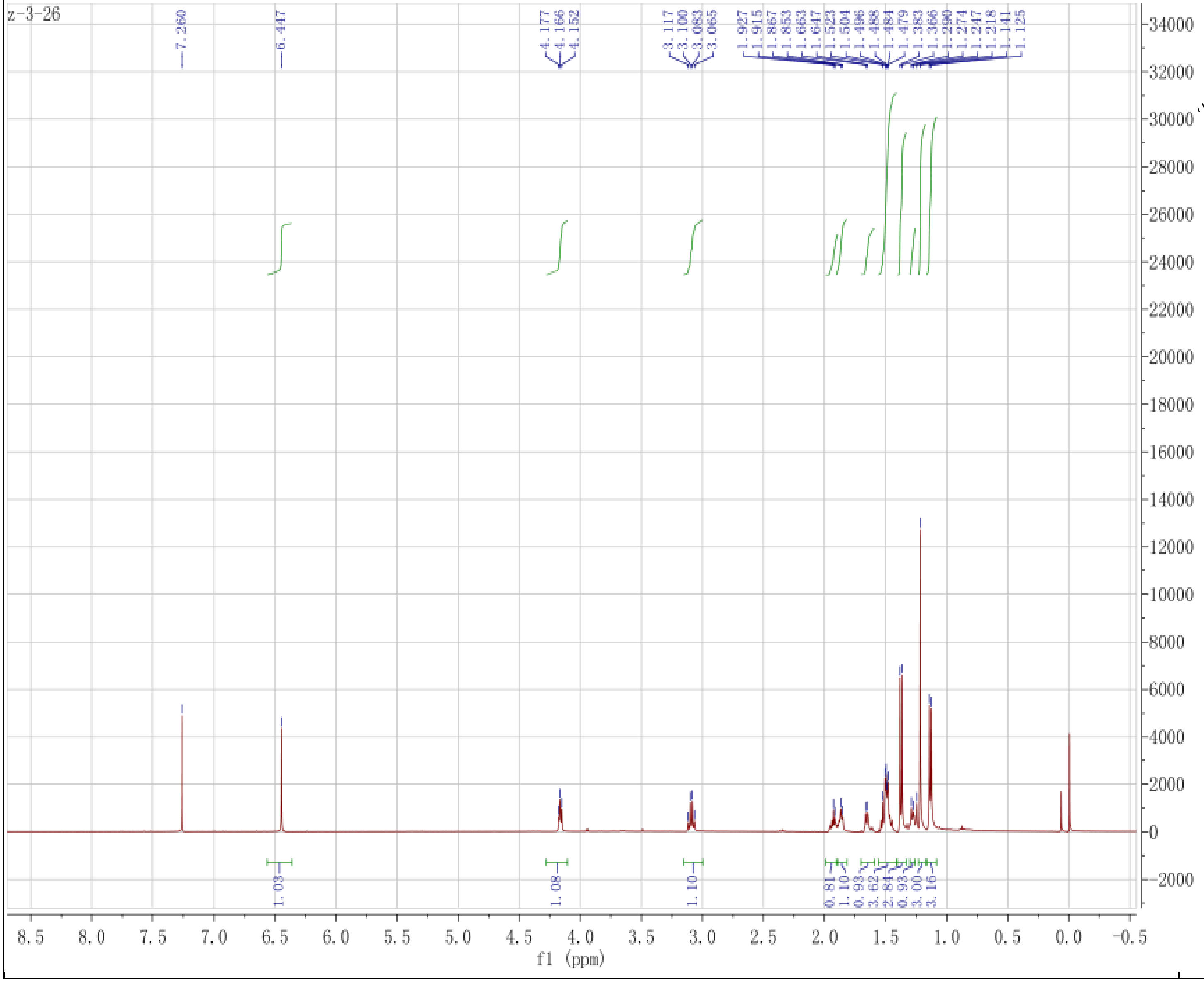

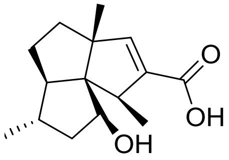

3

Figure S9. ^{13}C NMR (100 MHz, $CDCl_3$) of compound **3**

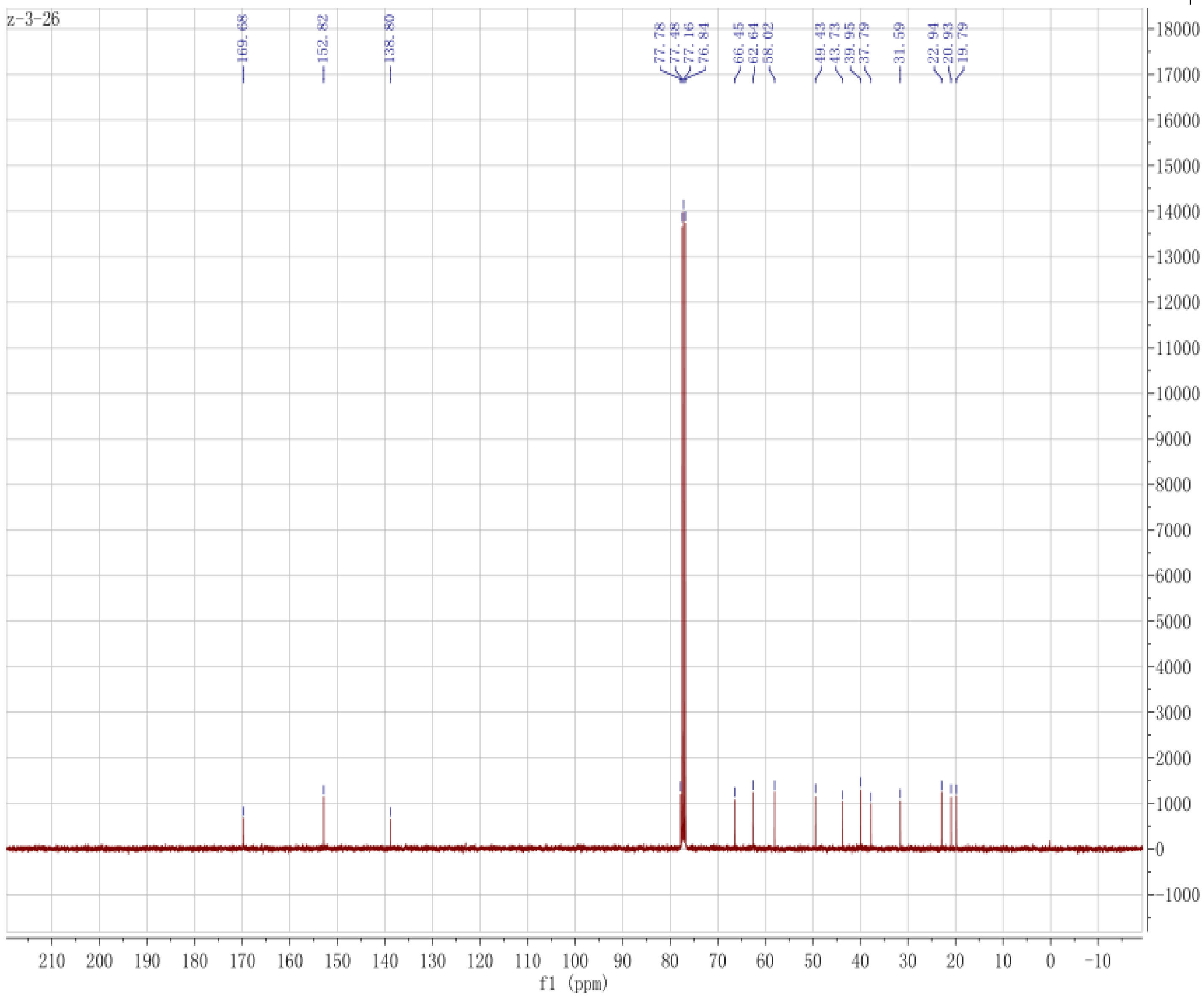

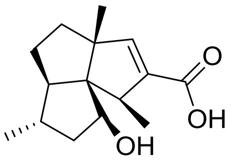

3

Figure S10. ^{1}H NMR (300 MHz, $CDCl_3$) of compound **4**

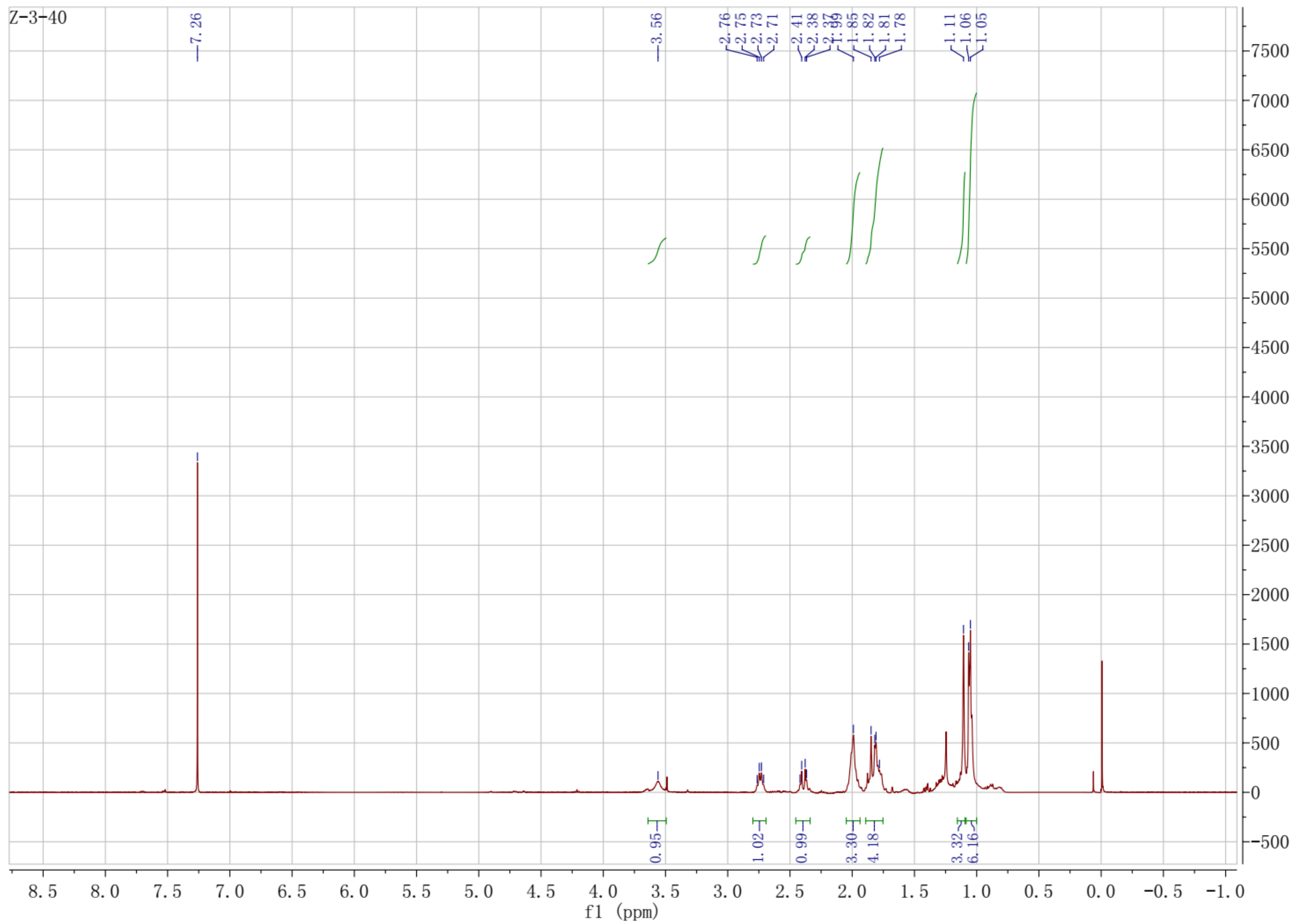

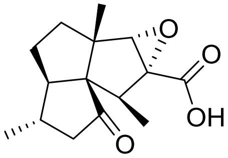

4

Figure S11. ^{13}C NMR (75 MHz, $CDCl_3$) of compound **4**

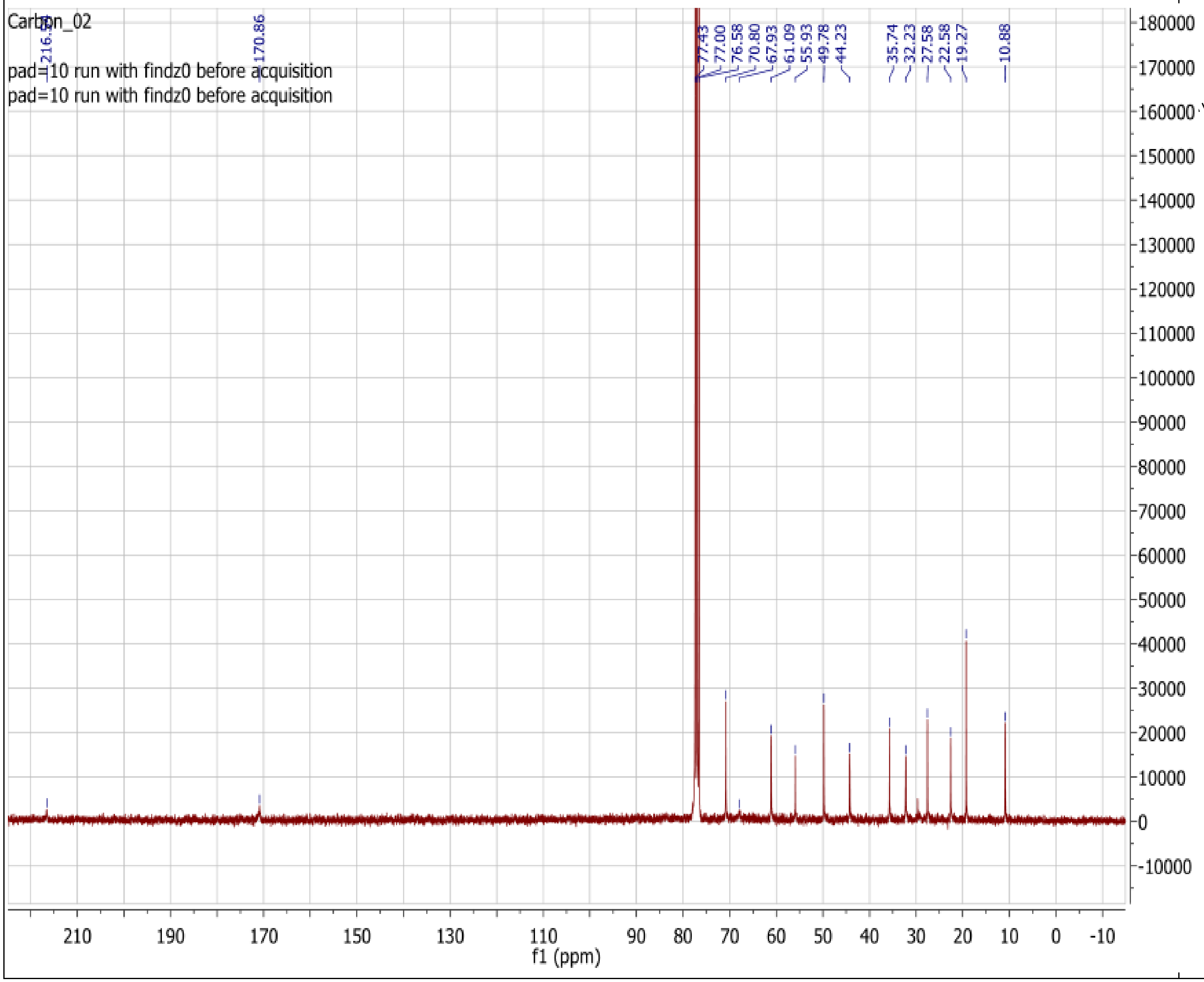

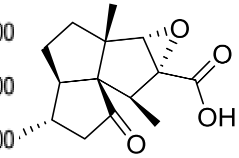

4

Figure S12. HSQC spectrum of compound **4**

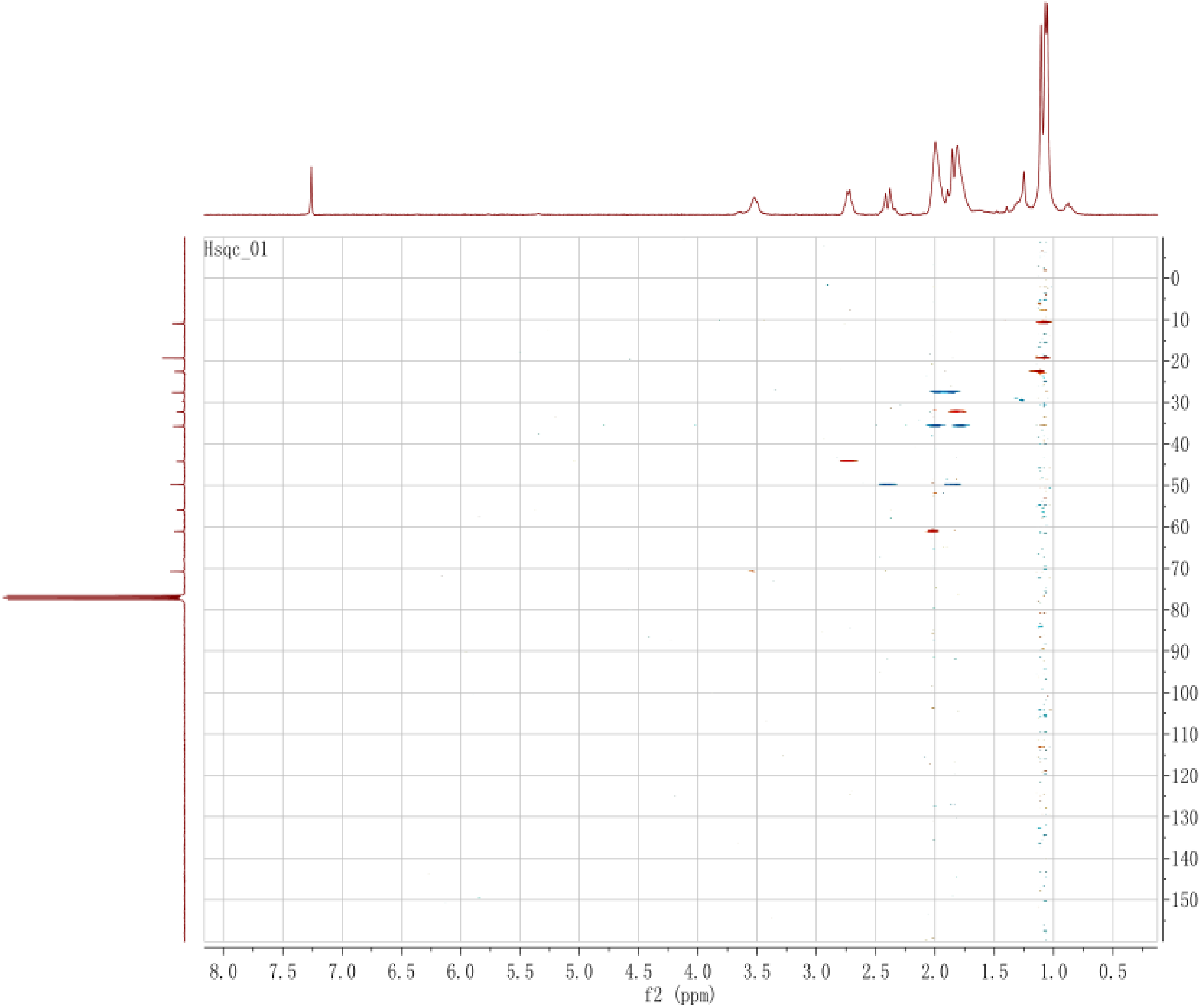

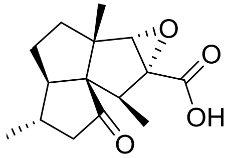

4

Figure S13. NOESY spectrum of compound **4**

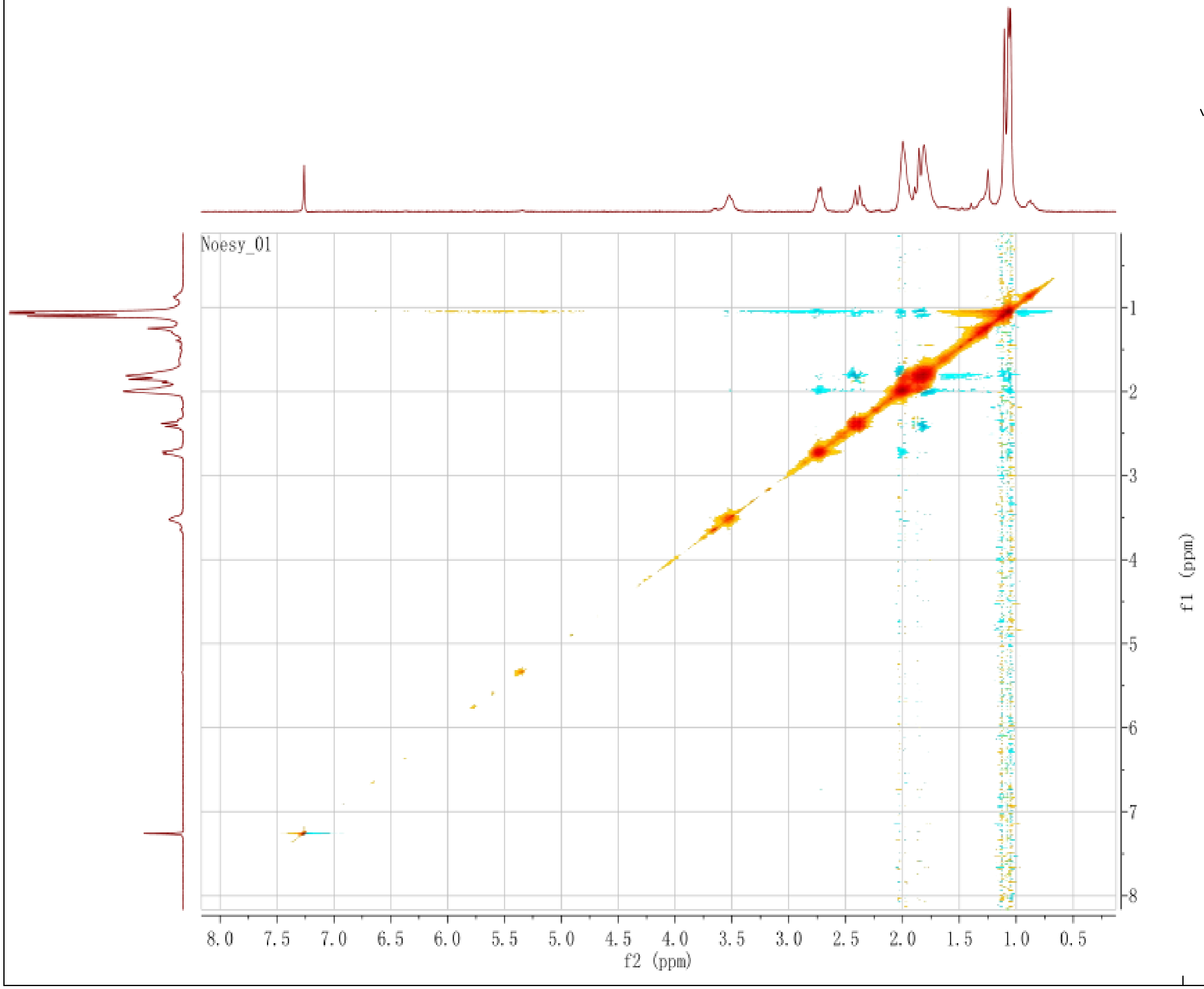

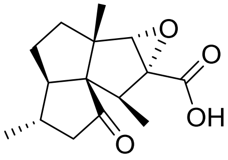

4

Figure S14. ^{1}H NMR (400 MHz, $CDCl_3$) of compound **5**

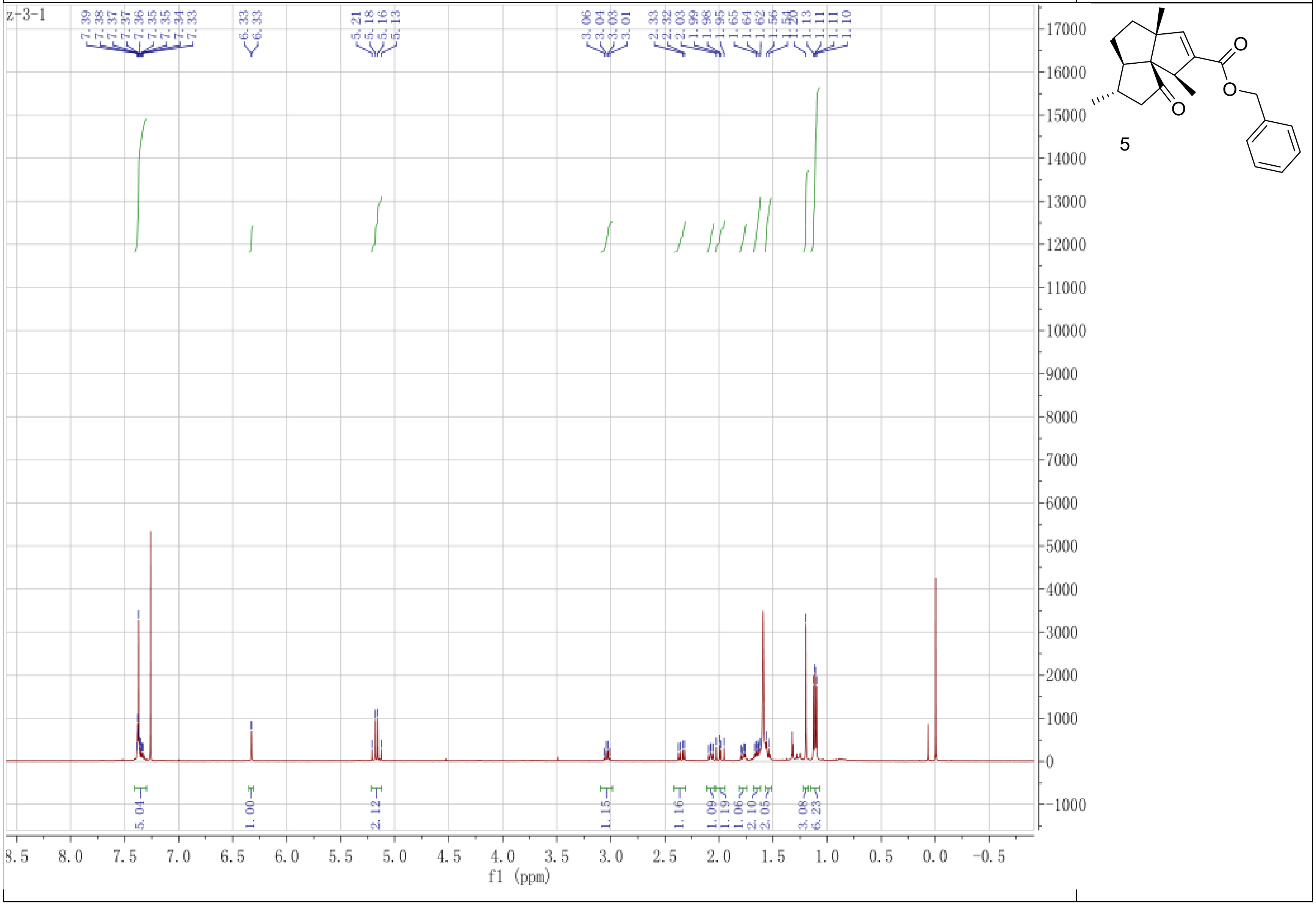

Figure S15. ^{13}C NMR (100 MHz, $CDCl_3$) of compound **5**

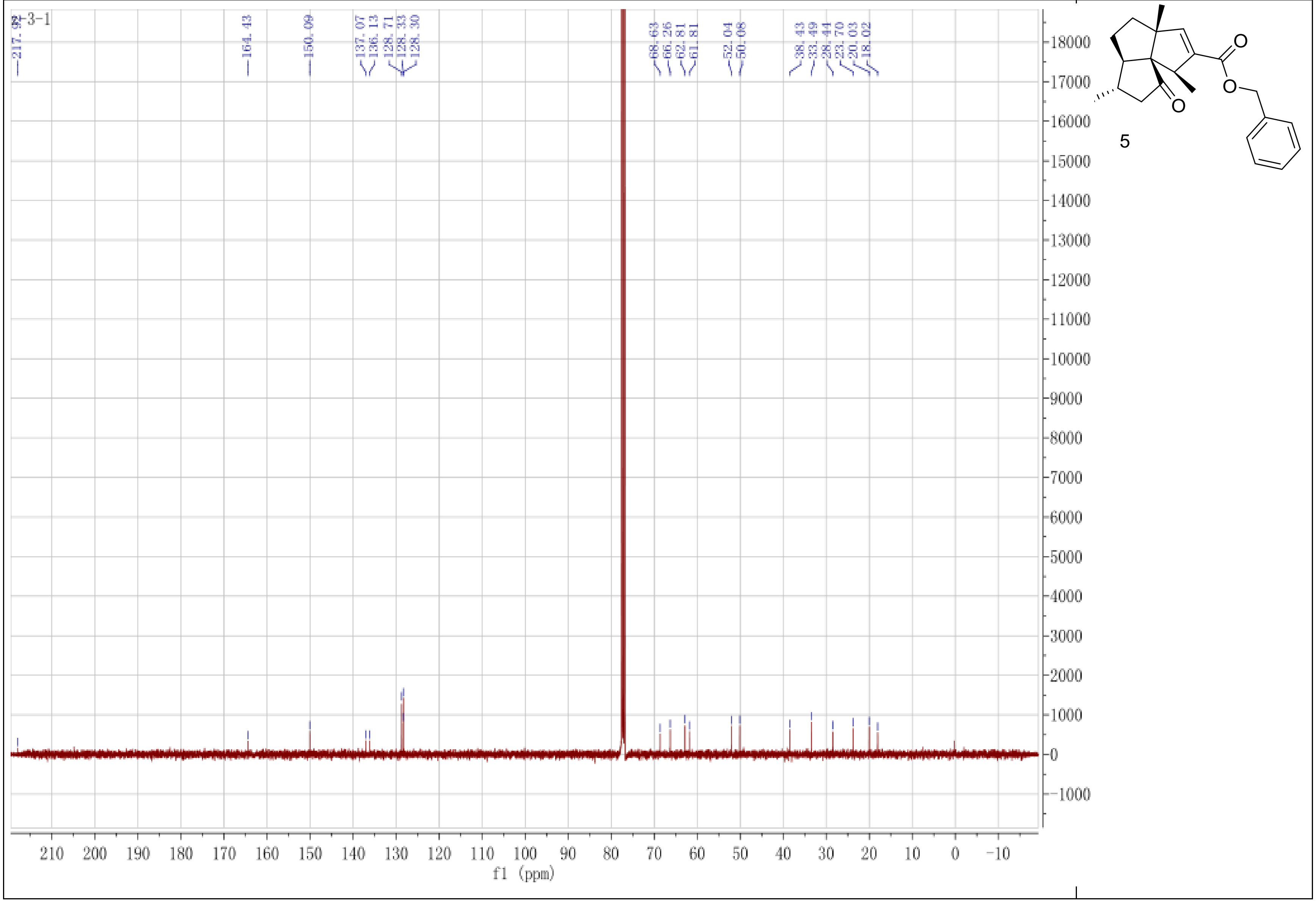

Figure S16. ^{1}H NMR (300 MHz, $CDCl_3$) of compound **6**

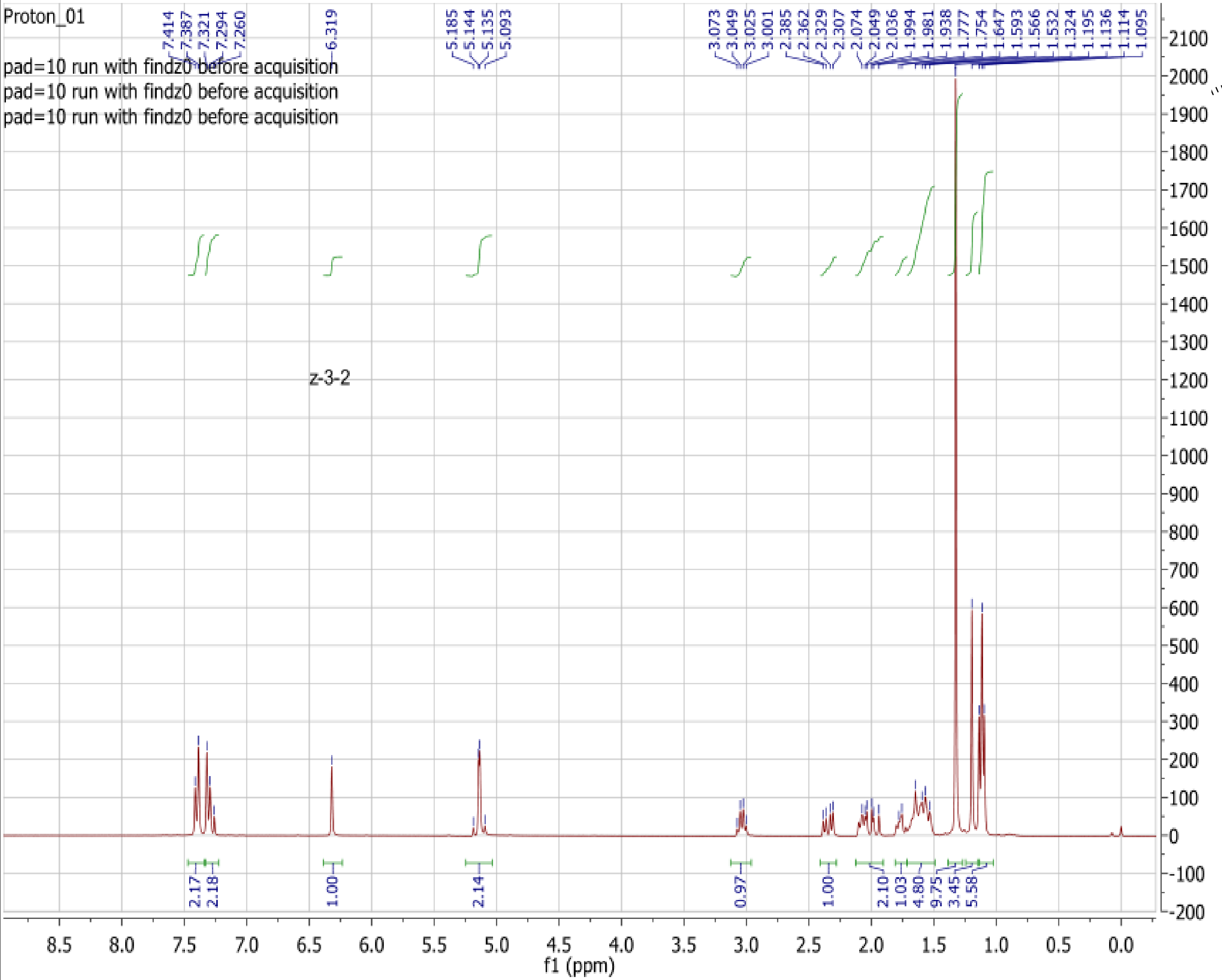

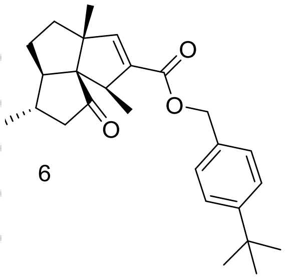

6

Figure S17. ^{13}C NMR (75 MHz, $CDCl_3$) of compound **6**

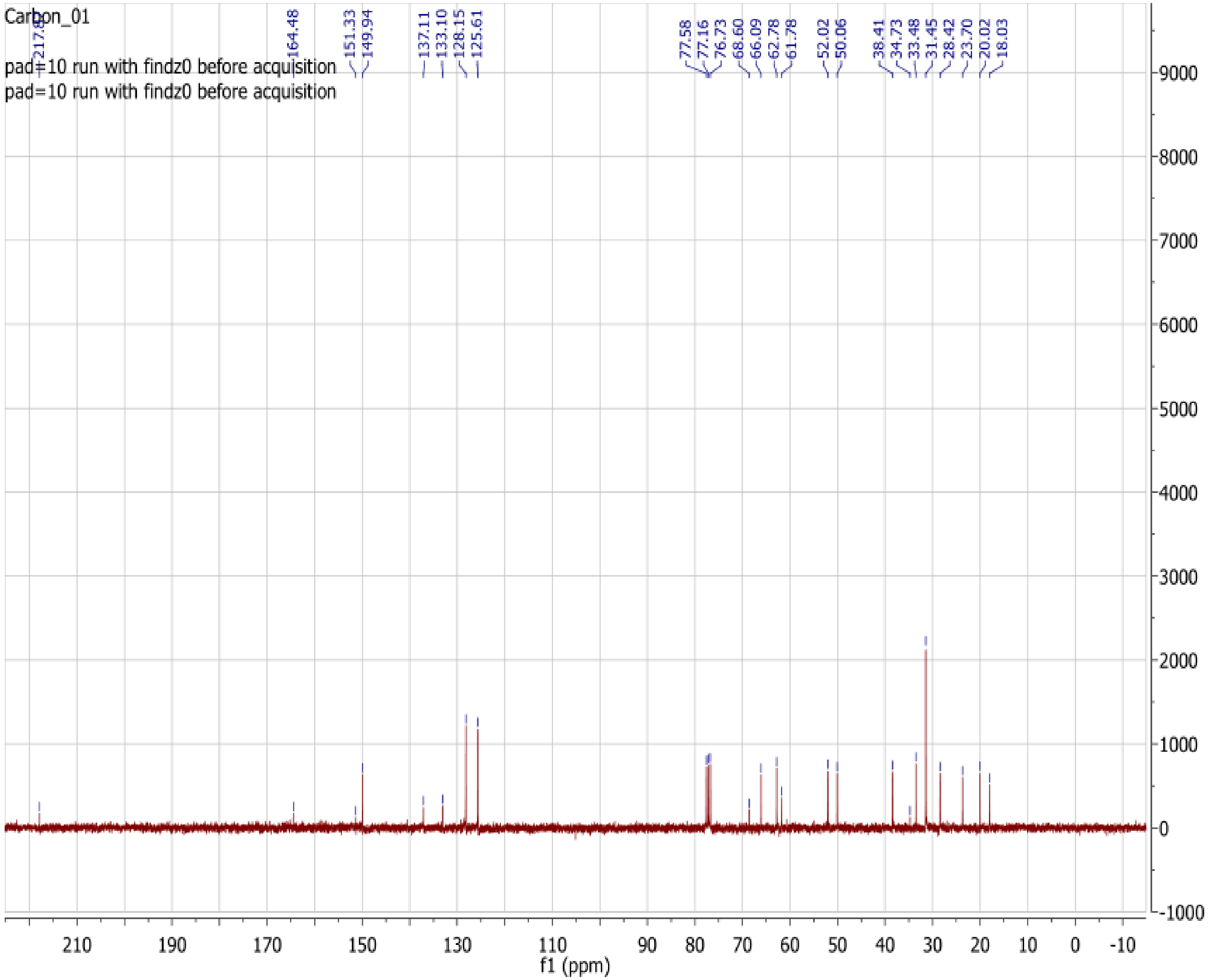

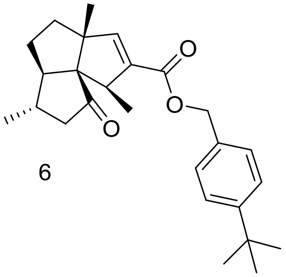

6

Figure S18. ^1H NMR (300 MHz, $CDCl_3$) of compound **7**

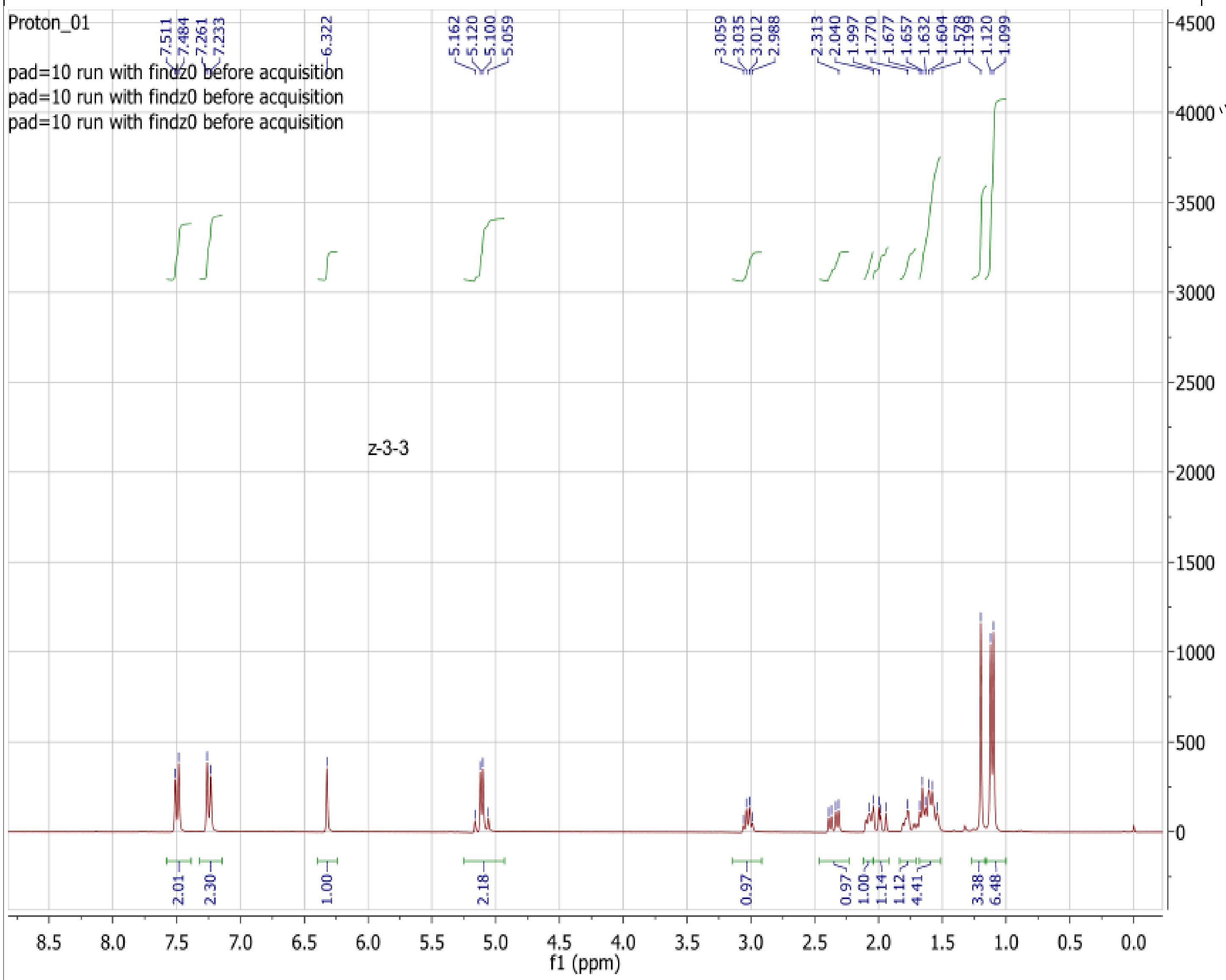

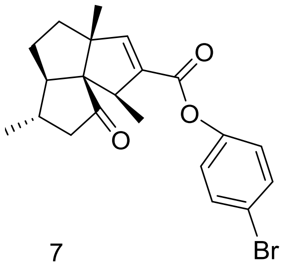

7

Figure S19. ^{13}C NMR (75 MHz, $CDCl_3$) of compound **7**

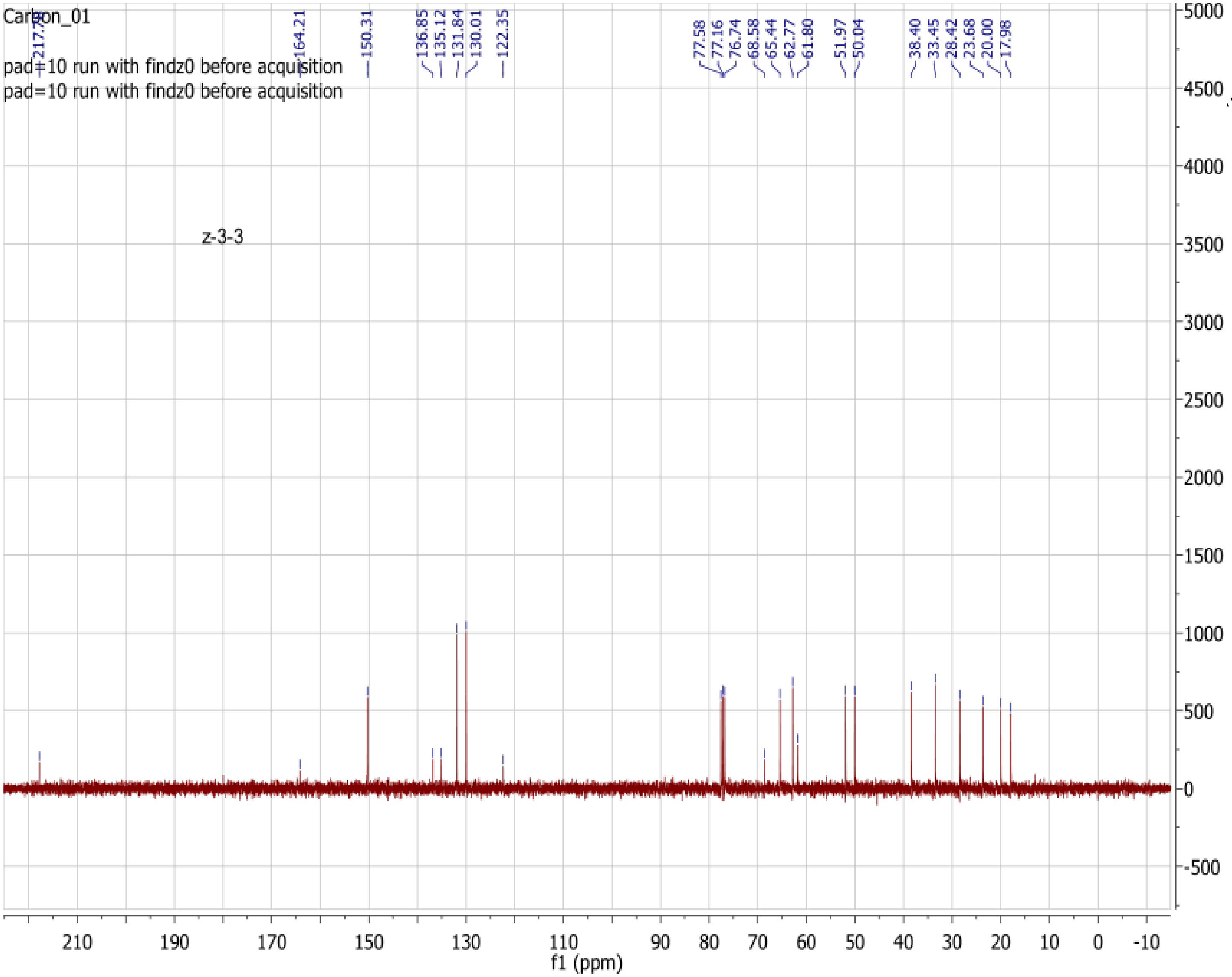

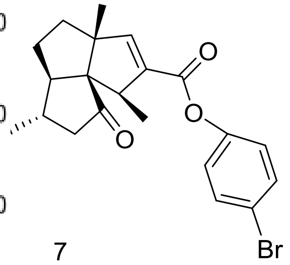

7

Figure S20. ^1H NMR (300 MHz, $CDCl_3$) of compound **8**

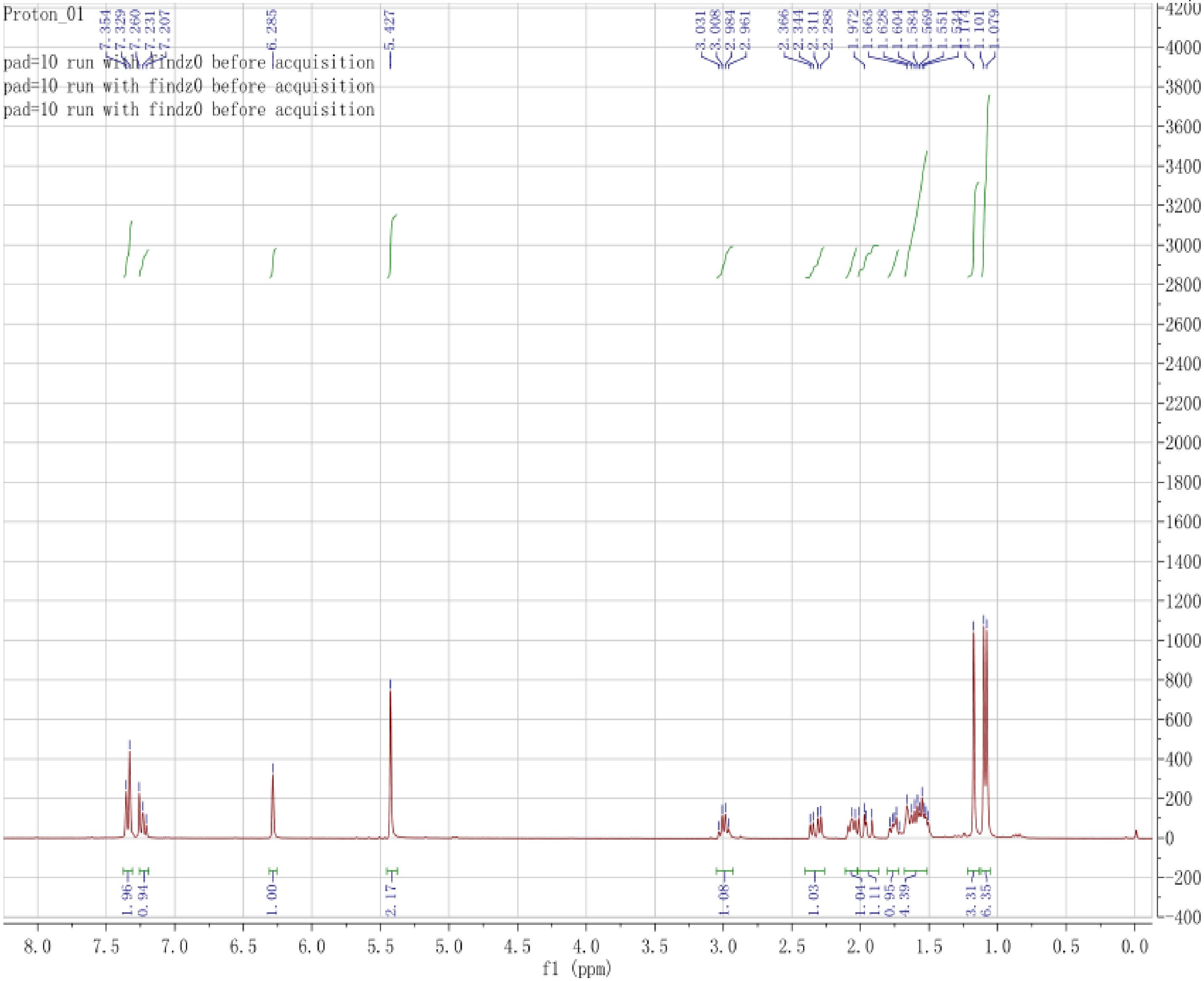

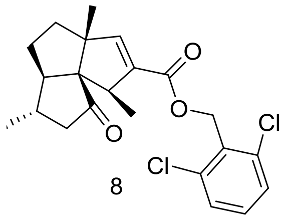

8

Figure S21. ^{13}C NMR (75 MHz, $CDCl_3$) of compound **8**

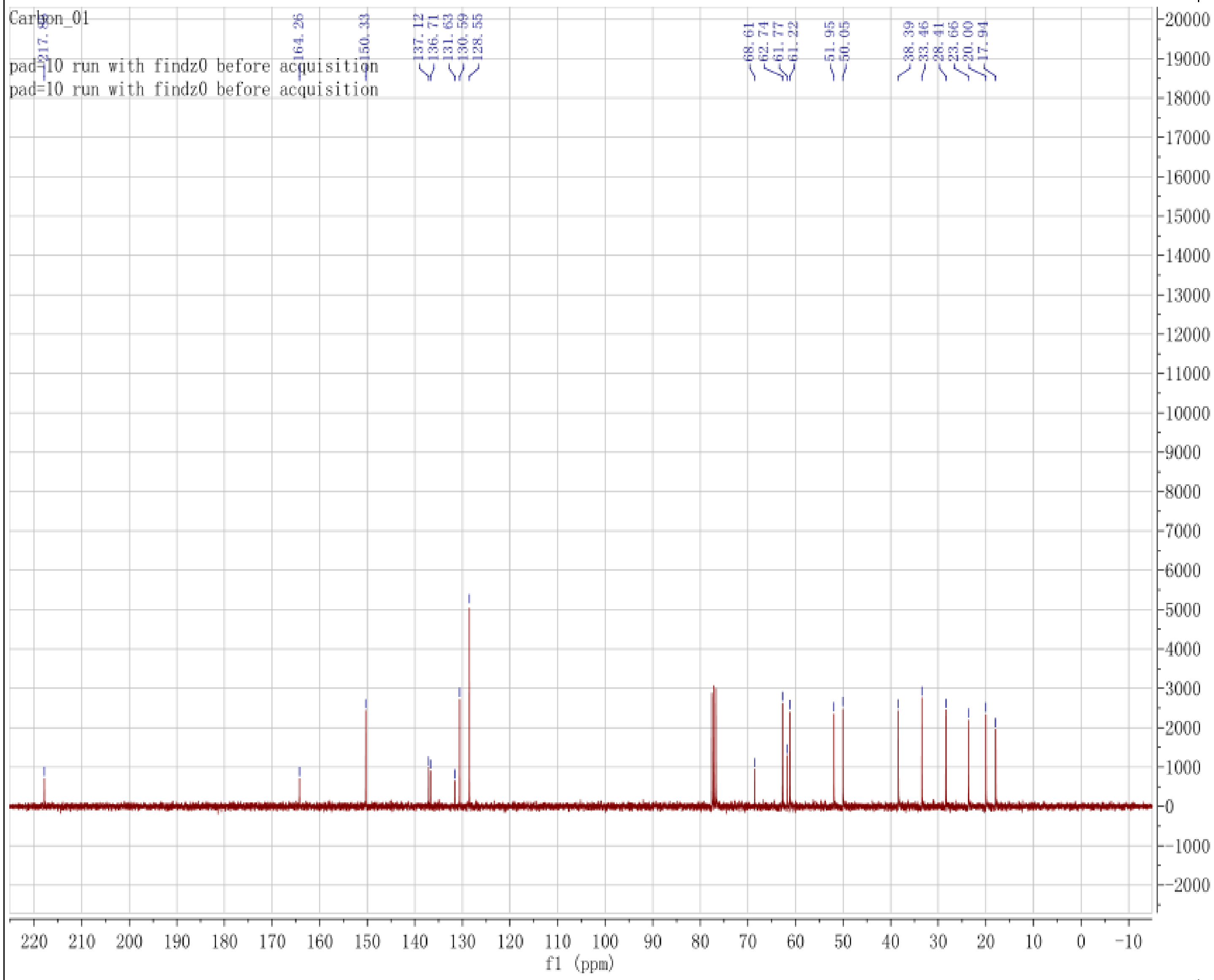

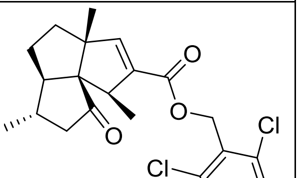

8

Figure S22. ^{1}H NMR (300 MHz, $CDCl_3$) of compound **9**

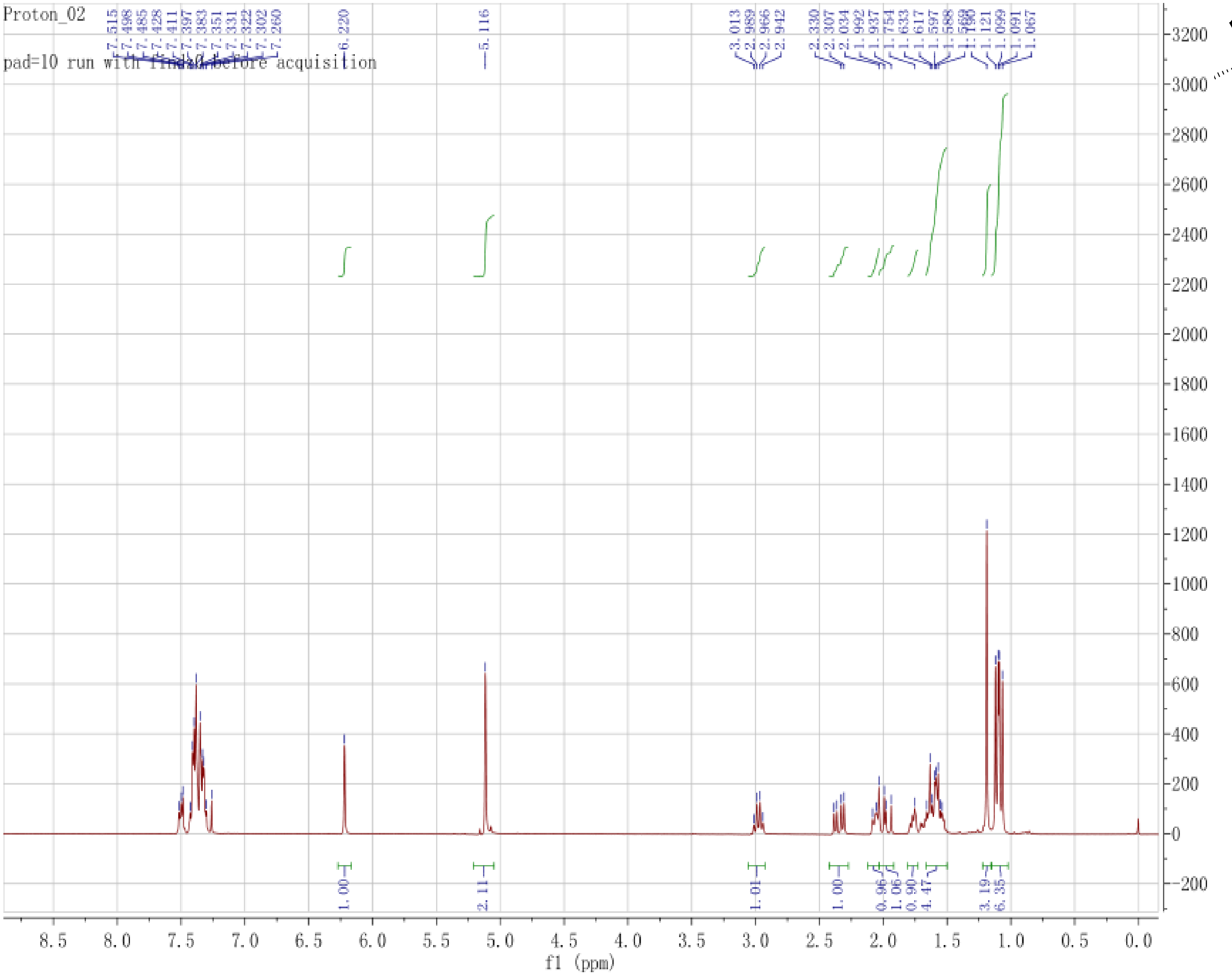

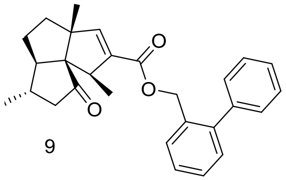

9

Figure S23. ^{13}C NMR (75 MHz, $CDCl_3$) of compound **9**

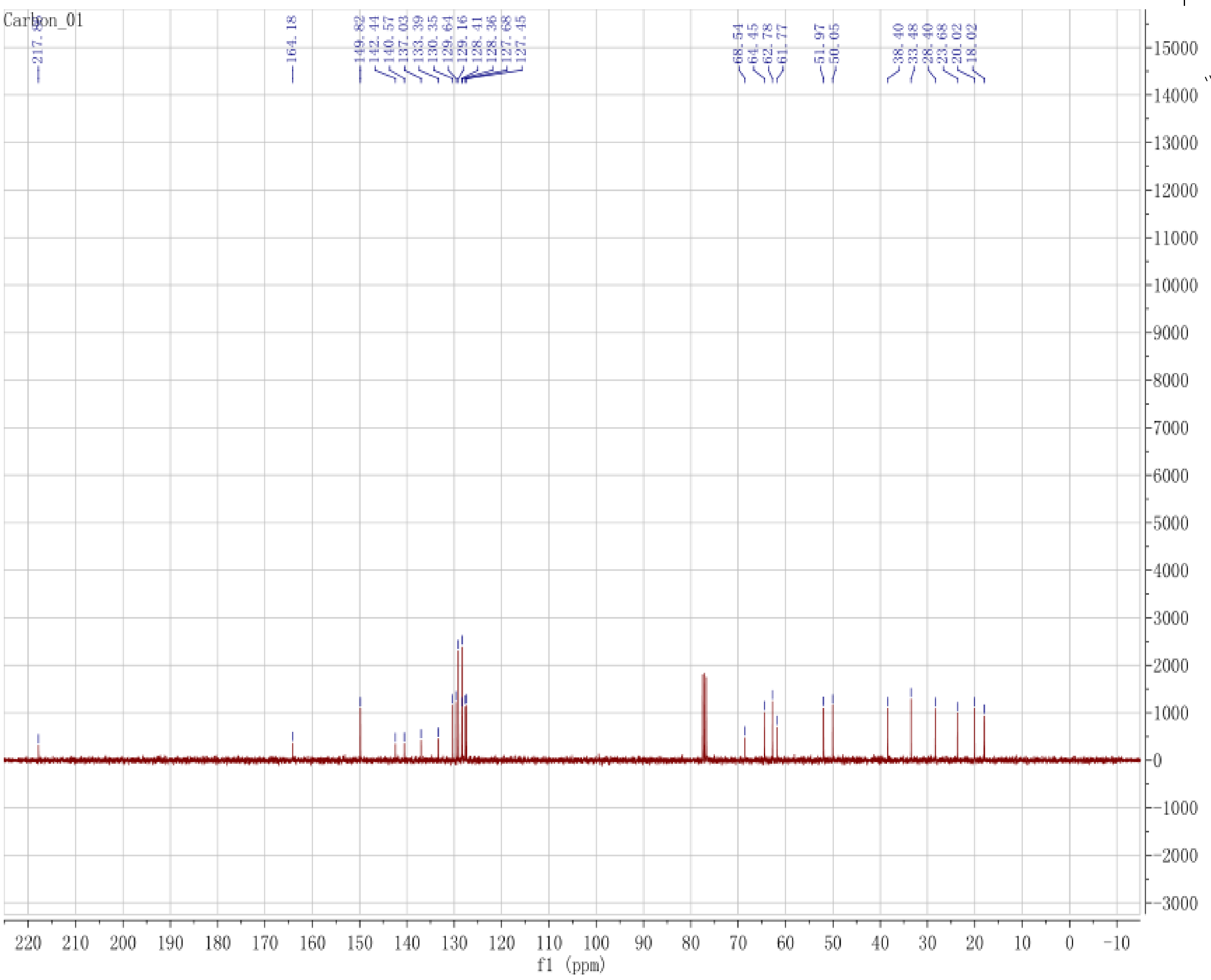

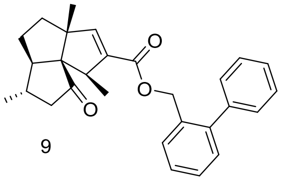

9

Figure S24. ^{1}H NMR (300 MHz, $CDCl_3$) of compound **10**

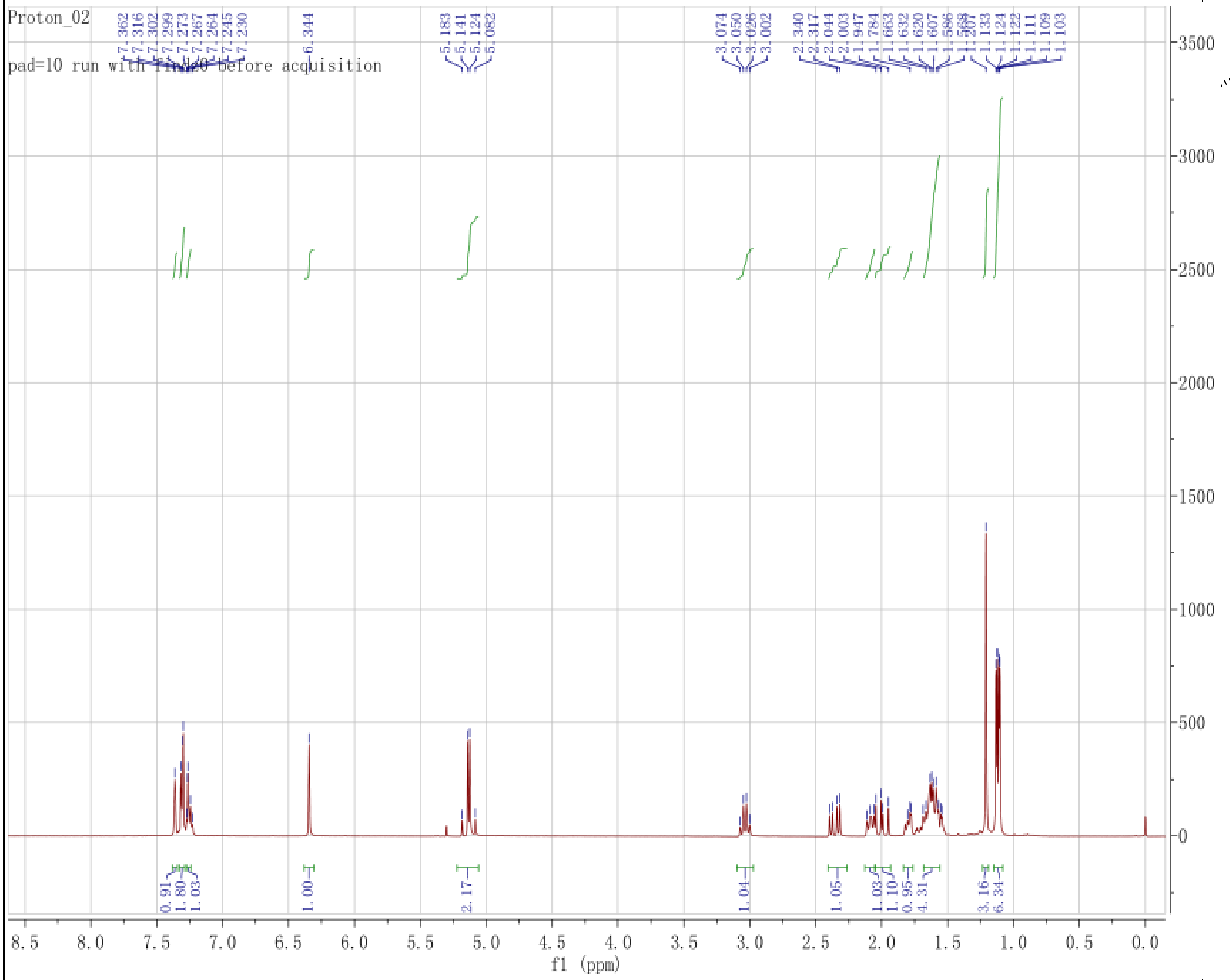

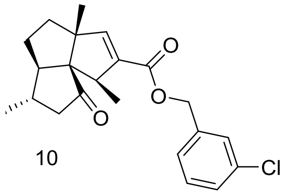

10

Figure S25. ^{13}C NMR (75 MHz, $CDCl_3$) of compound **10**

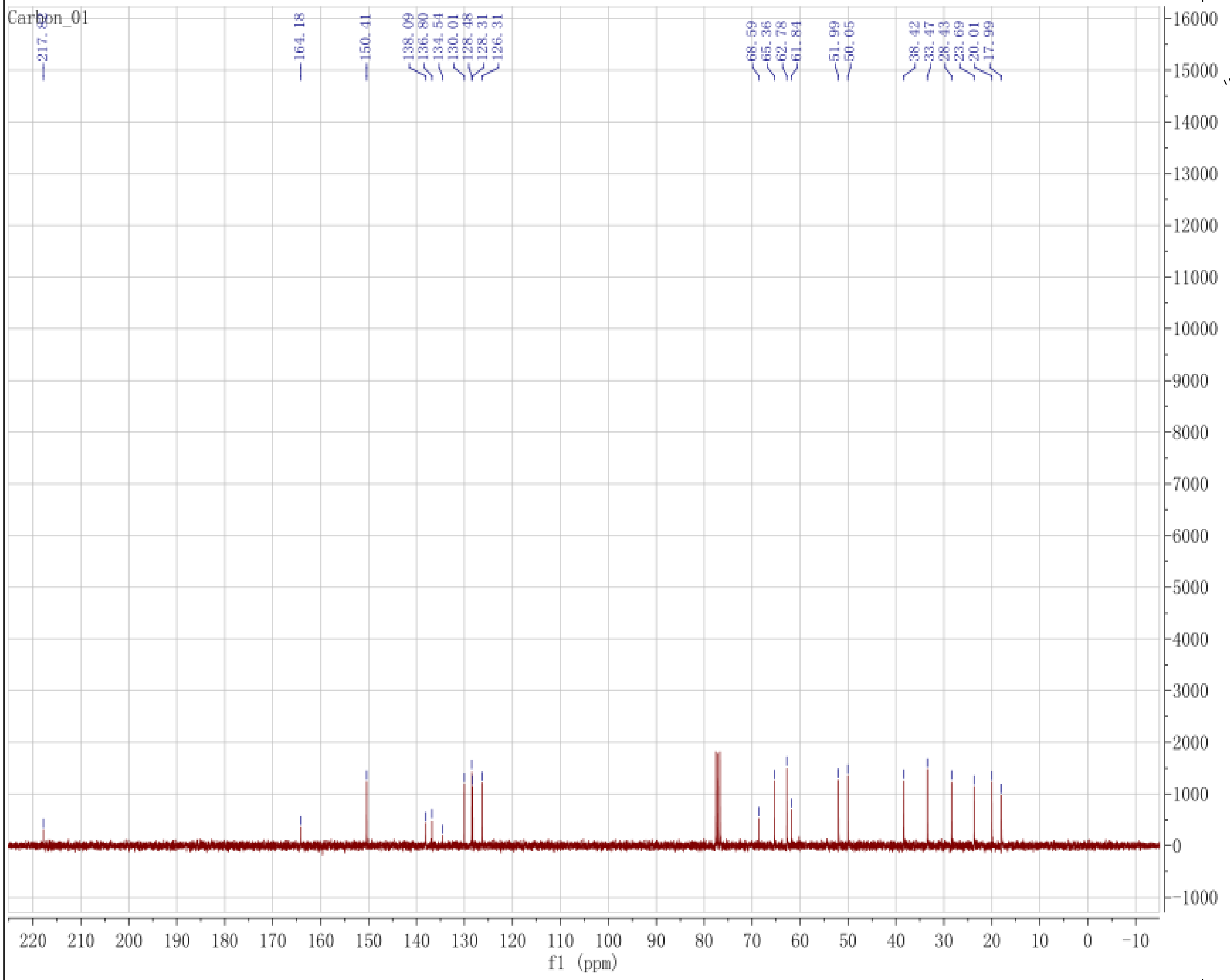

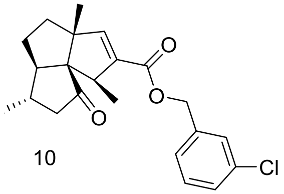

10

Figure S26. ^{1}H NMR (300 MHz, $CDCl_3$) of compound **11**

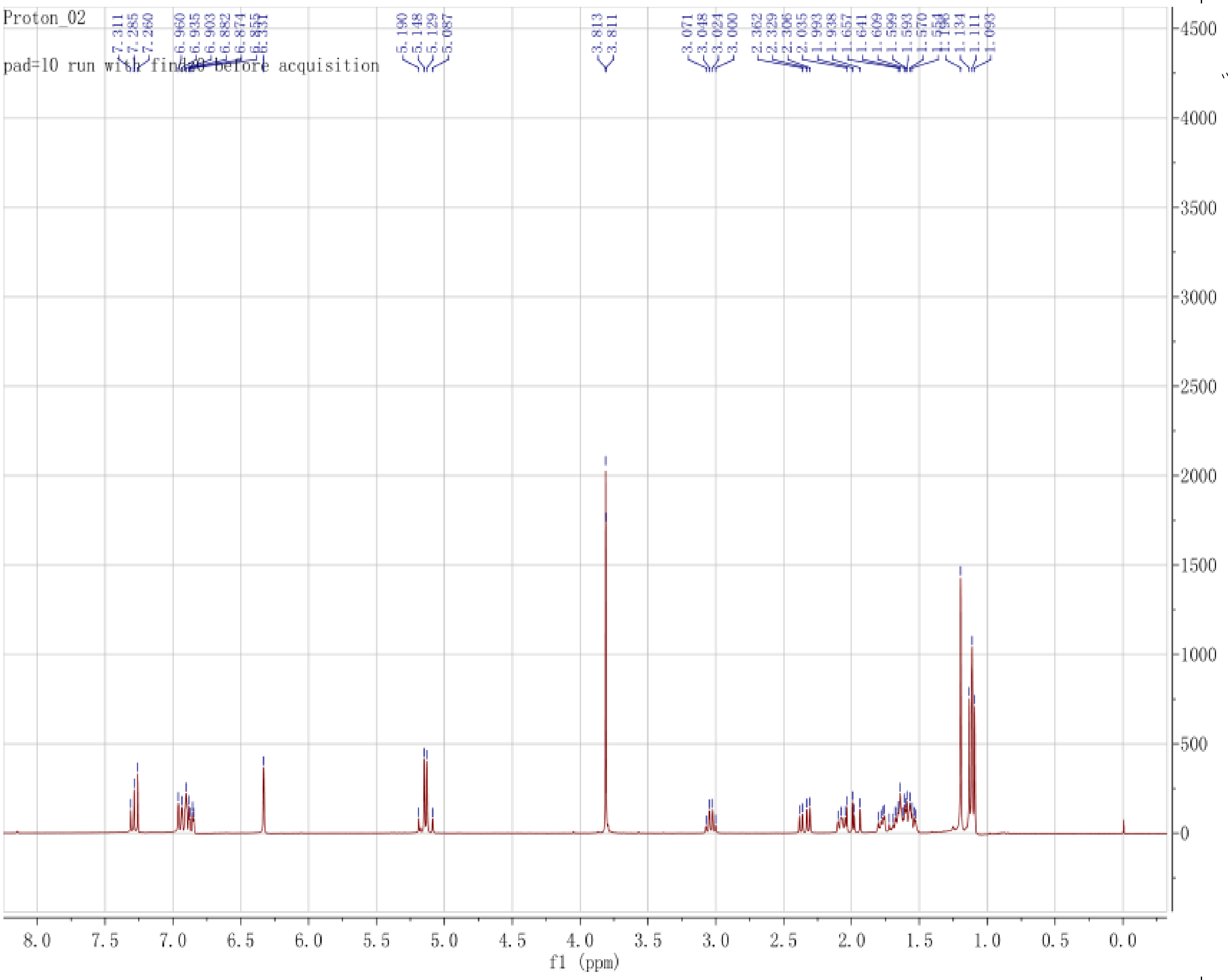

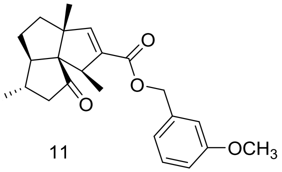

11

Figure S27. ^{13}C NMR (75 MHz, $CDCl_3$) of compound **11**

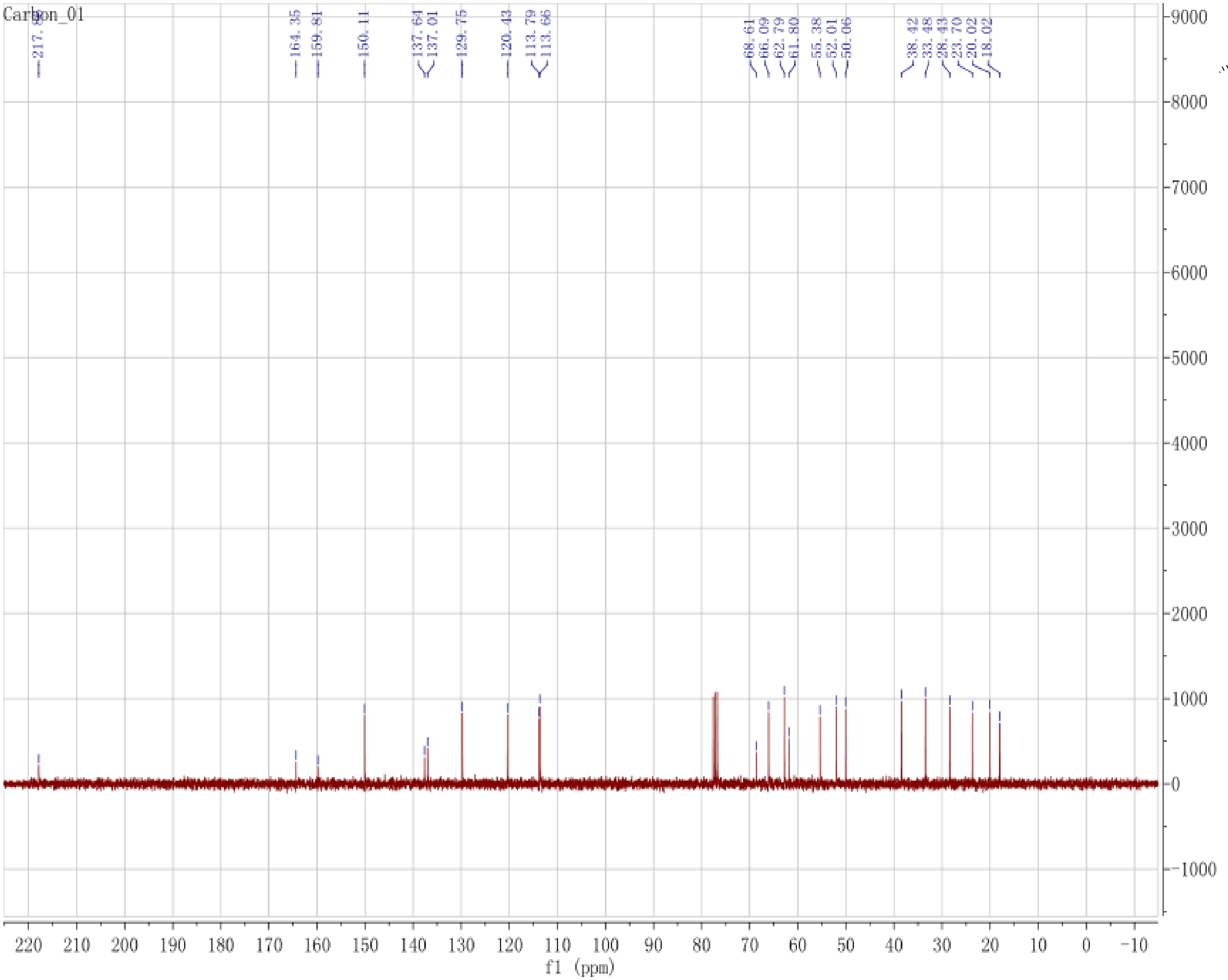

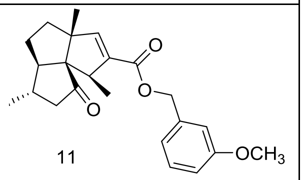

11

Figure S28. ^{1}H NMR (300 MHz, $CDCl_3$) of compound **12**

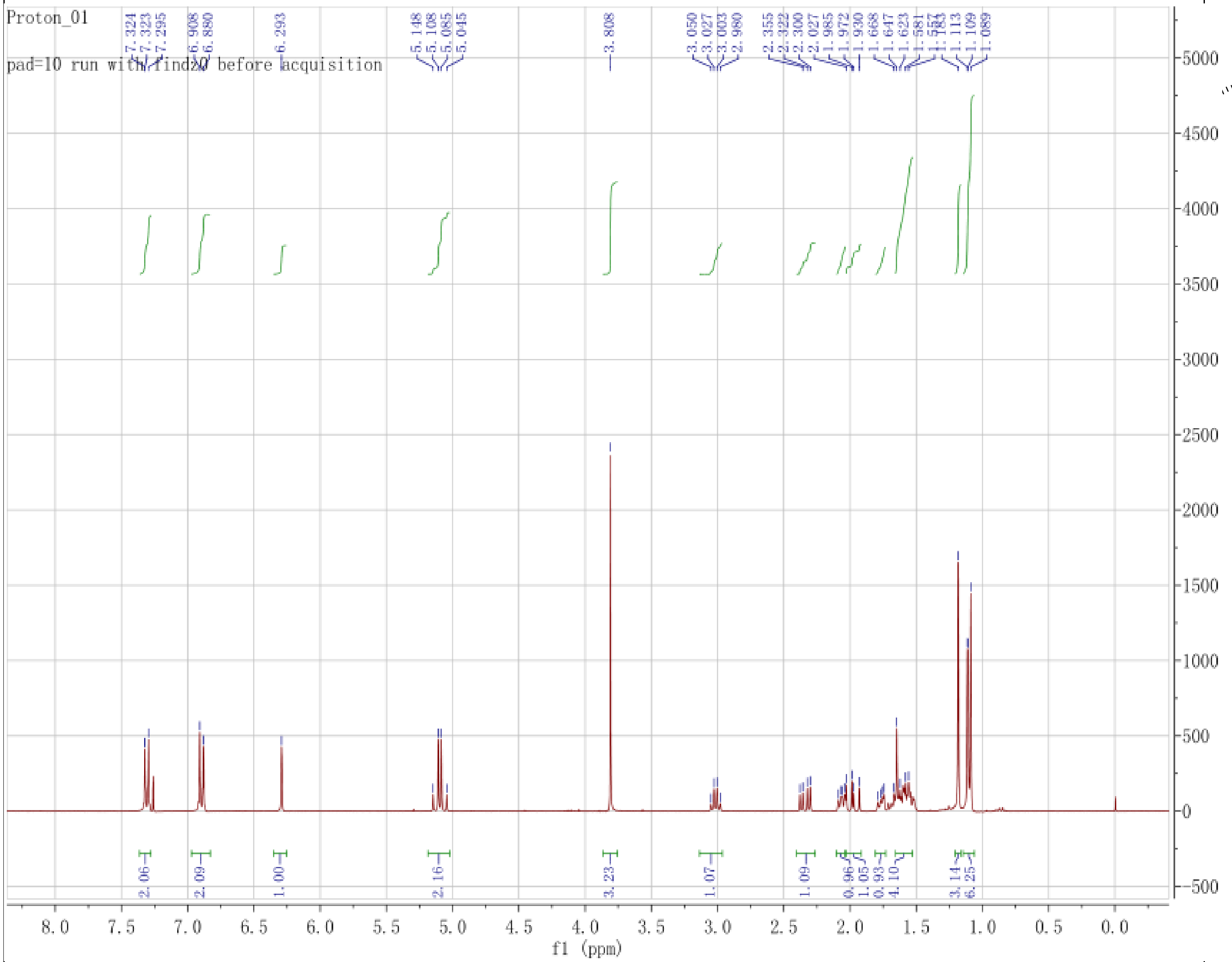

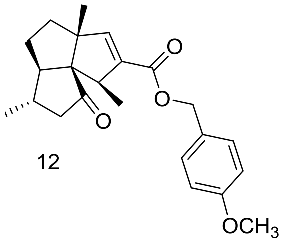

12

Figure S29. ^{13}C NMR (75 MHz, $CDCl_3$) of compound **12**

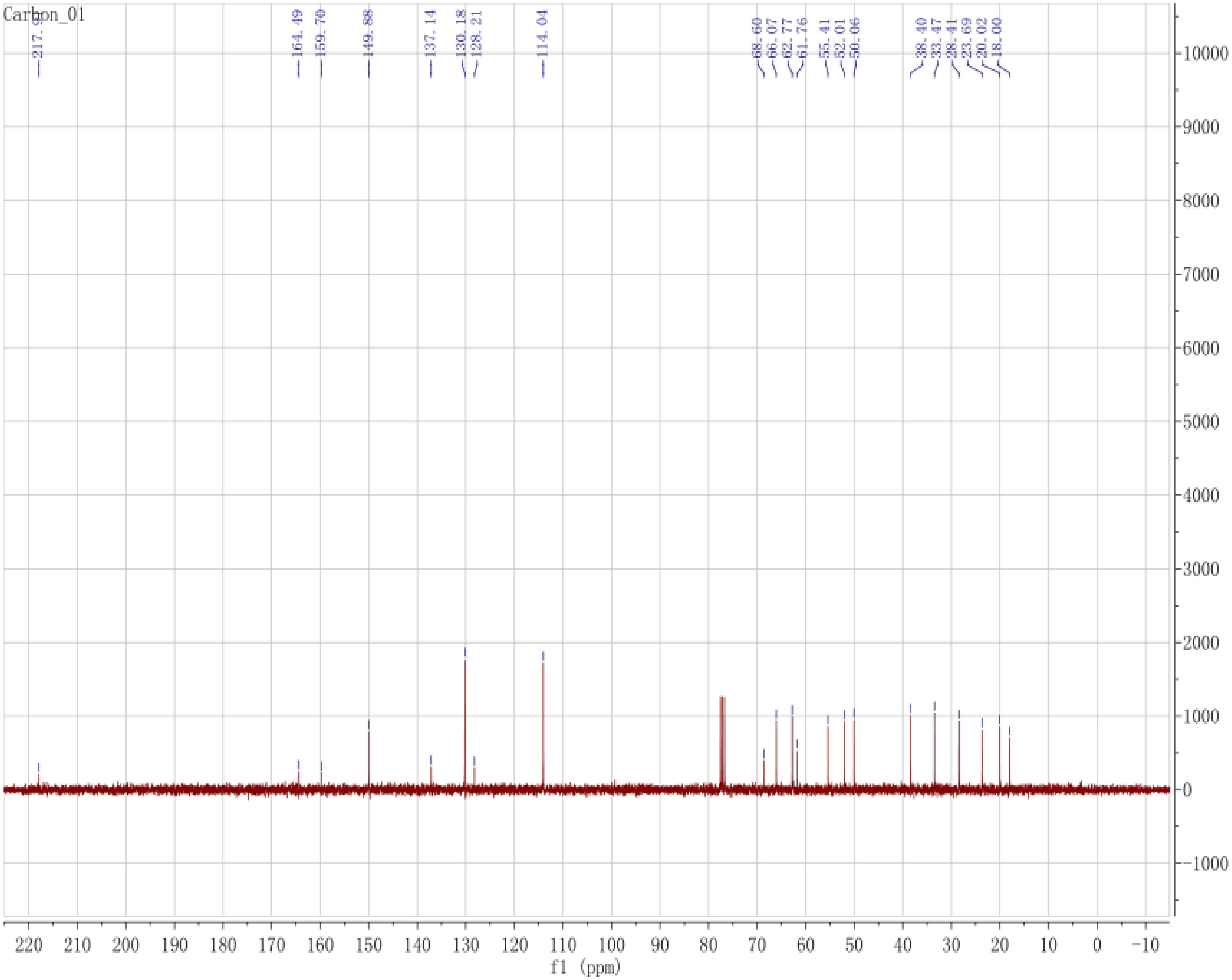

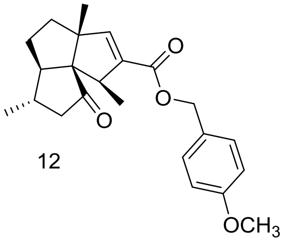

12

Figure S30. ^{1}H NMR (300 MHz, $CDCl_3$) of compound **13**

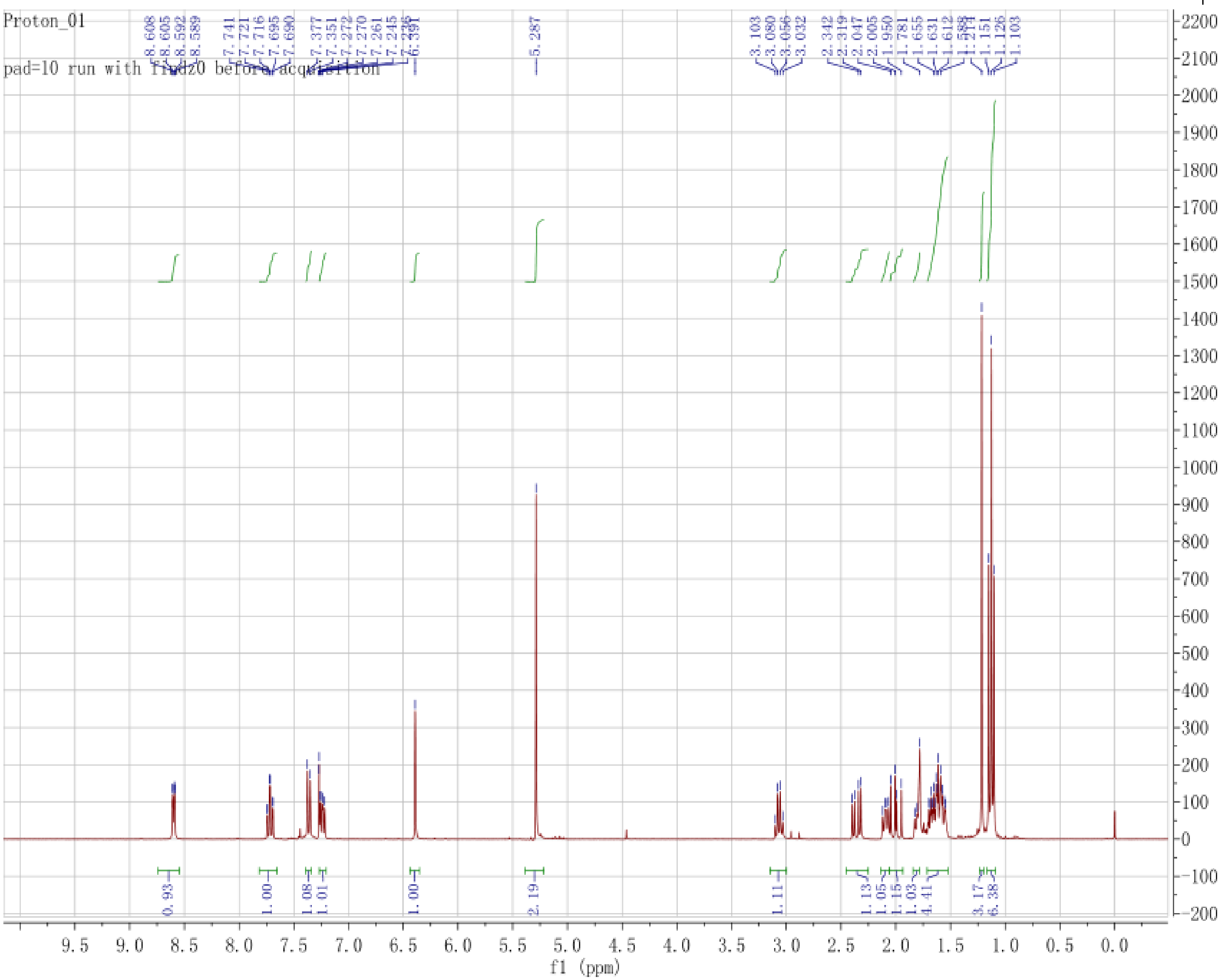

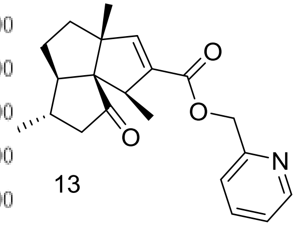

13

Figure S31. ^{13}C NMR (75 MHz, $CDCl_3$) of compound **13**

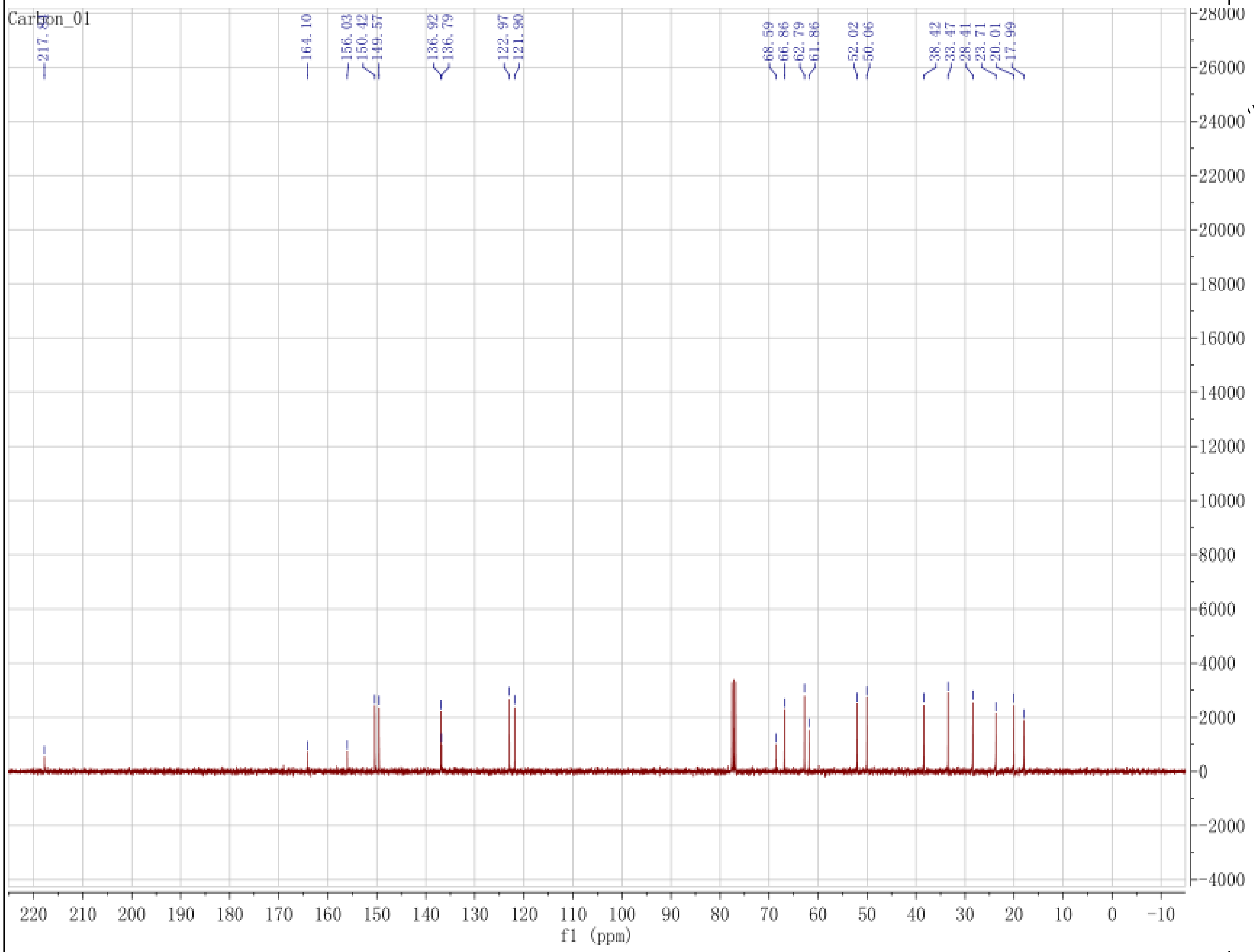

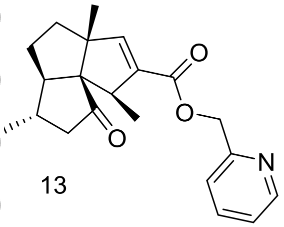

13

Figure S32. ^1H NMR (300 MHz, $CDCl_3$) of compound **14**

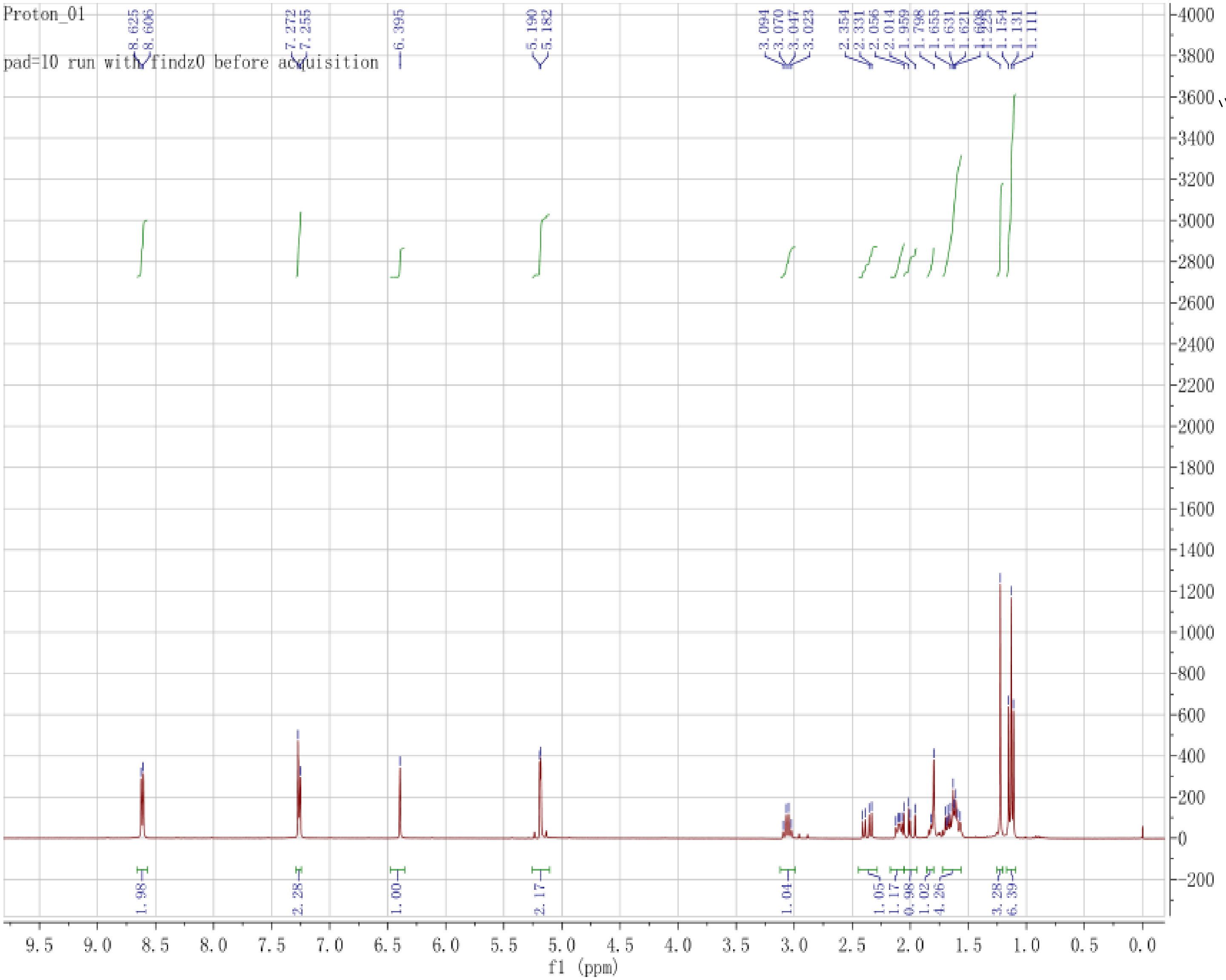

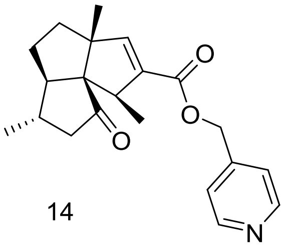

14

Figure S33. ^{13}C NMR (75 MHz, $CDCl_3$) of compound **14**

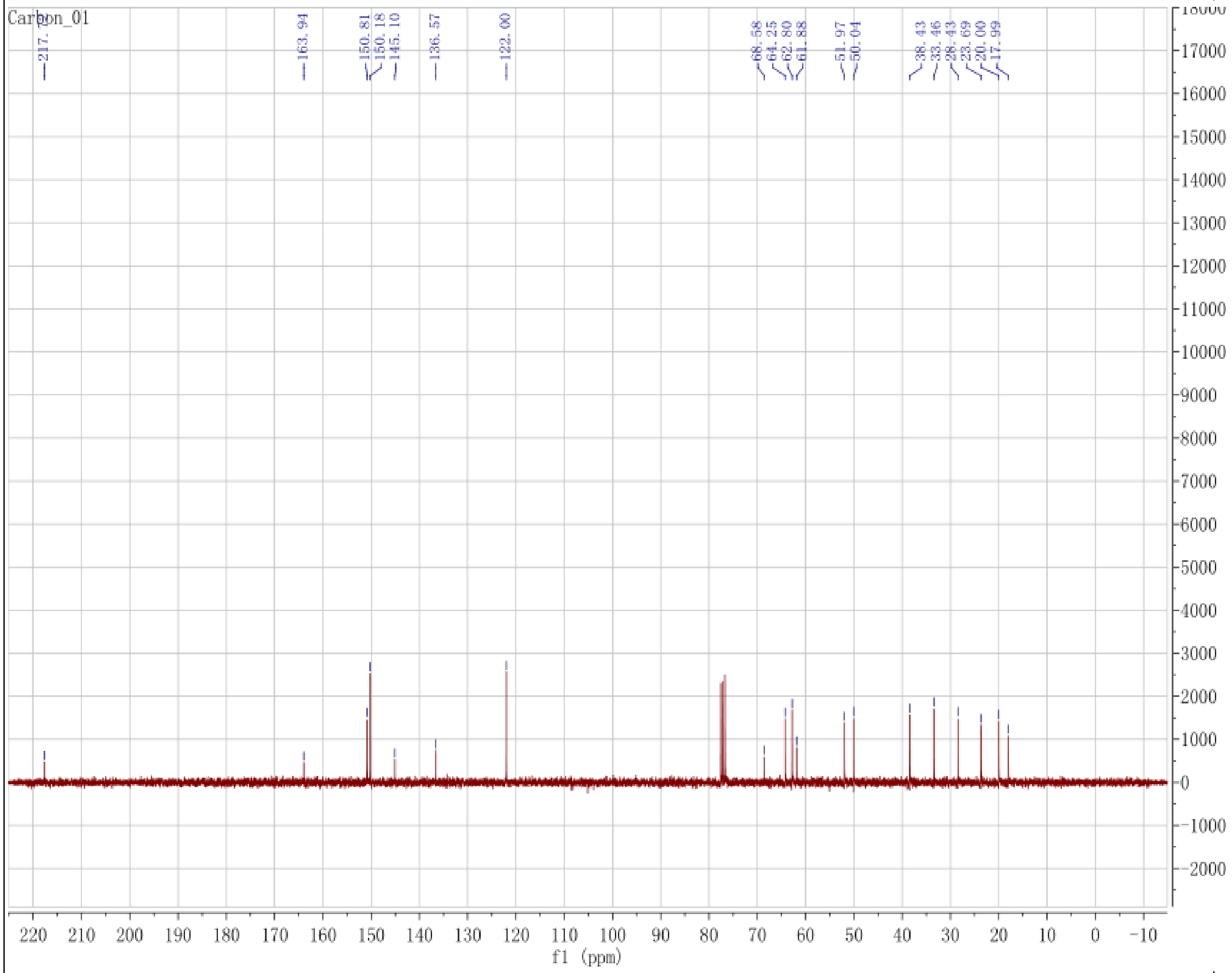

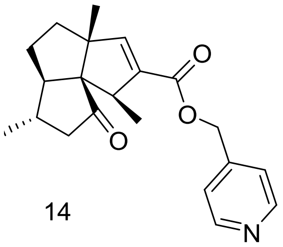

14

Figure S34. ^{1}H NMR (300 MHz, $CDCl_3$) of compound **15**

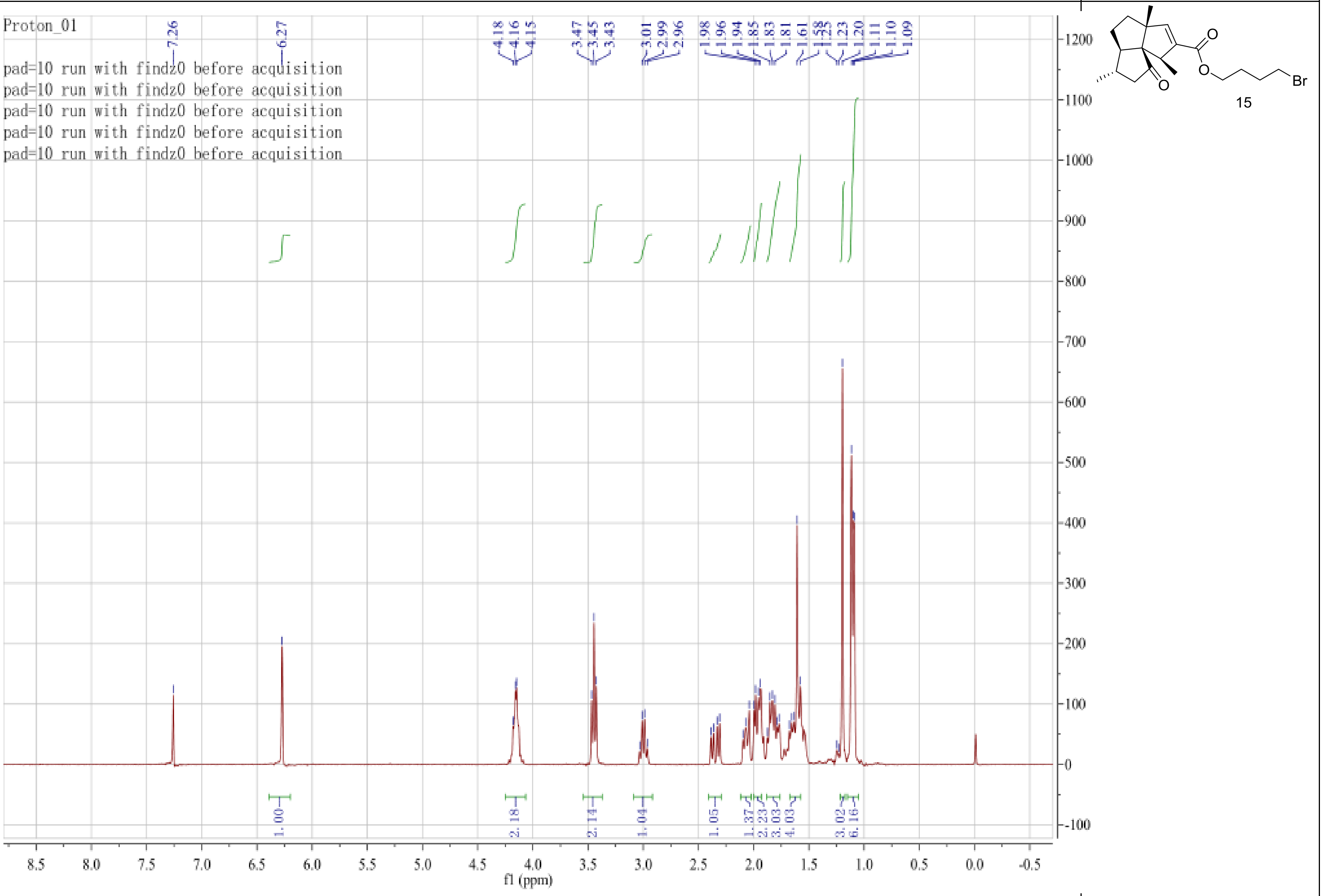

Figure S35. ^{13}C NMR (75 MHz, $CDCl_3$) of compound **15**

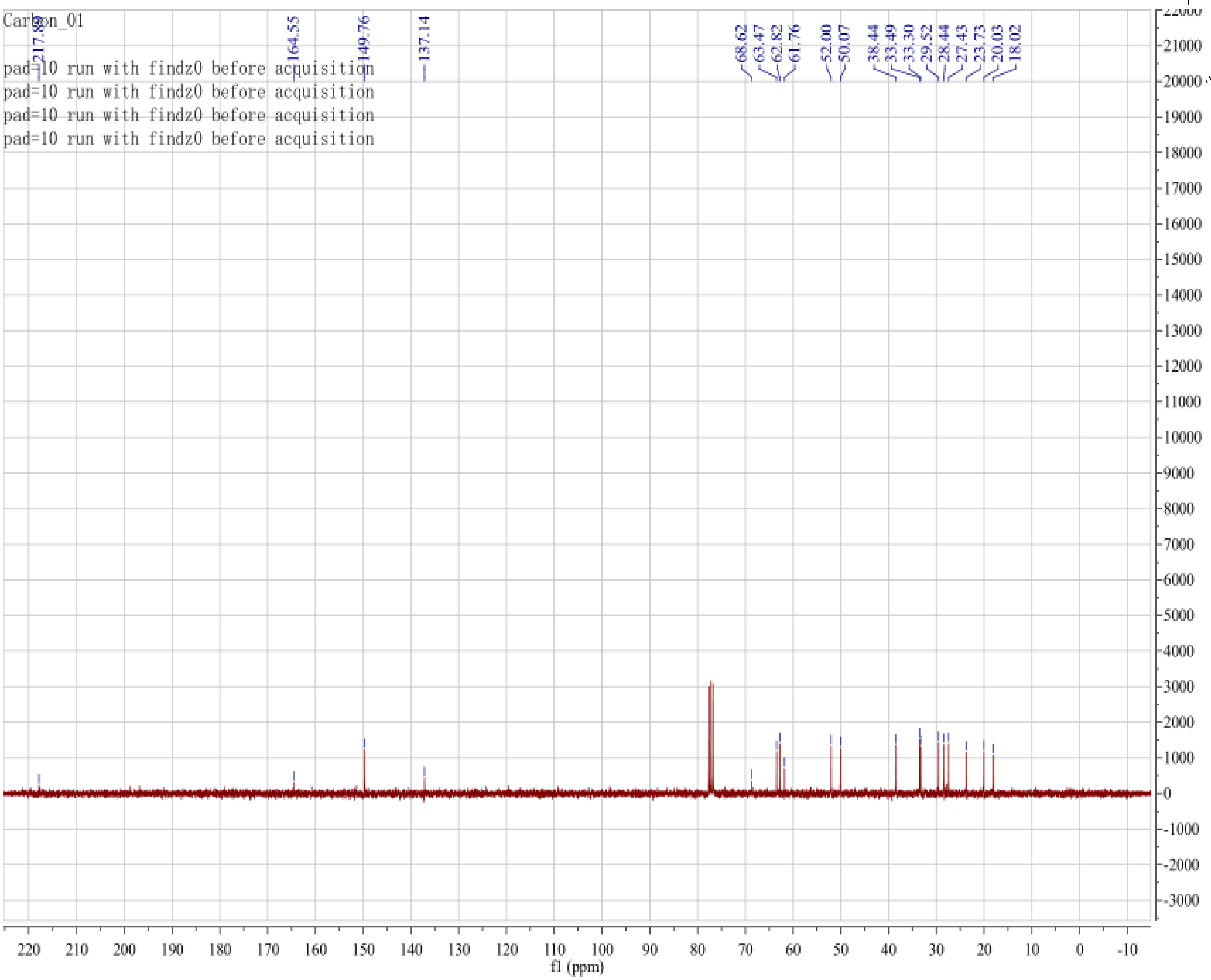

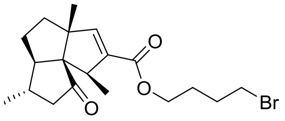

15

Figure S36. ^{1}H NMR (400 MHz, $CDCl_3$) of compound **16**

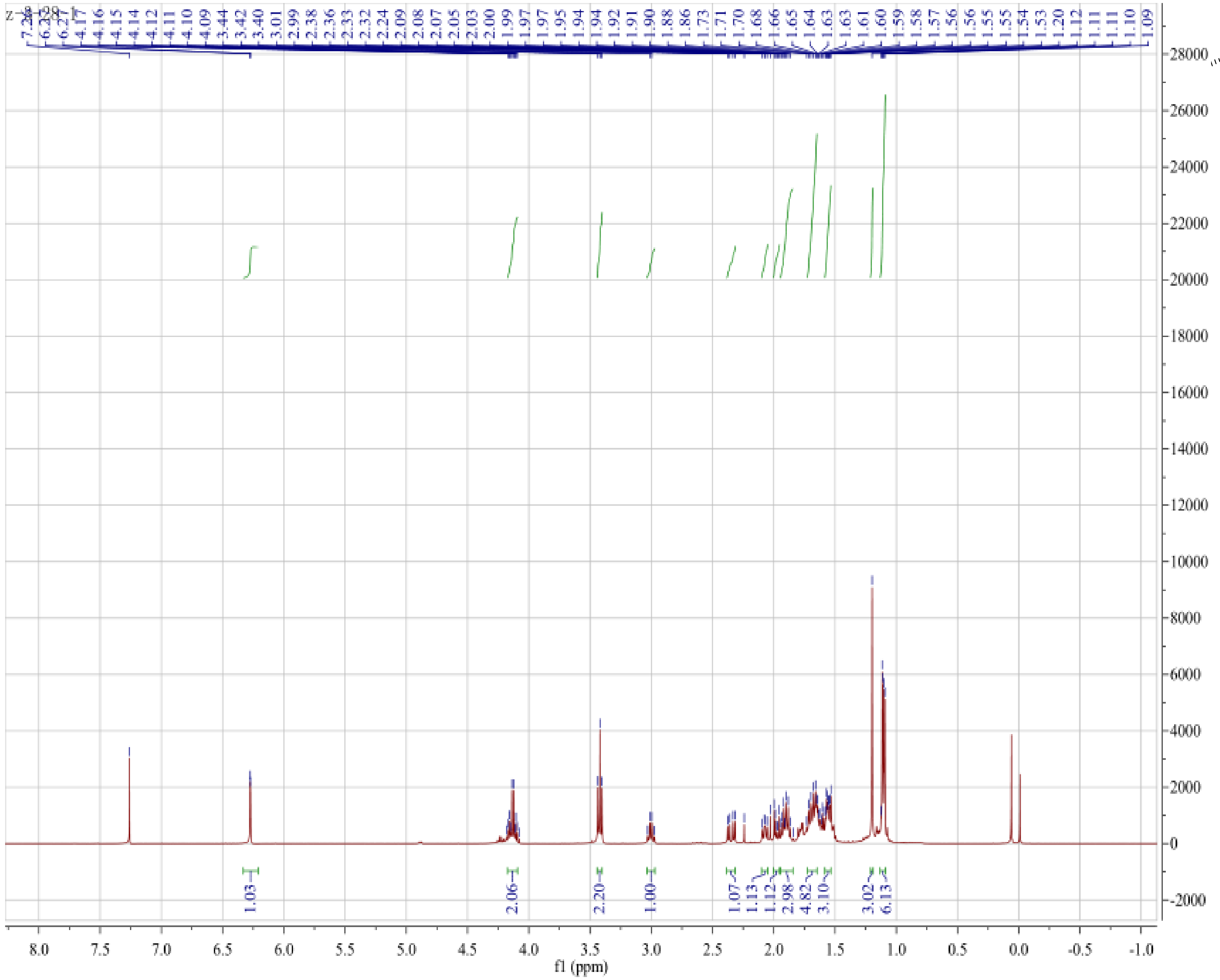

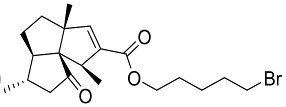

16

Figure S37. ^{13}C NMR (100 MHz, $CDCl_3$) of compound **16**

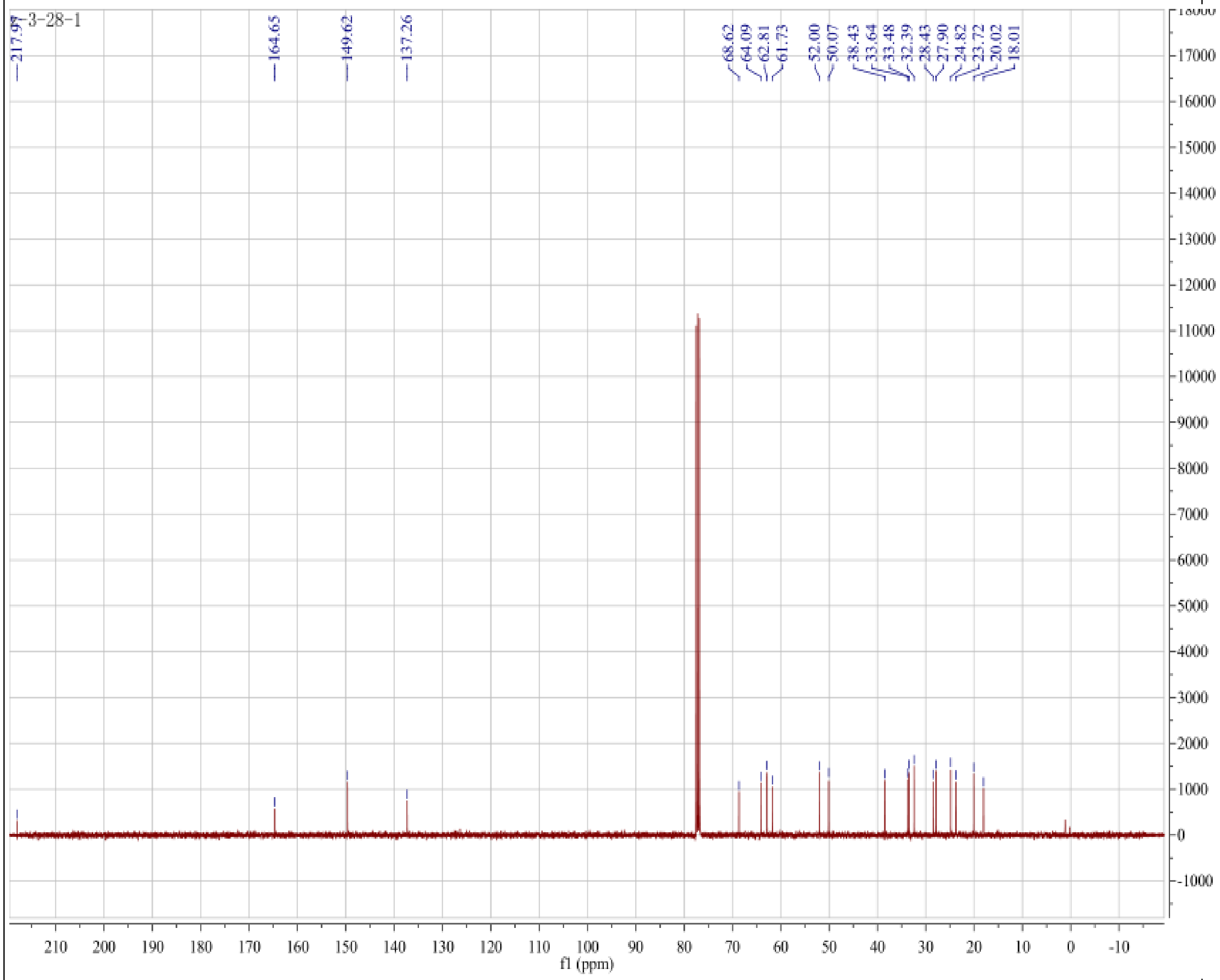

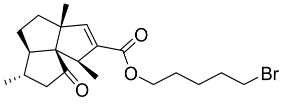

16

Figure S38. ^{1}H NMR (400 MHz, $CDCl_3$) of compound **17**

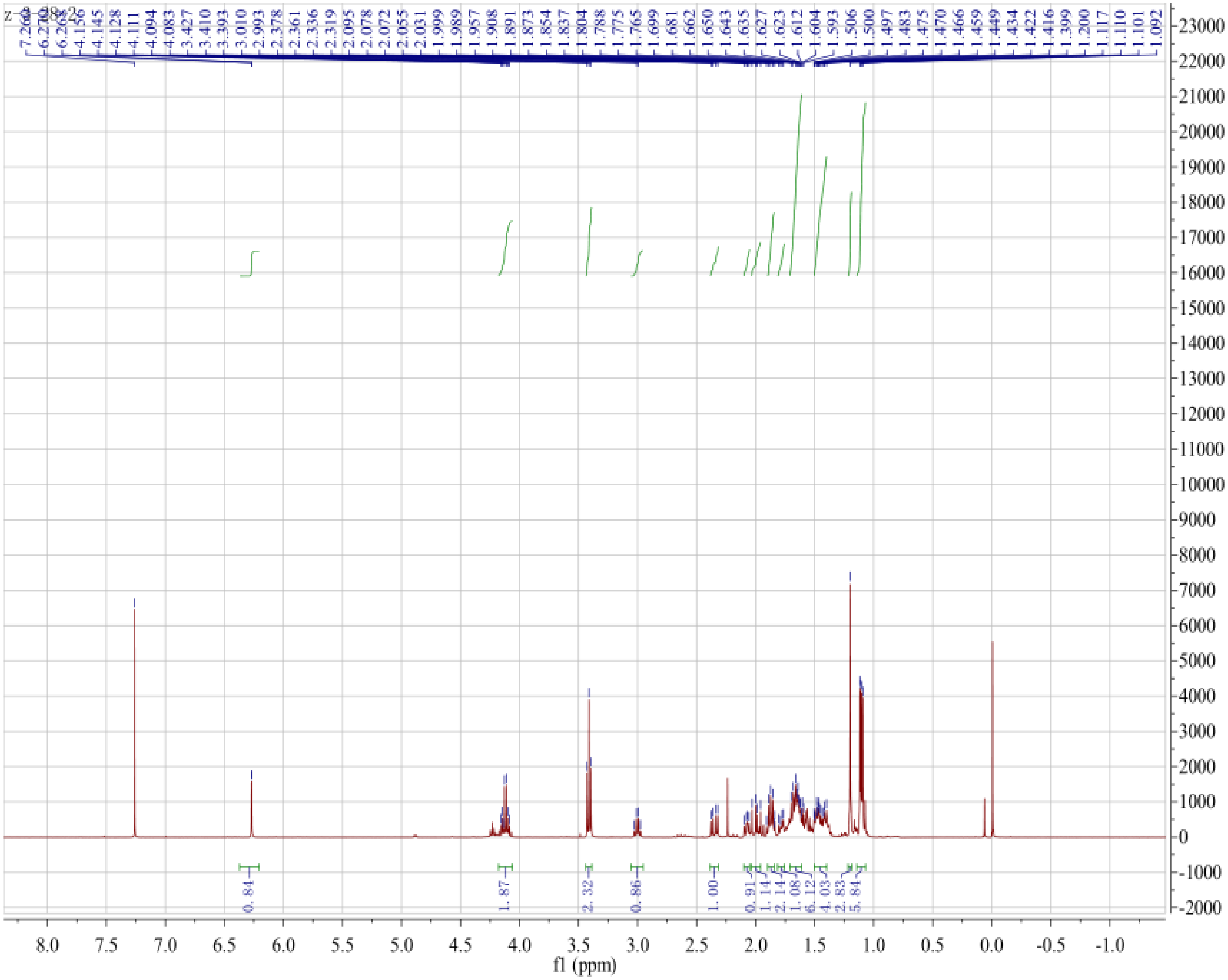

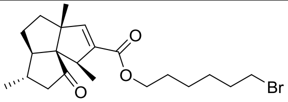

17

Figure S39. ^{13}C NMR (100 MHz, $CDCl_3$) of compound **17**

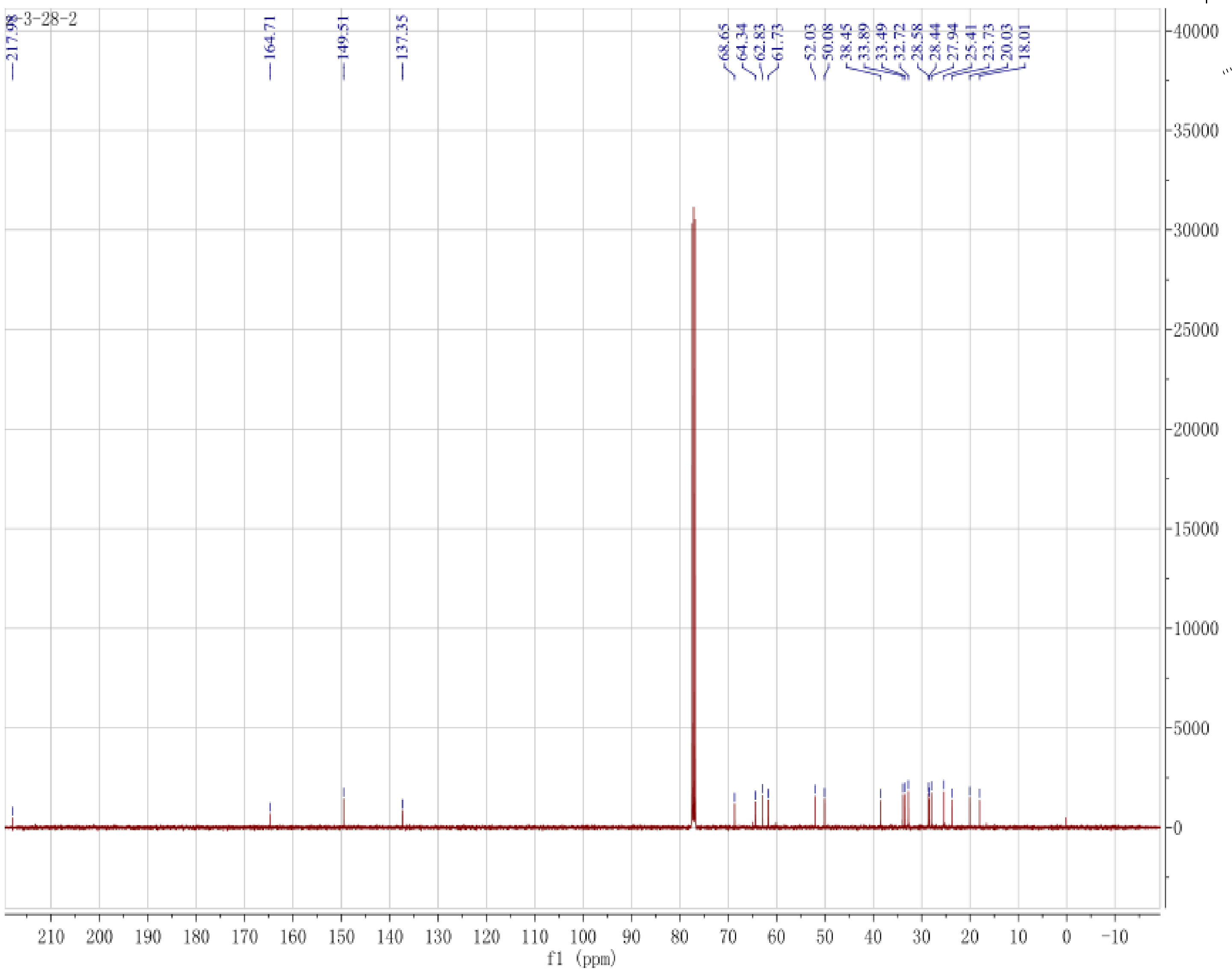

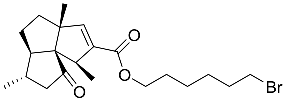

17

Figure S40. ^{1}H NMR (400 MHz, $CDCl_3$) of compound **18**

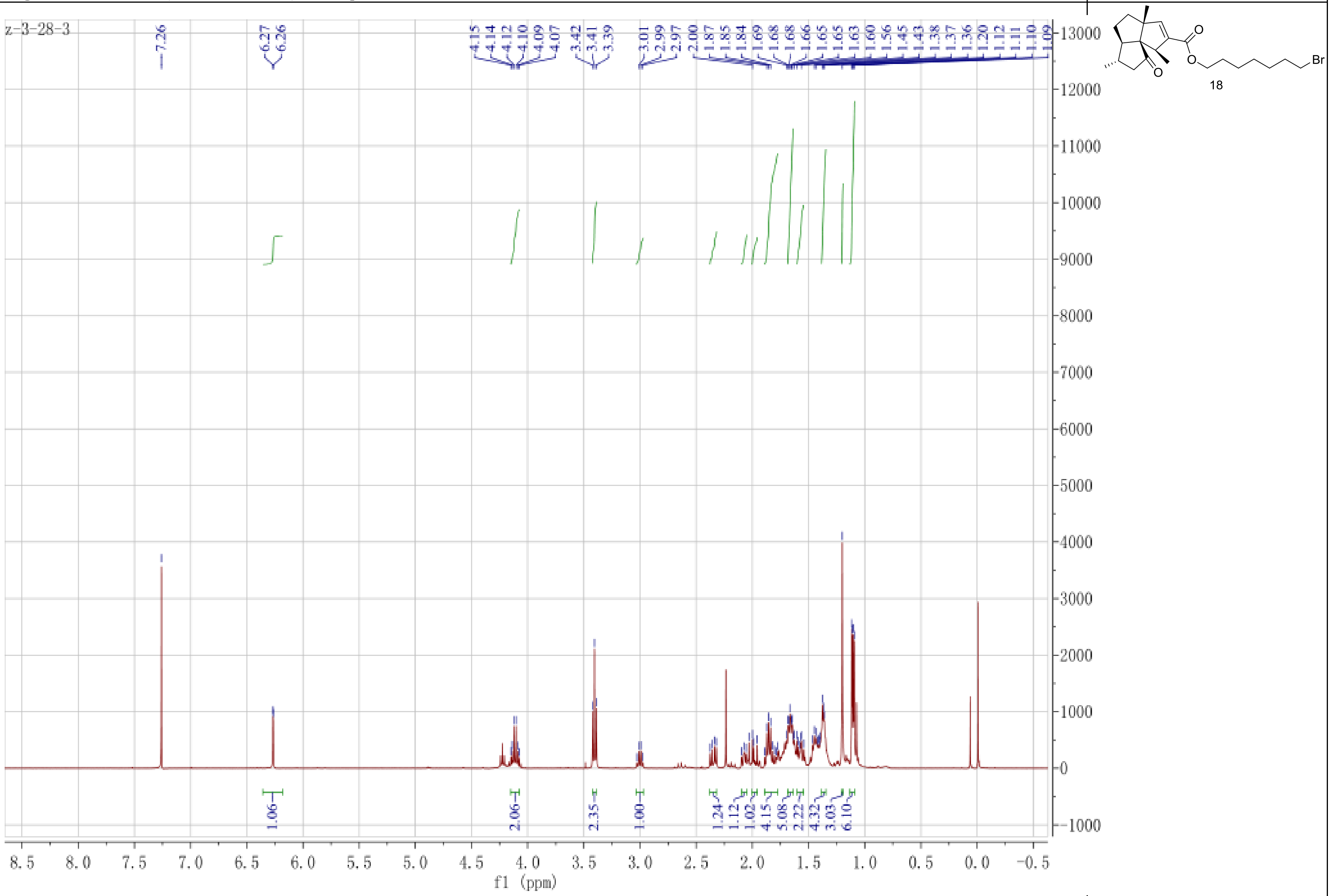

Figure S41. ^{13}C NMR (100 MHz, $CDCl_3$) of compound **18**

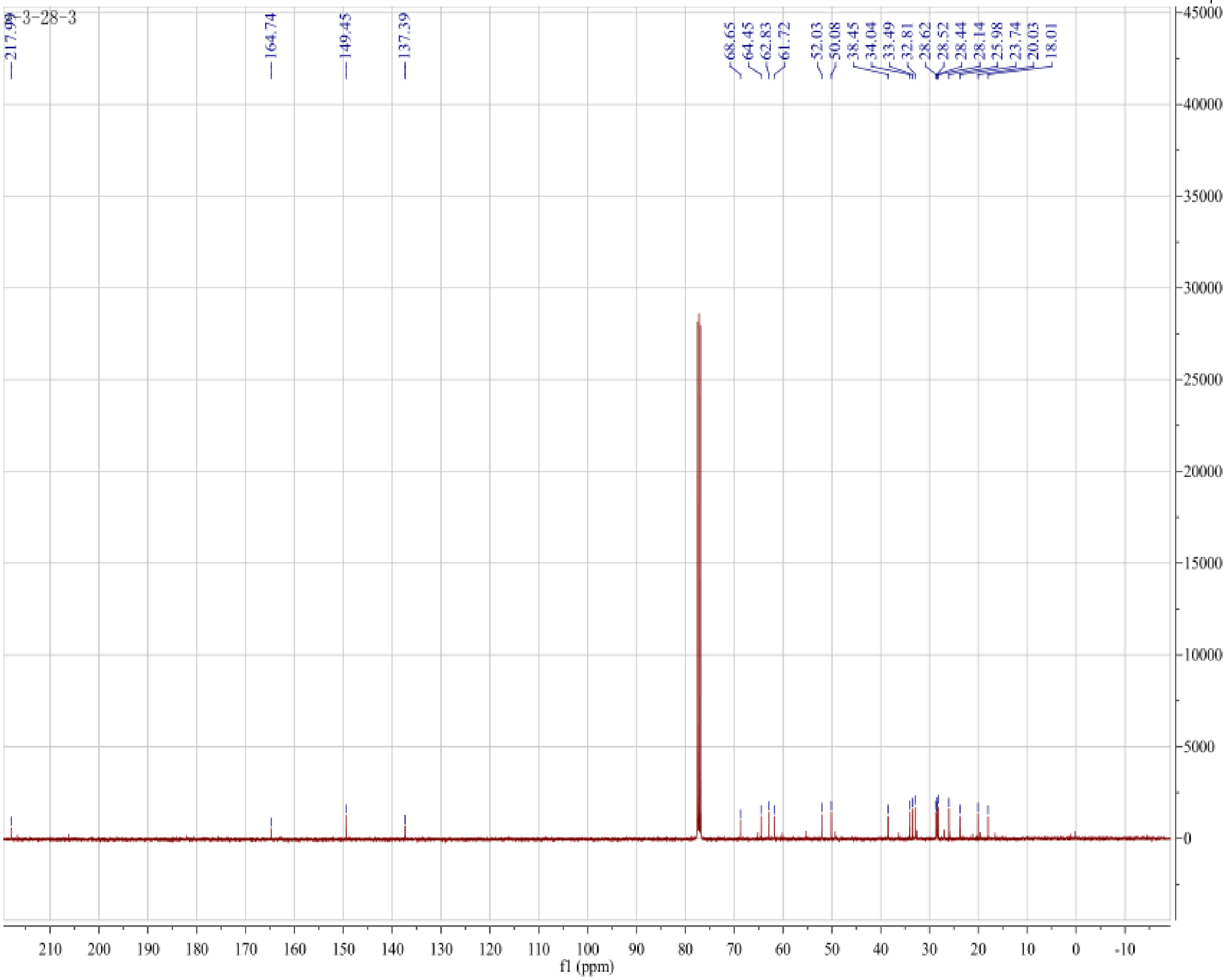

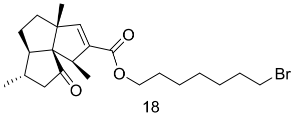

18

Figure S42. ^{1}H NMR (400 MHz, $CDCl_3$) of compound **19**

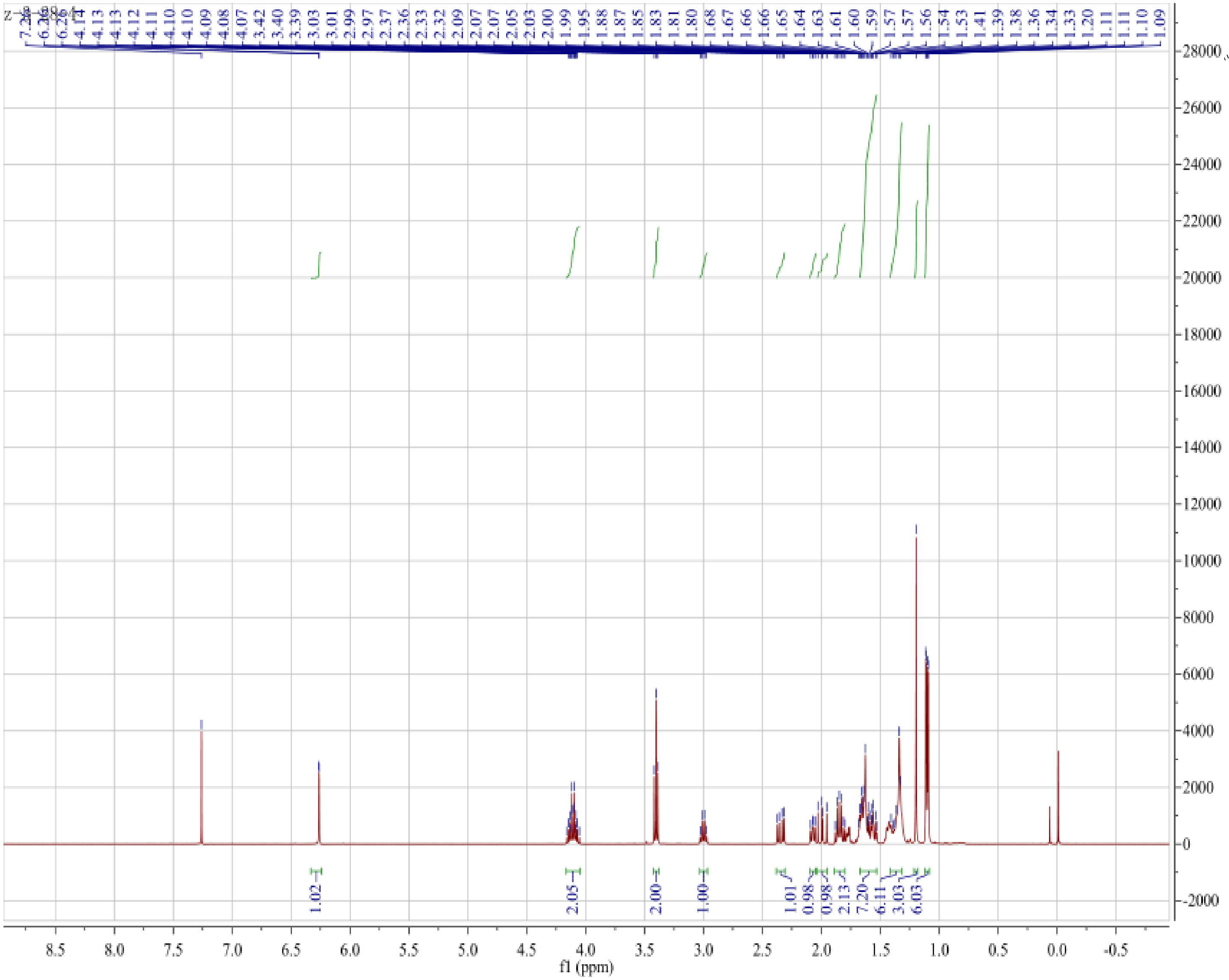

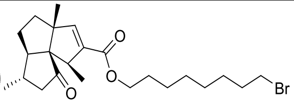

19

Figure S43. ^{13}C NMR (100 MHz, $CDCl_3$) of compound **19**

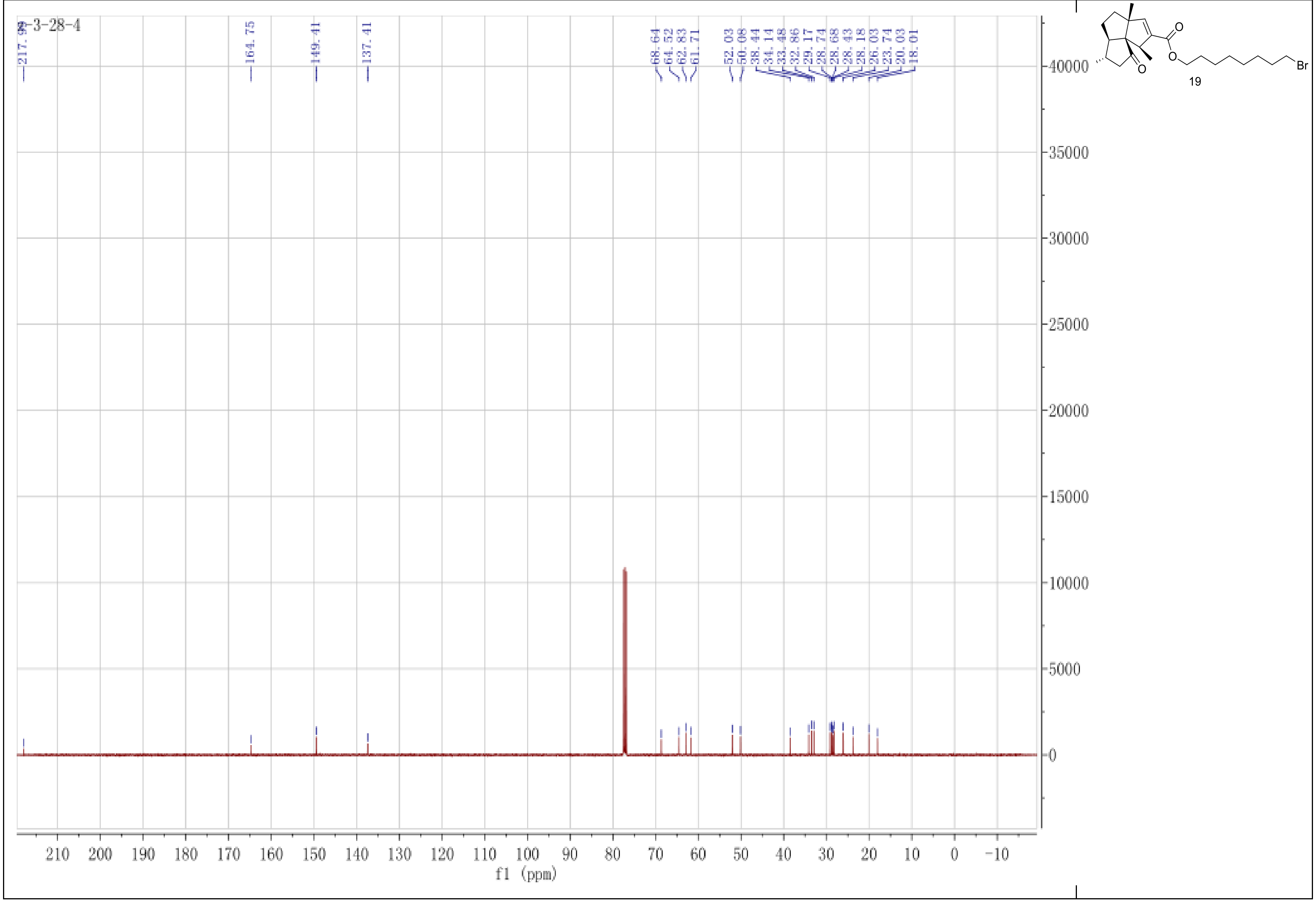

Figure S44. ^{1}H NMR (400 MHz, $CDCl_3$) of compound **20**

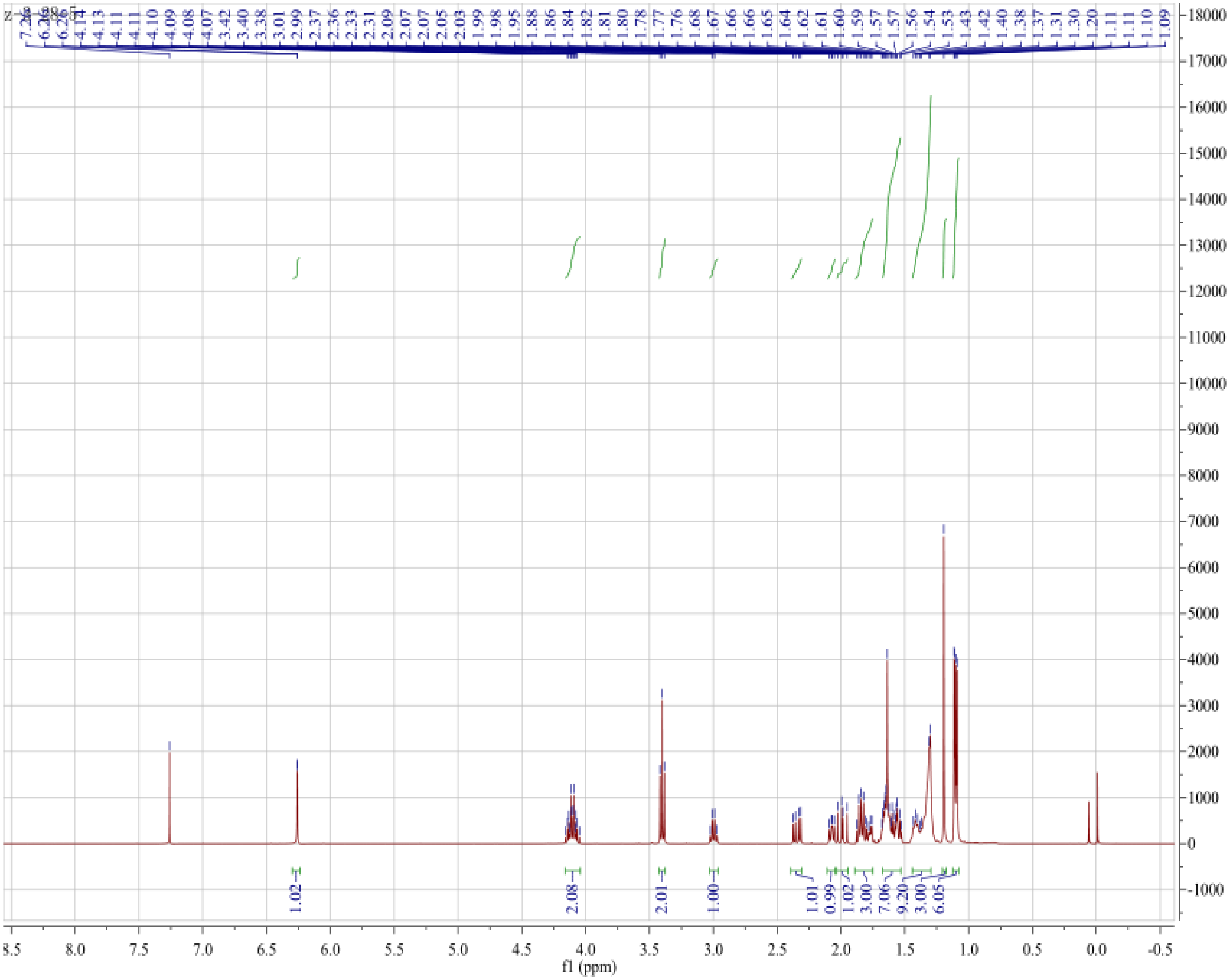

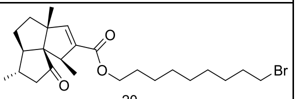

20

Figure S45. ^{13}C NMR (100 MHz, $CDCl_3$) of compound **20**

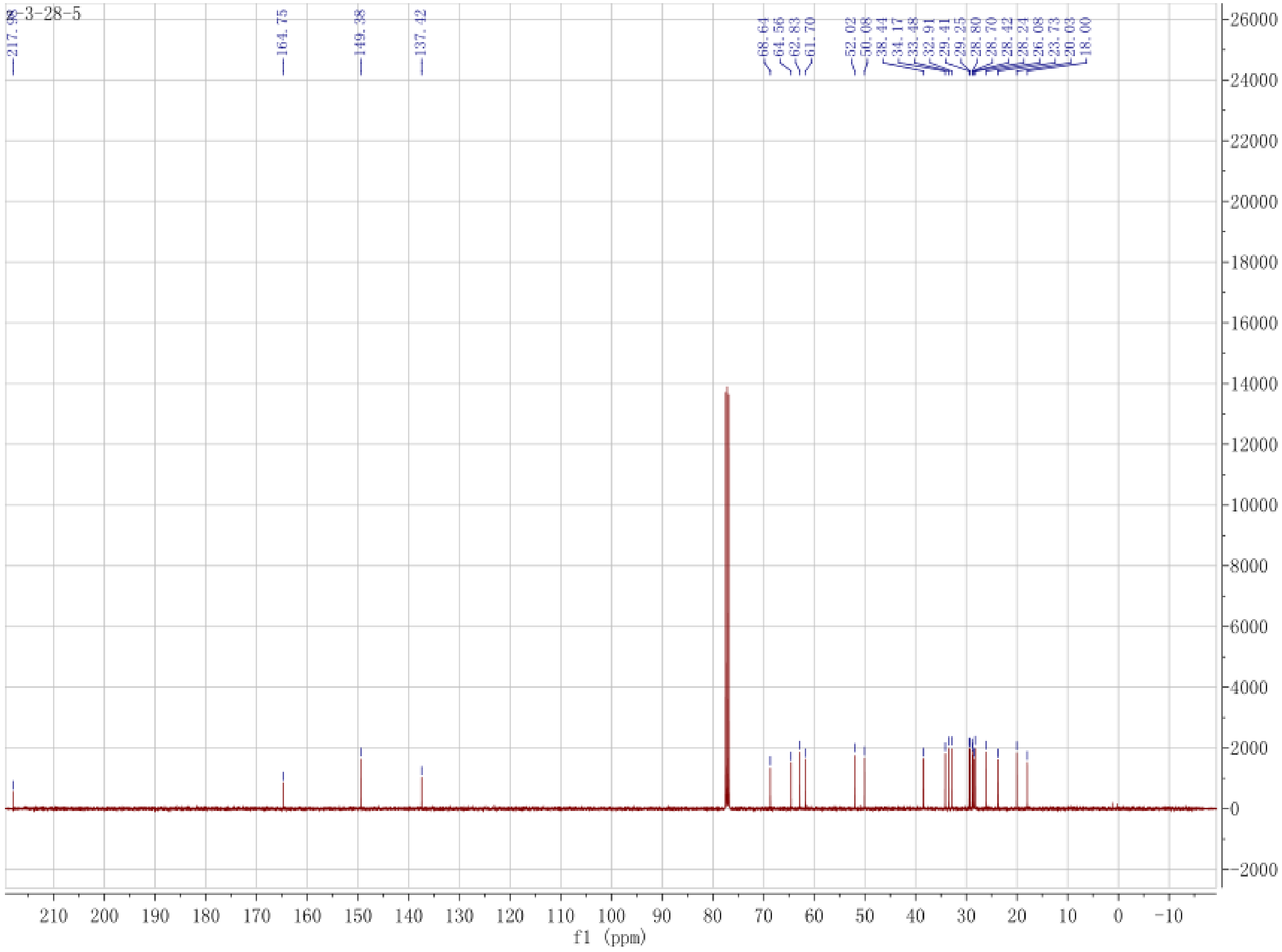

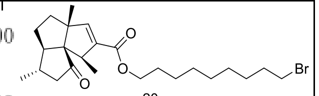

20

Figure S46. ^{1}H NMR (300 MHz, $CDCl_3$) of compound **21**

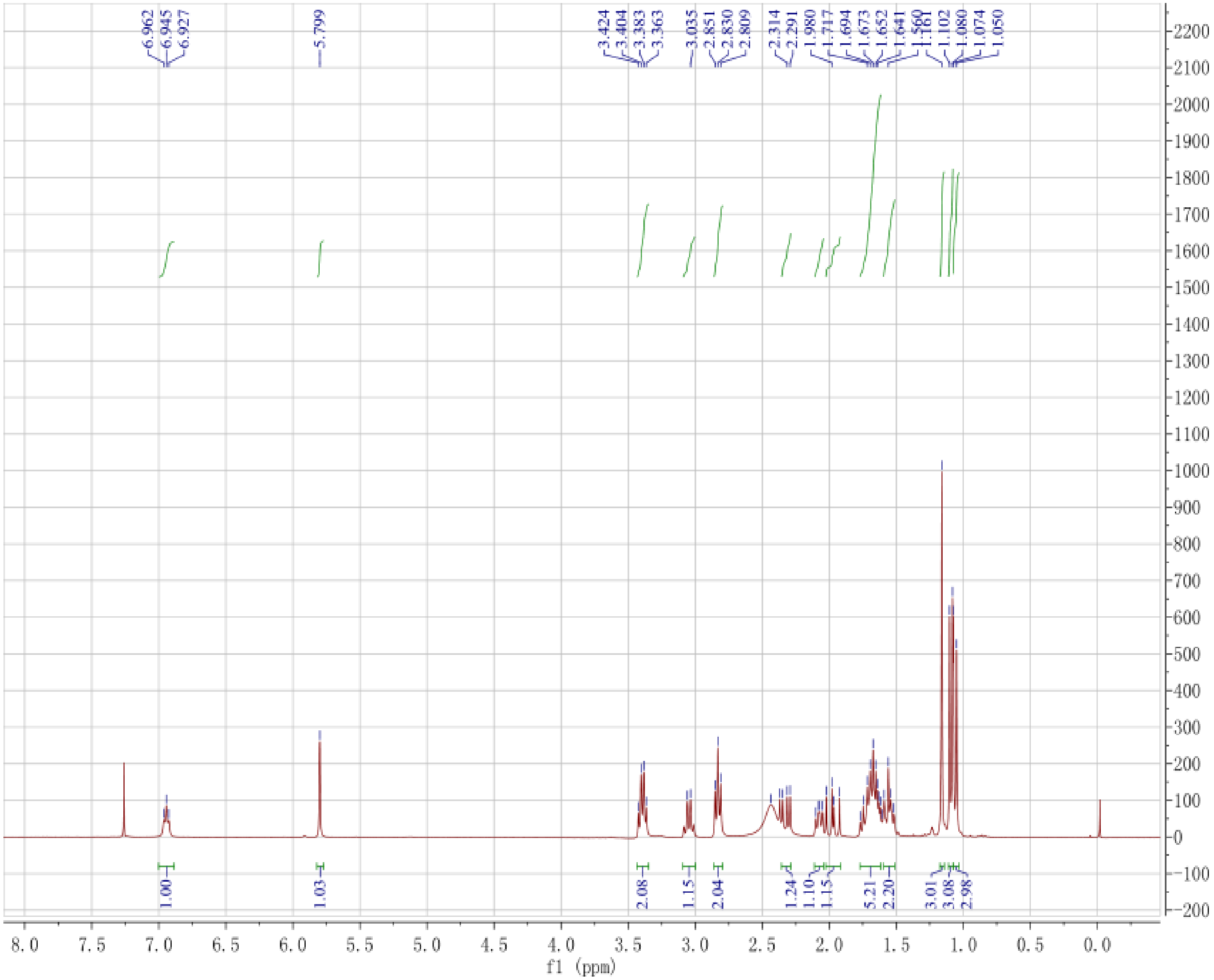

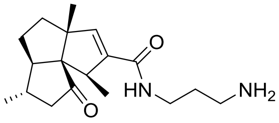
21

Figure S47. ^{13}C NMR (75 MHz, $CDCl_3$) of compound **21**

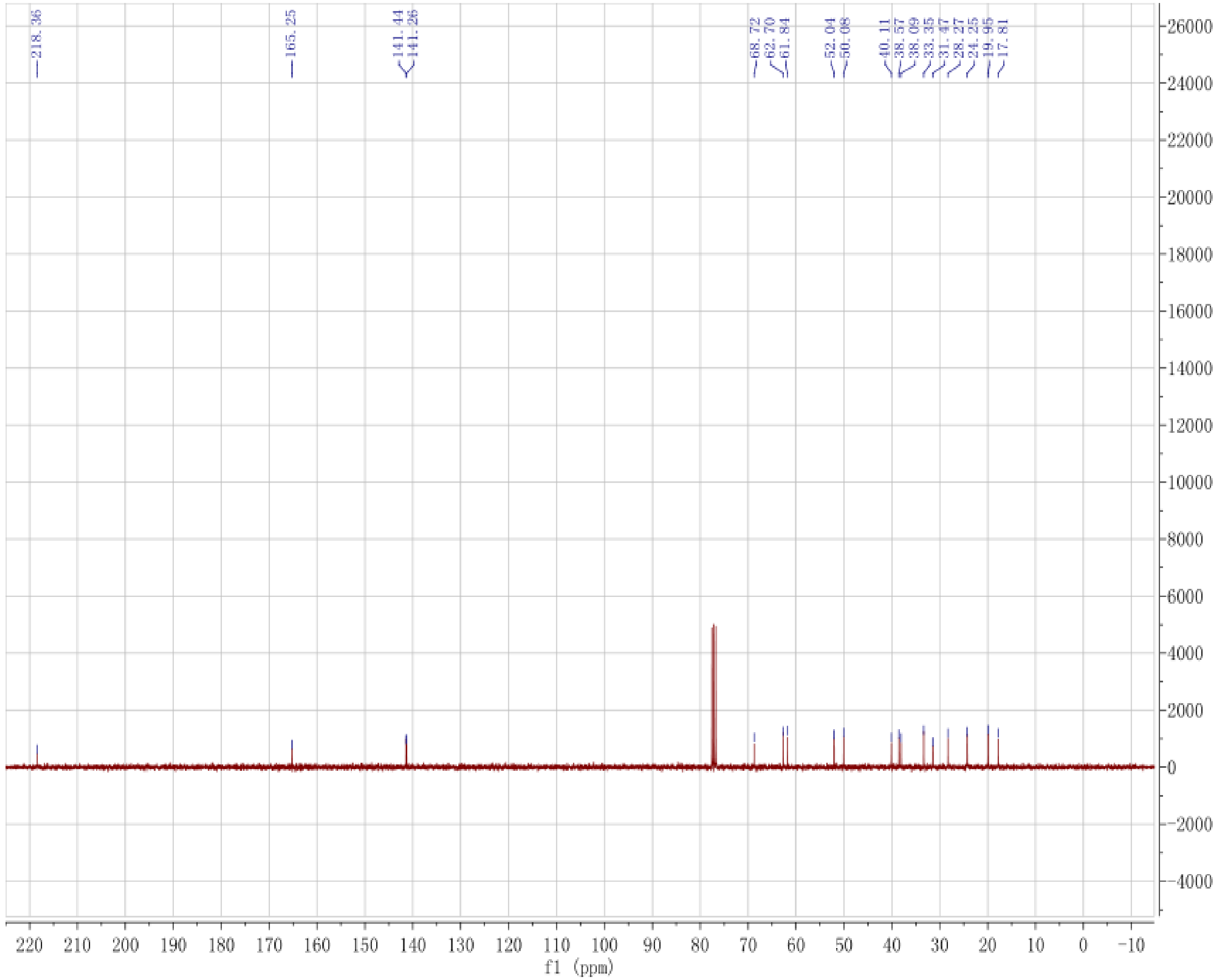

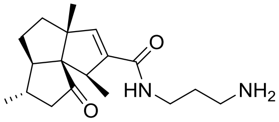

21

Figure S48. ^{1}H NMR (300 MHz, $CDCl_3$) of compound **22**

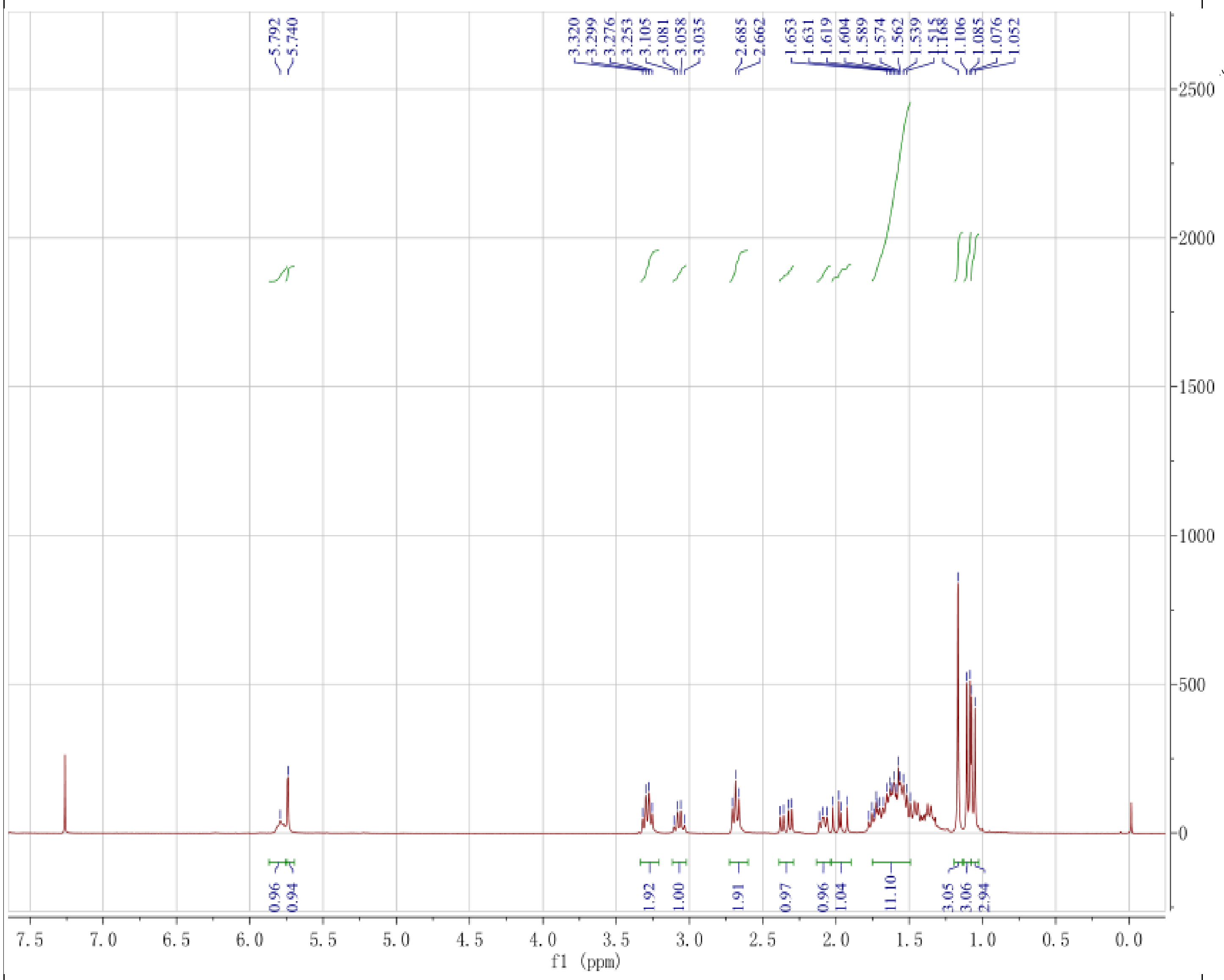

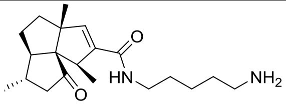

22

Figure S49. ^{13}C NMR (75 MHz, $CDCl_3$) of compound **22**

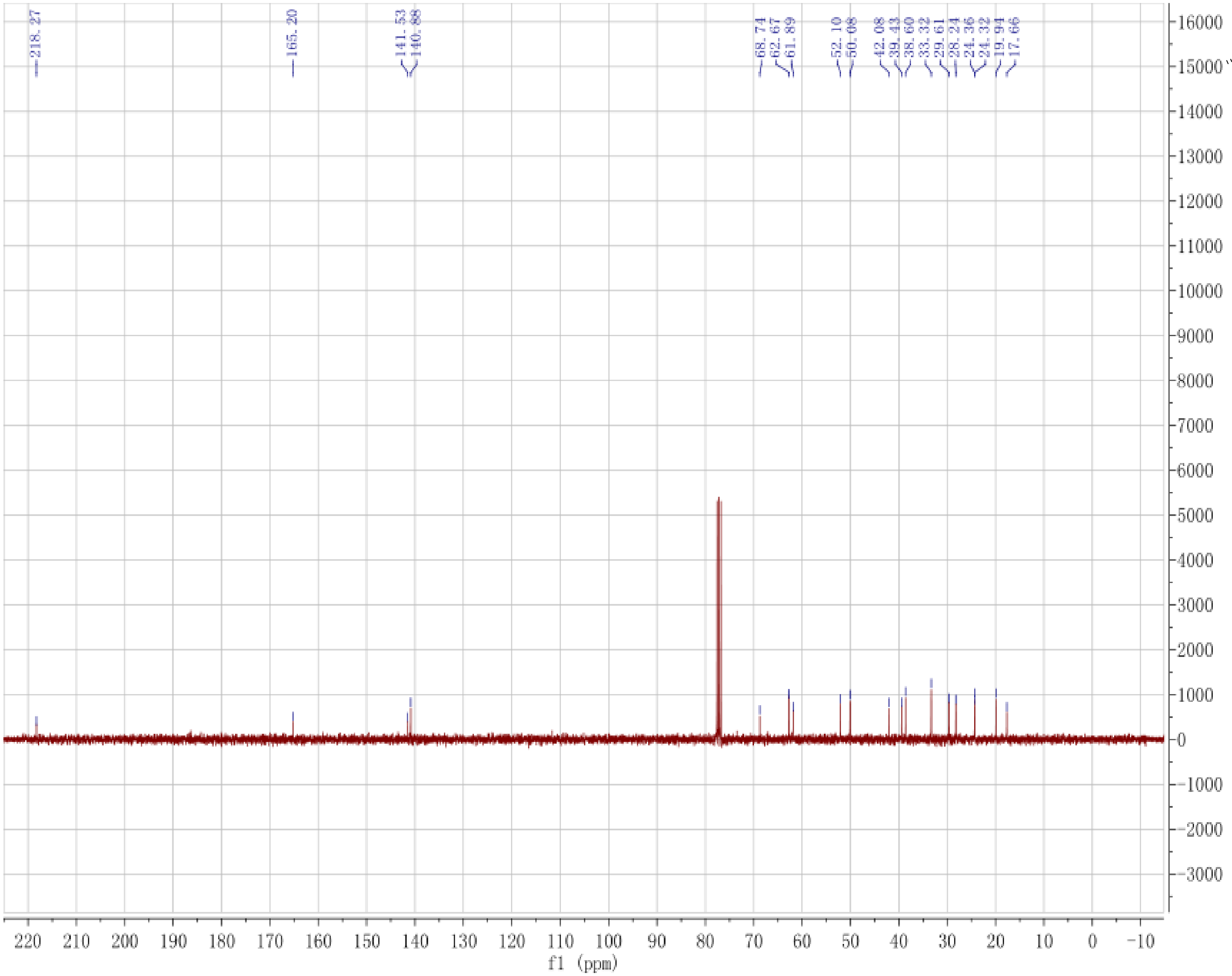

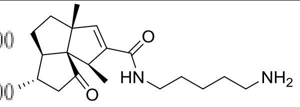

22

Figure S50. ^{1}H NMR (300 MHz, $CDCl_3$) of compound **23**

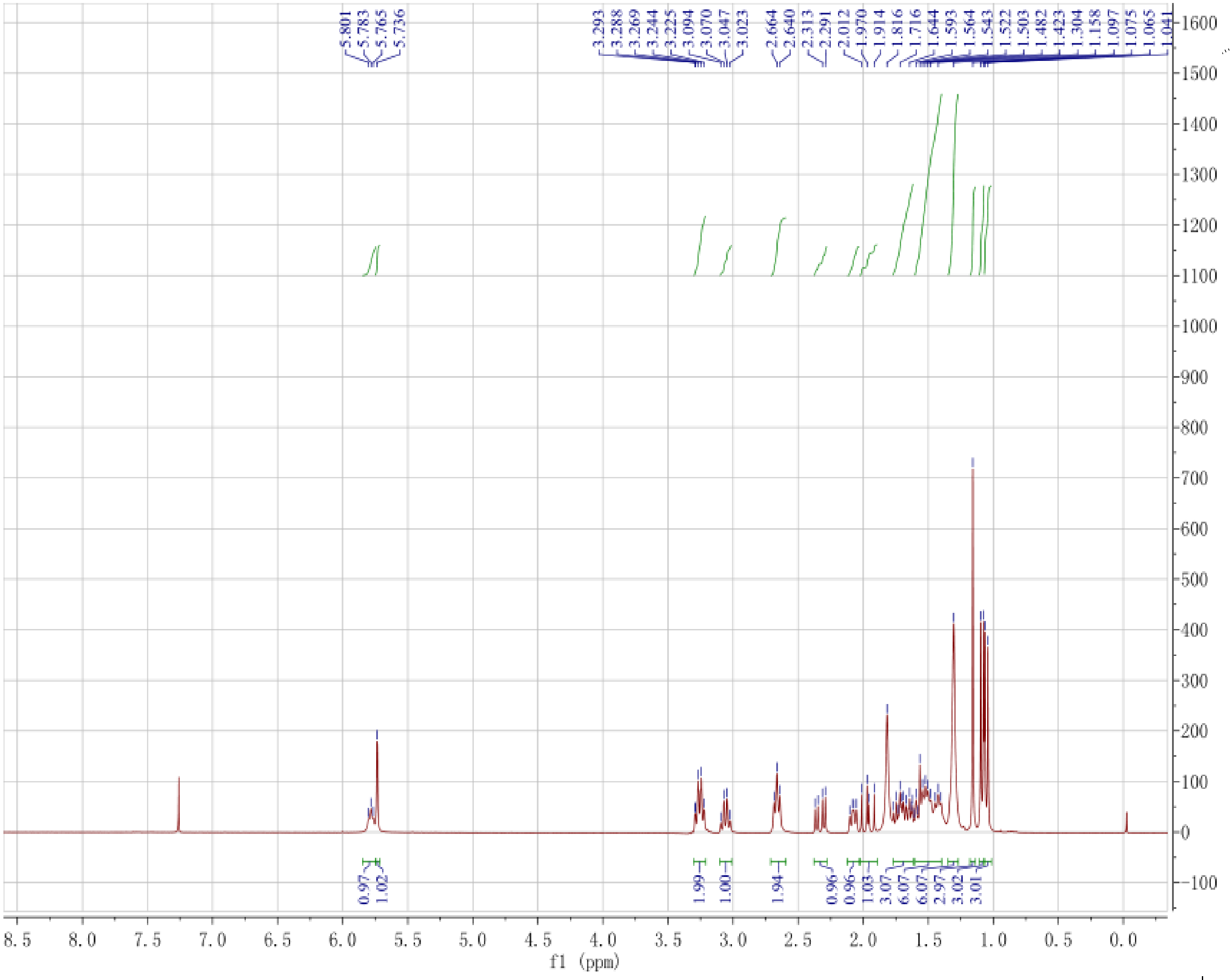

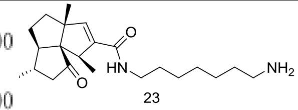

23

Figure S51. ^{13}C NMR (75 MHz, CDCl$_3$) of compound **23**

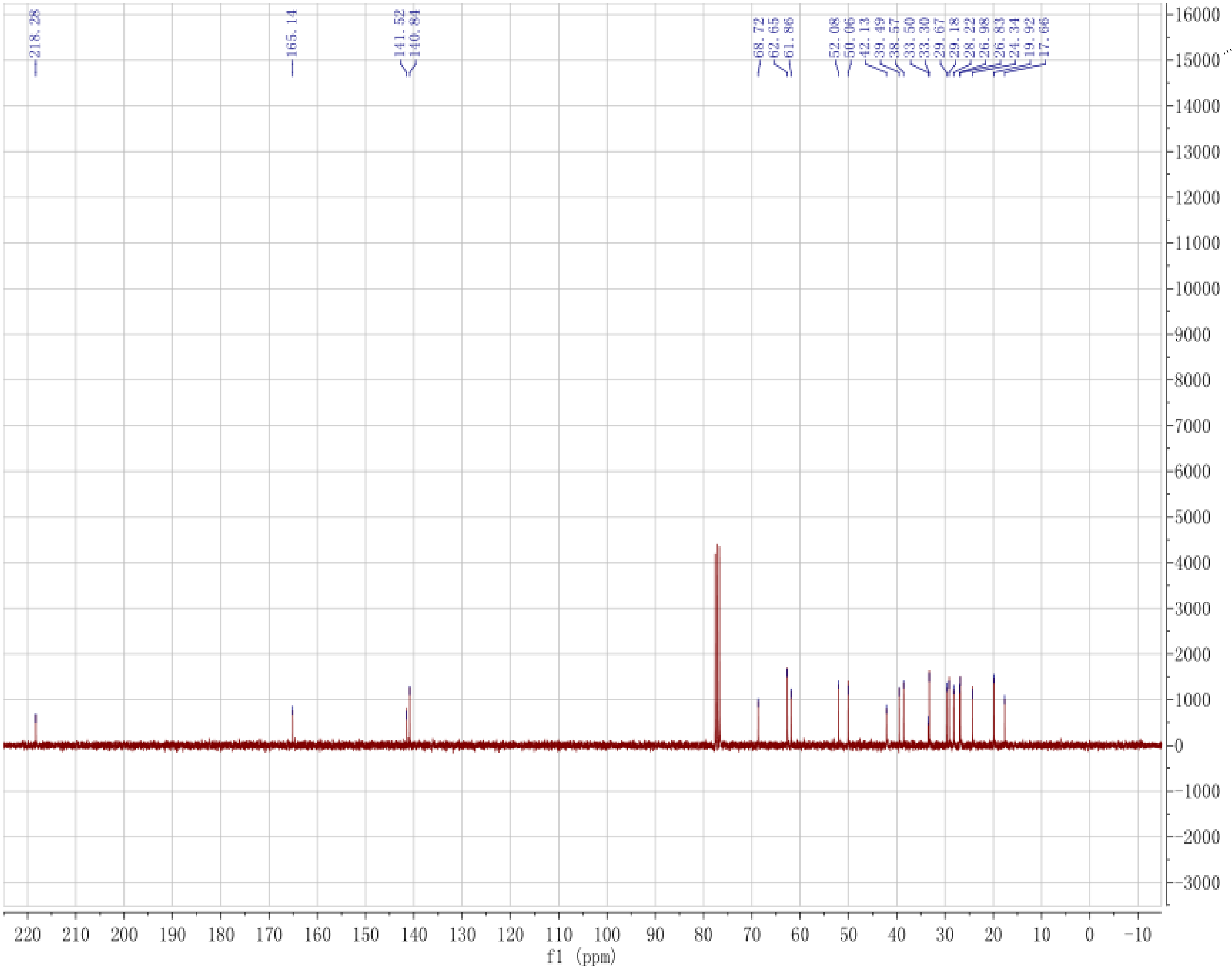

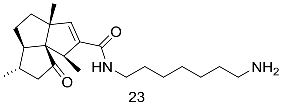

23

Figure S52. ^{1}H NMR (300 MHz, $CDCl_3$) of compound **24**

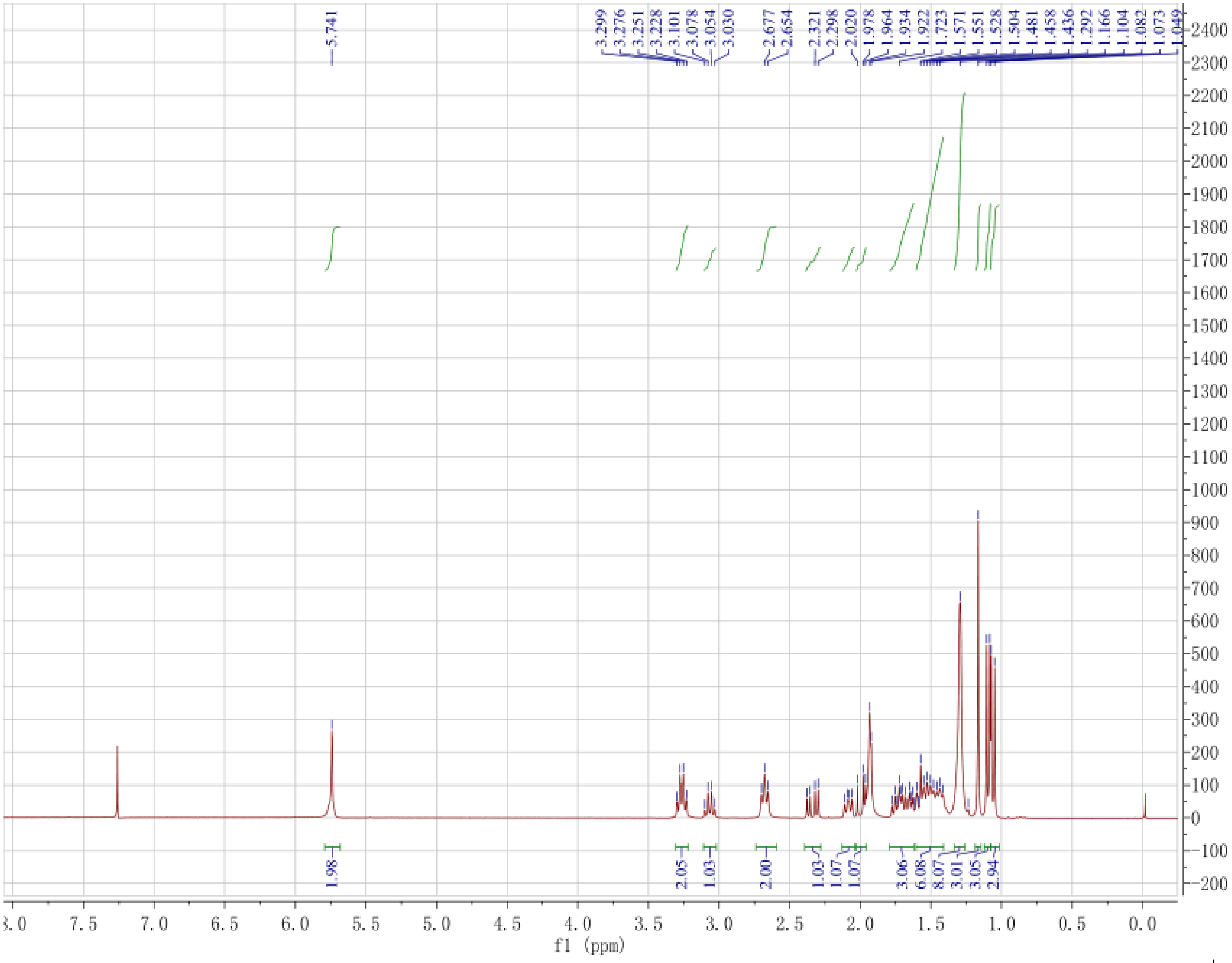

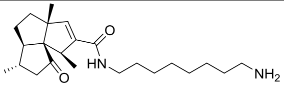

24

Figure S53. ^{13}C NMR (75 MHz, $CDCl_3$) of compound **24**

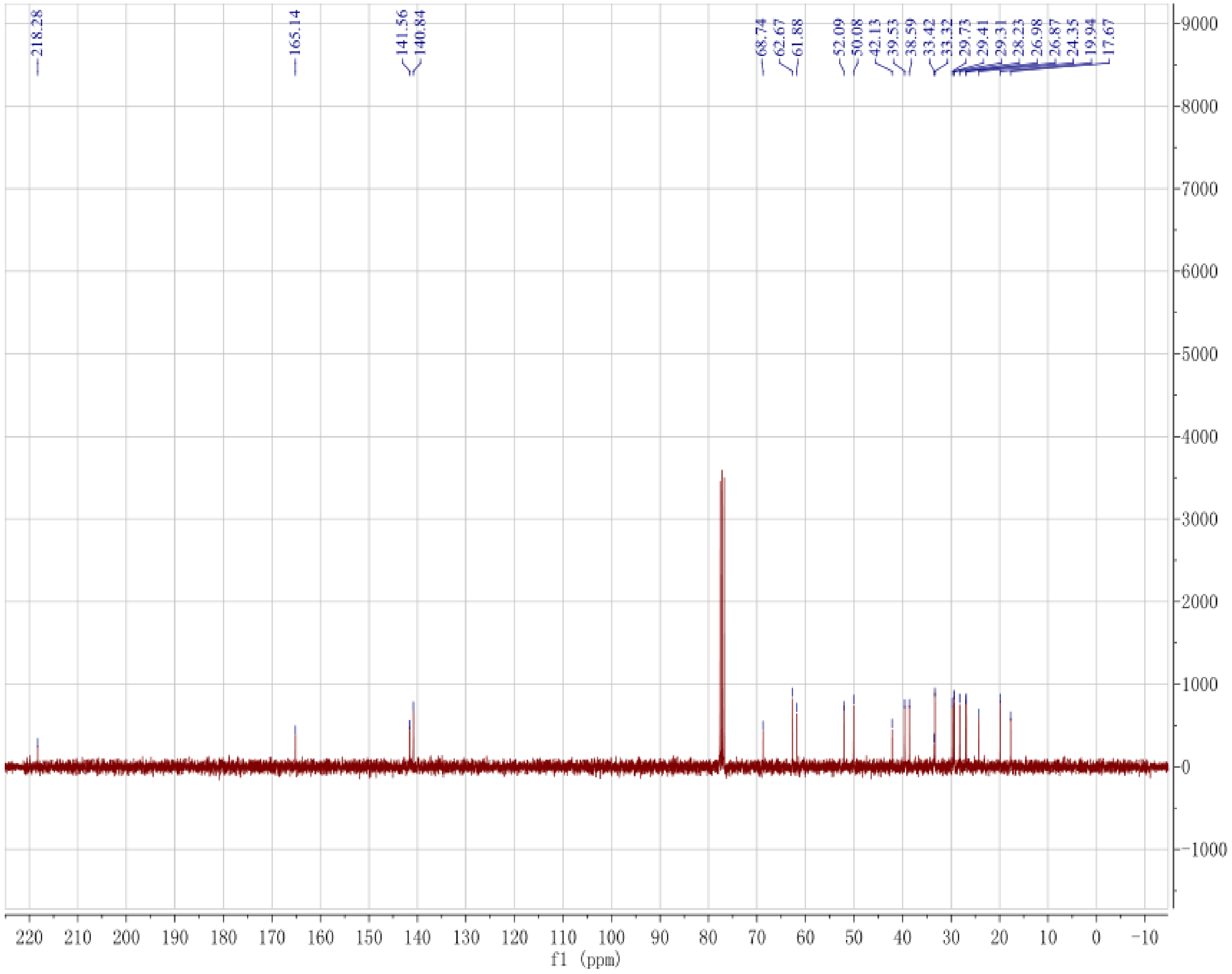

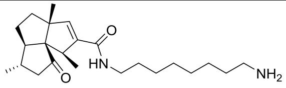

24

Figure S54. ^{1}H NMR (300 MHz, CDCl$_3$) of compound **25**

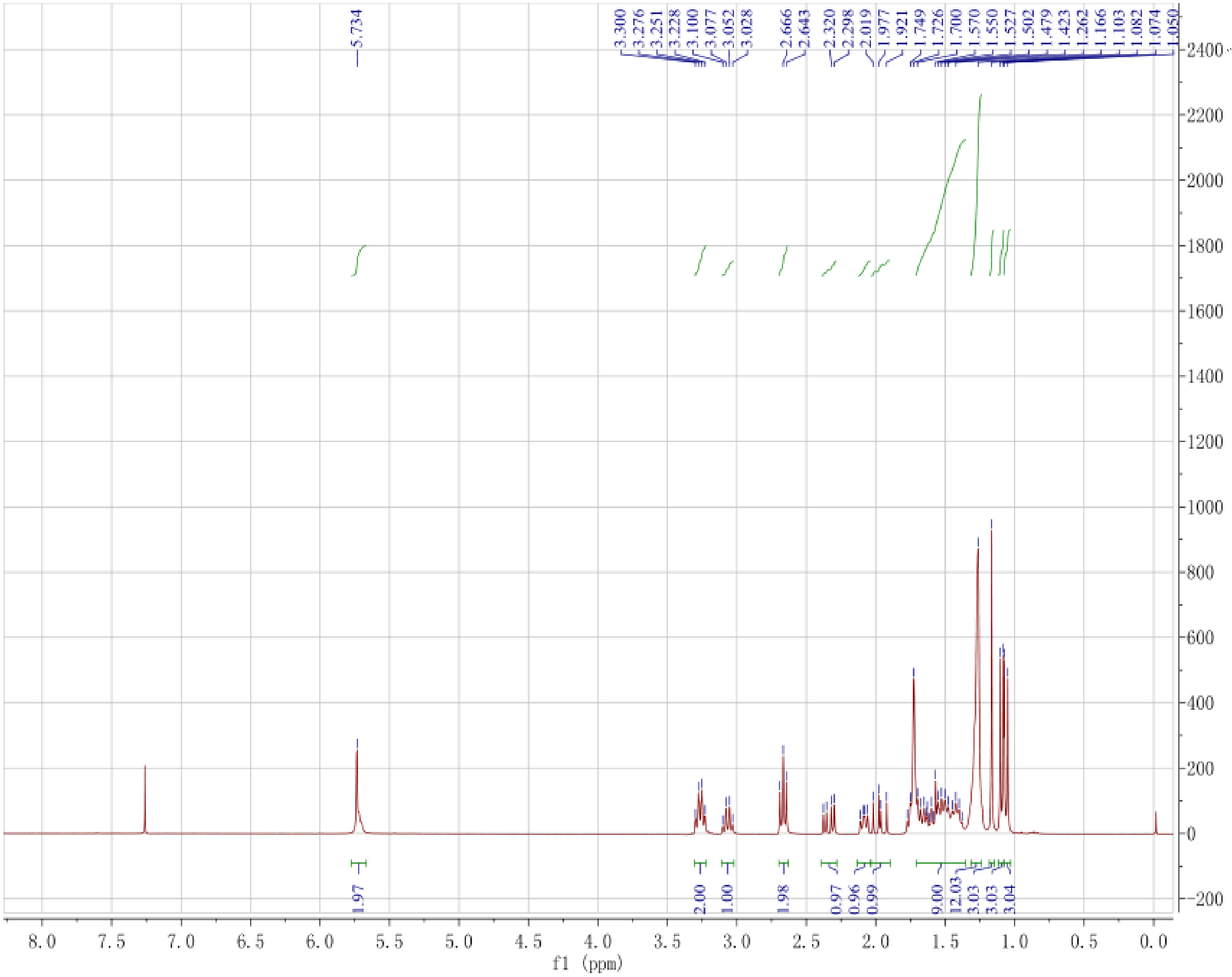

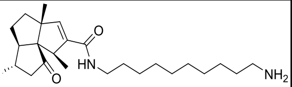

25

Figure S55. ^{13}C NMR (75 MHz, $CDCl_3$) of compound **25**

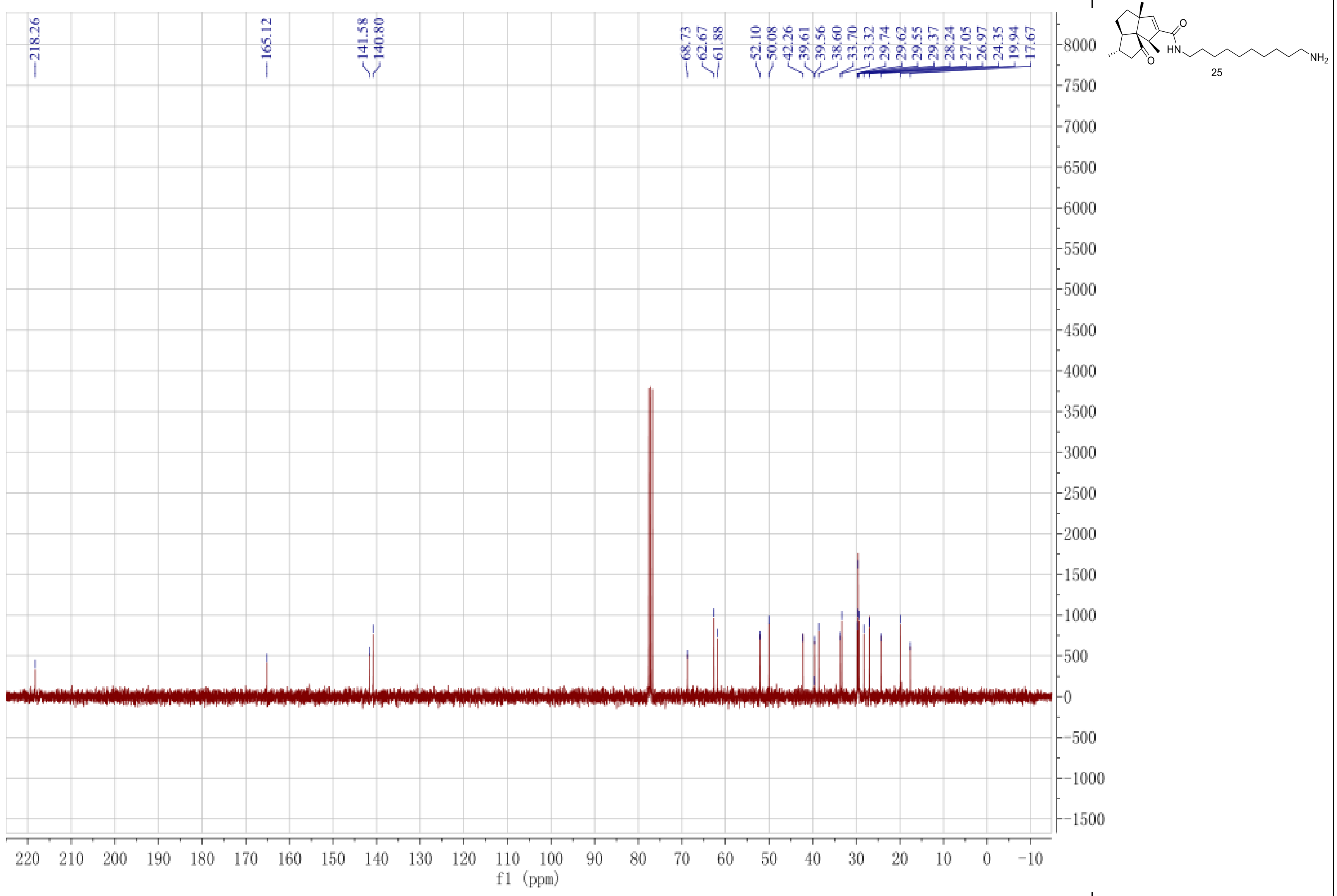

Figure S56. ^{1}H NMR (300 MHz, CDCl₃) of compound **26**

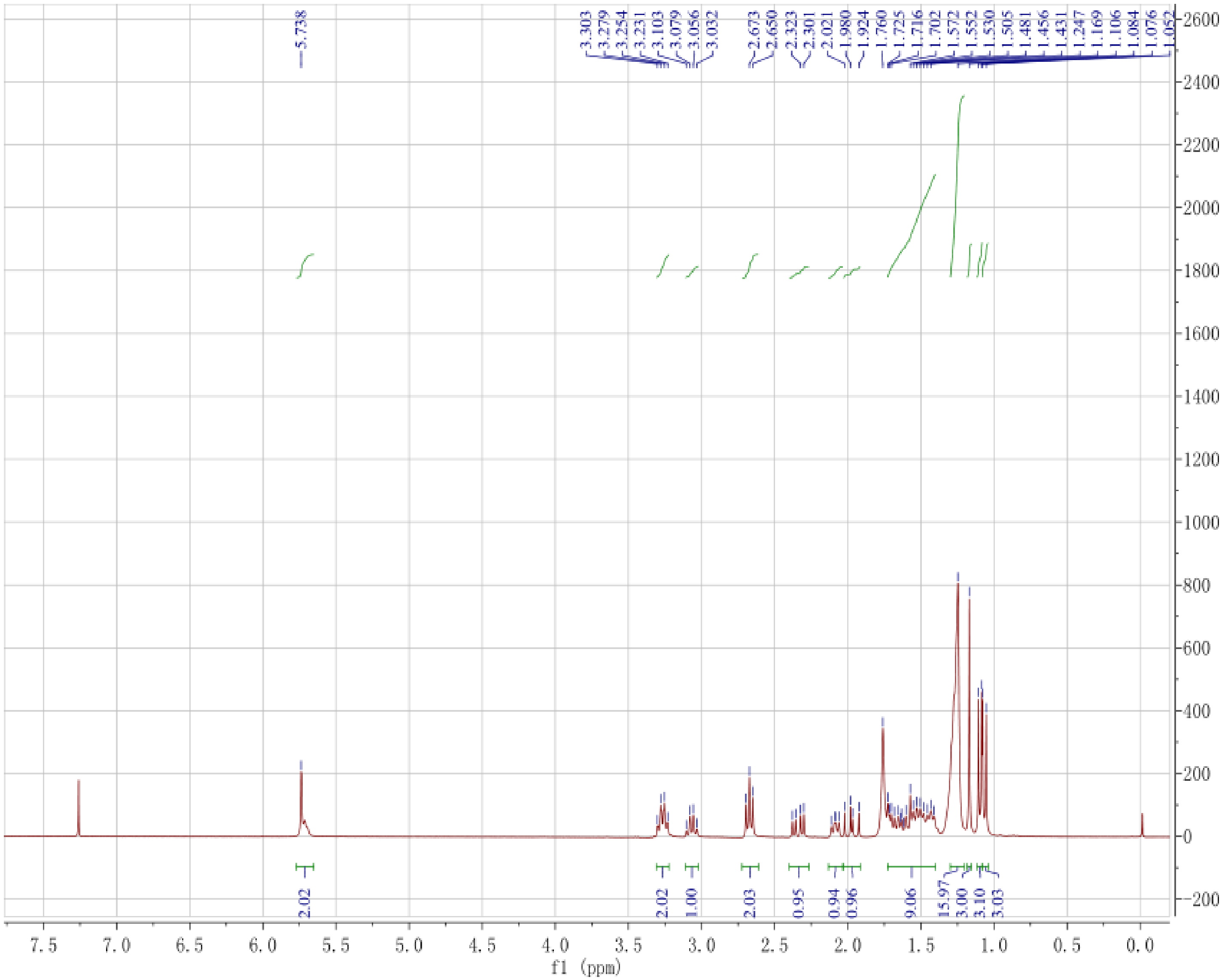

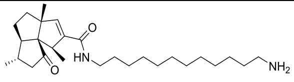

26

Figure S57. ^{13}C NMR (75 MHz, $CDCl_3$) of compound **26**

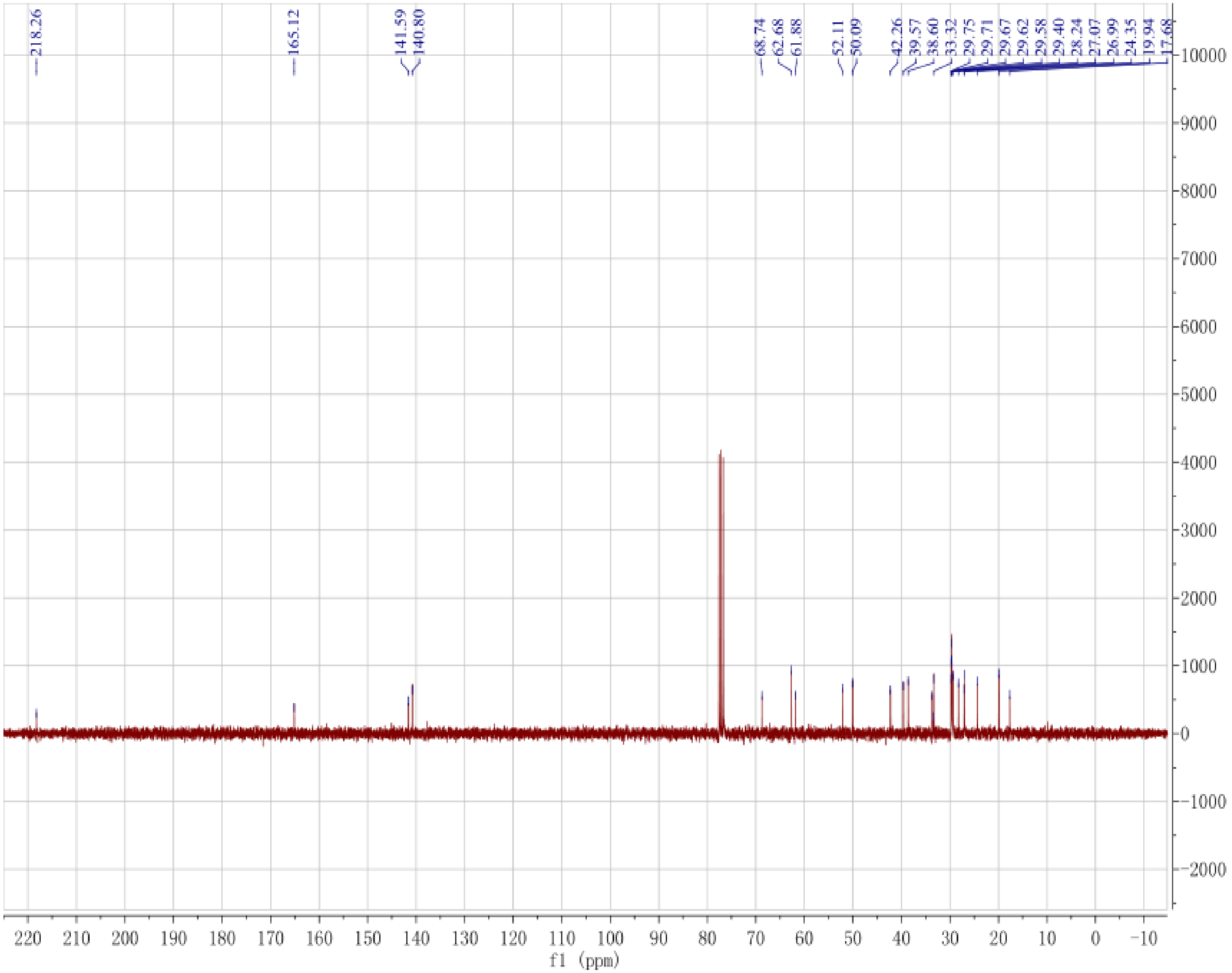

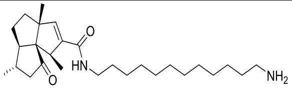

26

Figure S58. HRMS of compound **4**

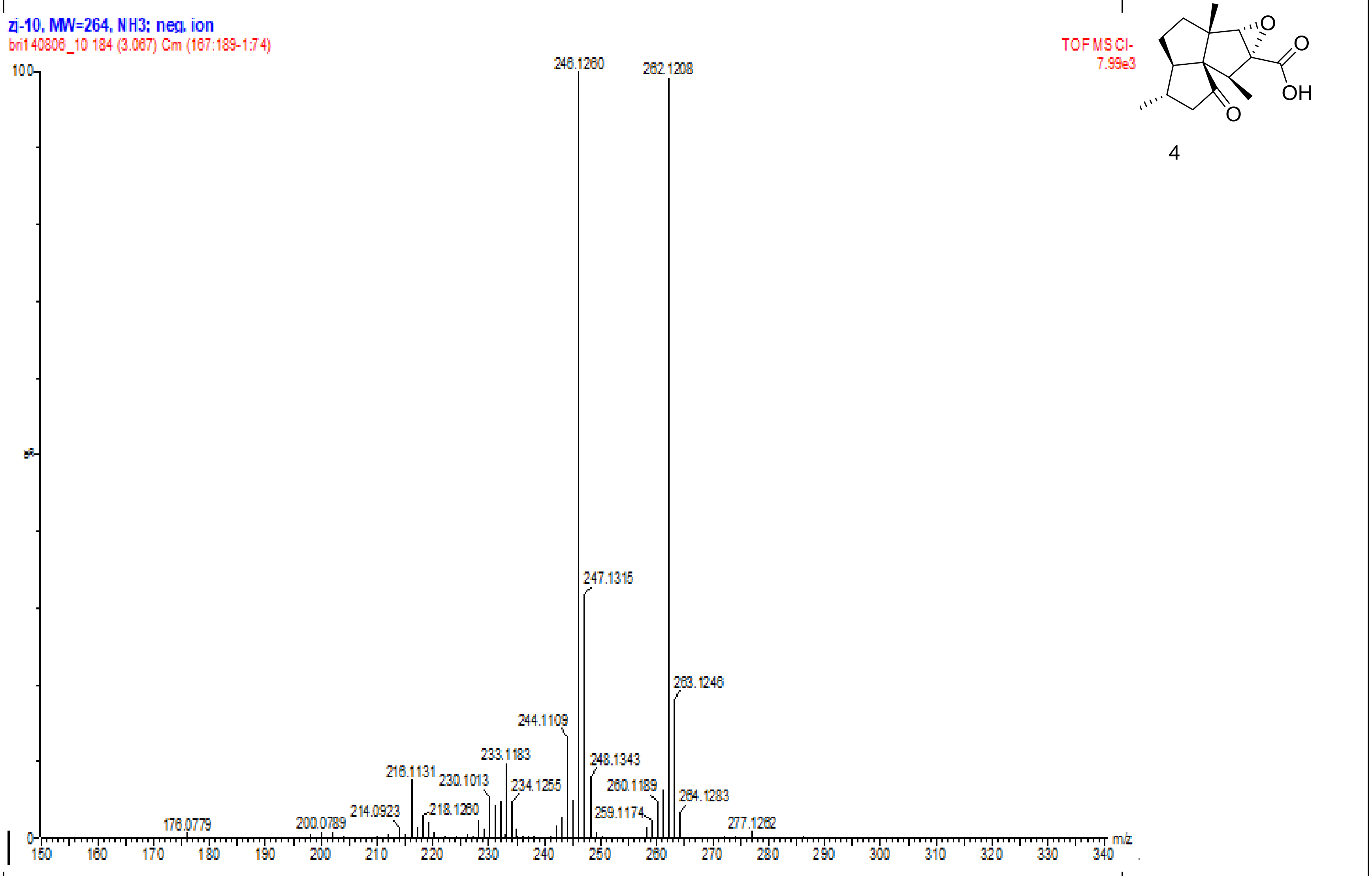

Figure S59. HRMS of compound **5**

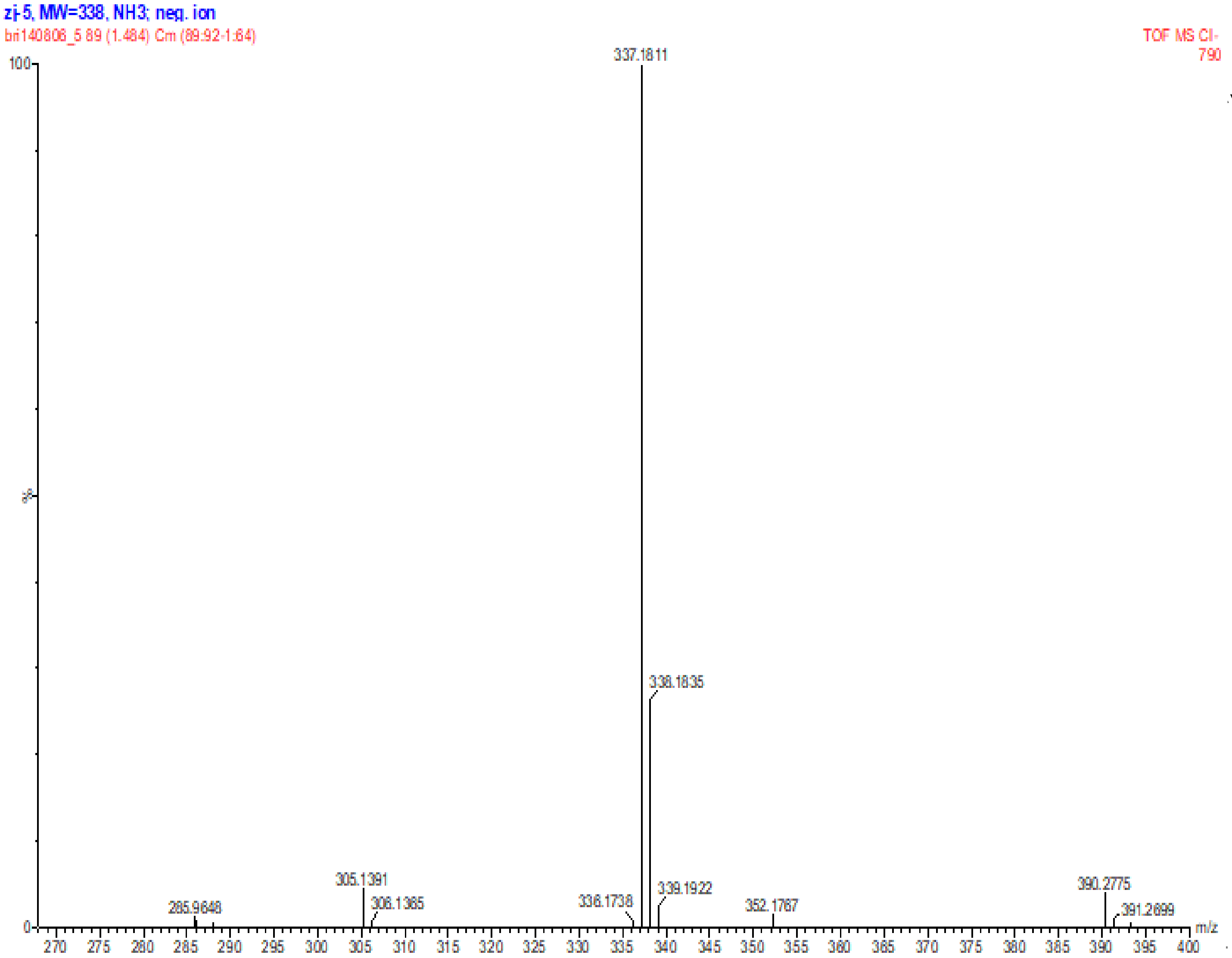

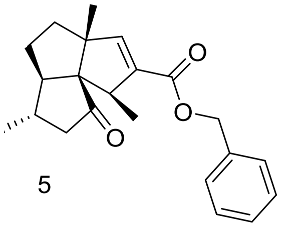

5

Figure S60. HRMS of compound **8**

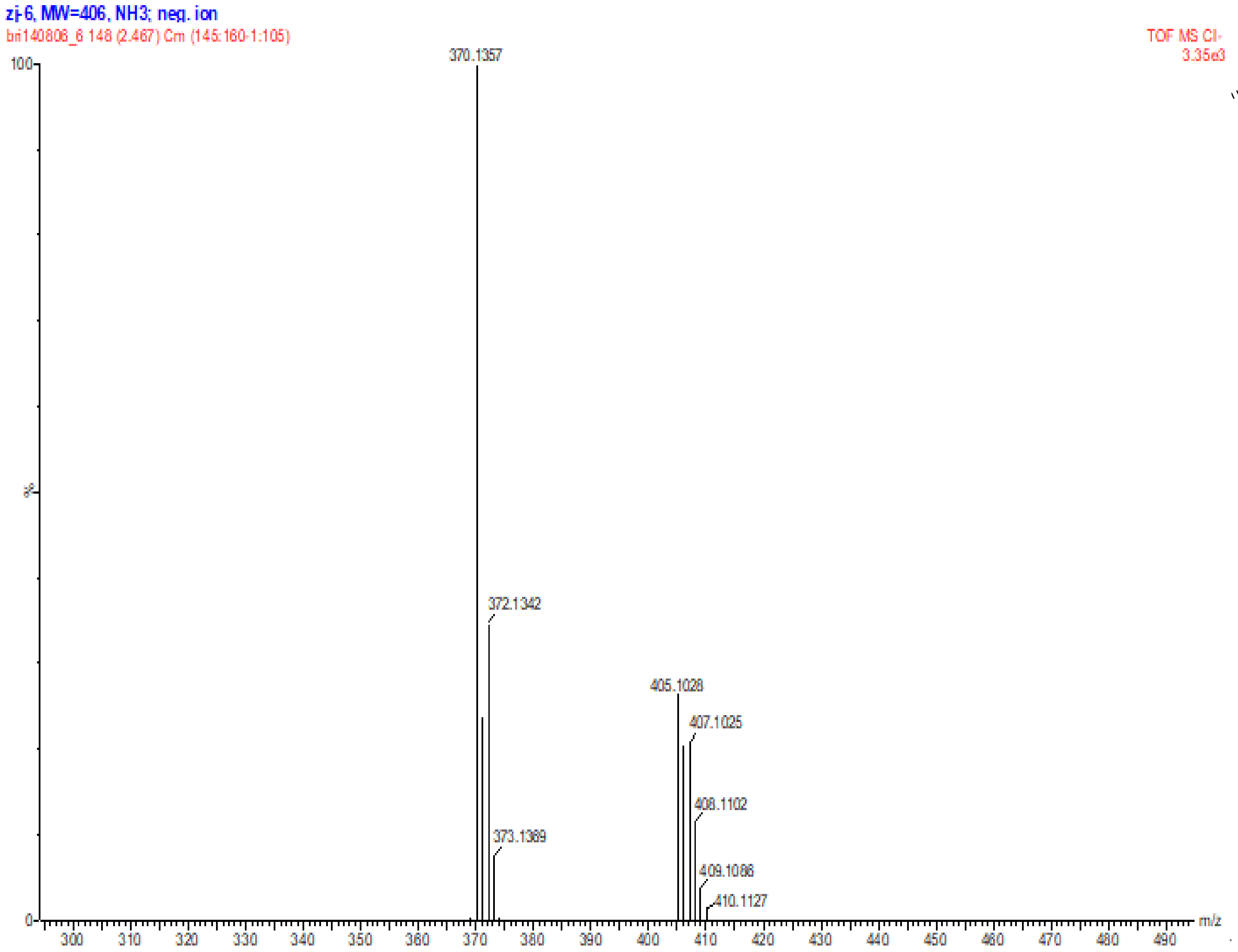

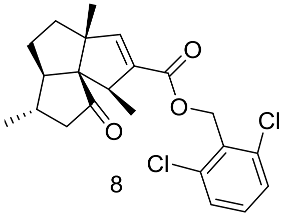

8

Figure S61. HRMS of compound **10**

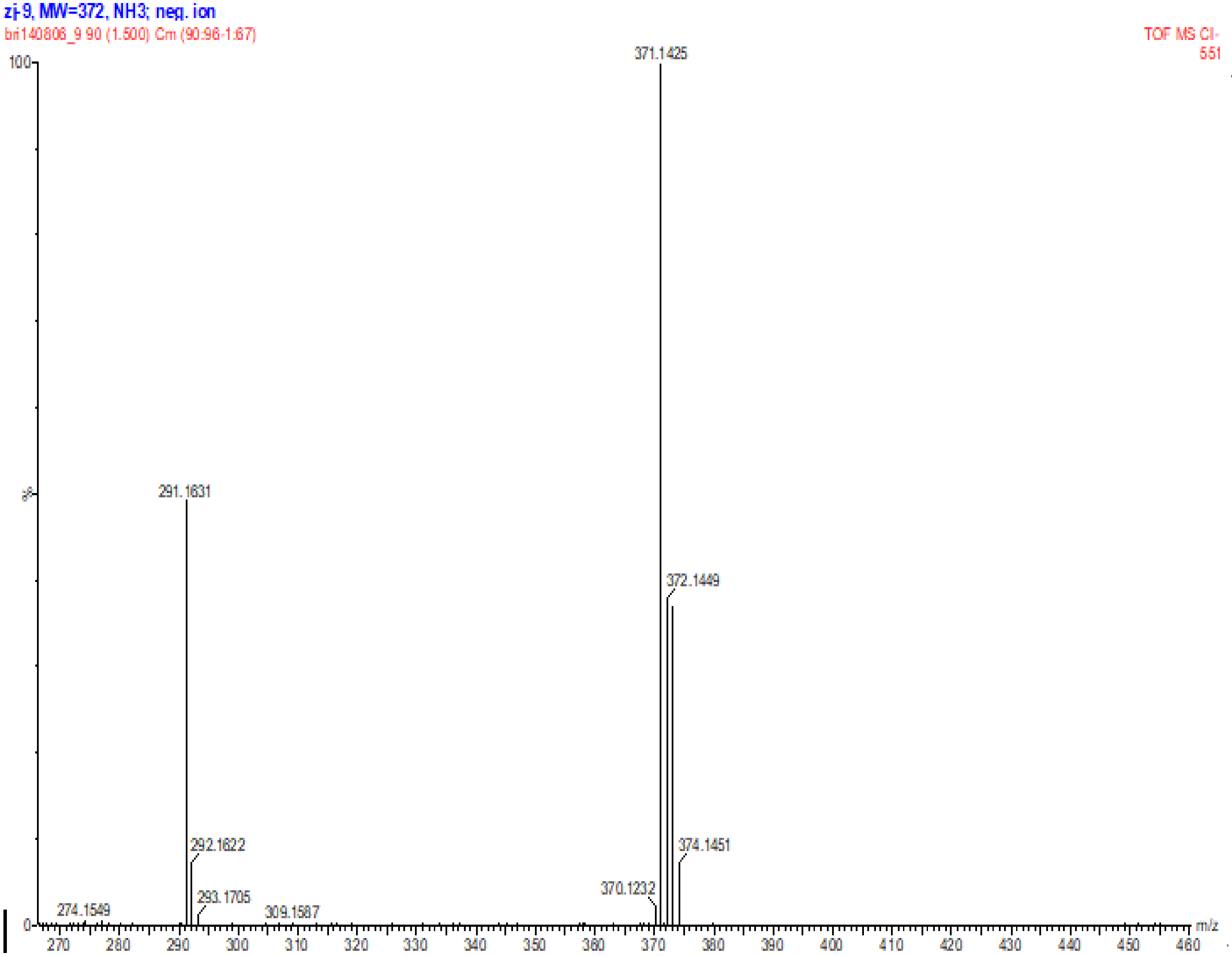

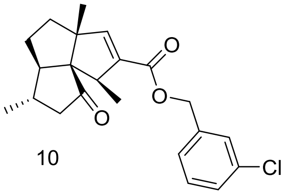

10

Figure S62. HRMS of compound **11**

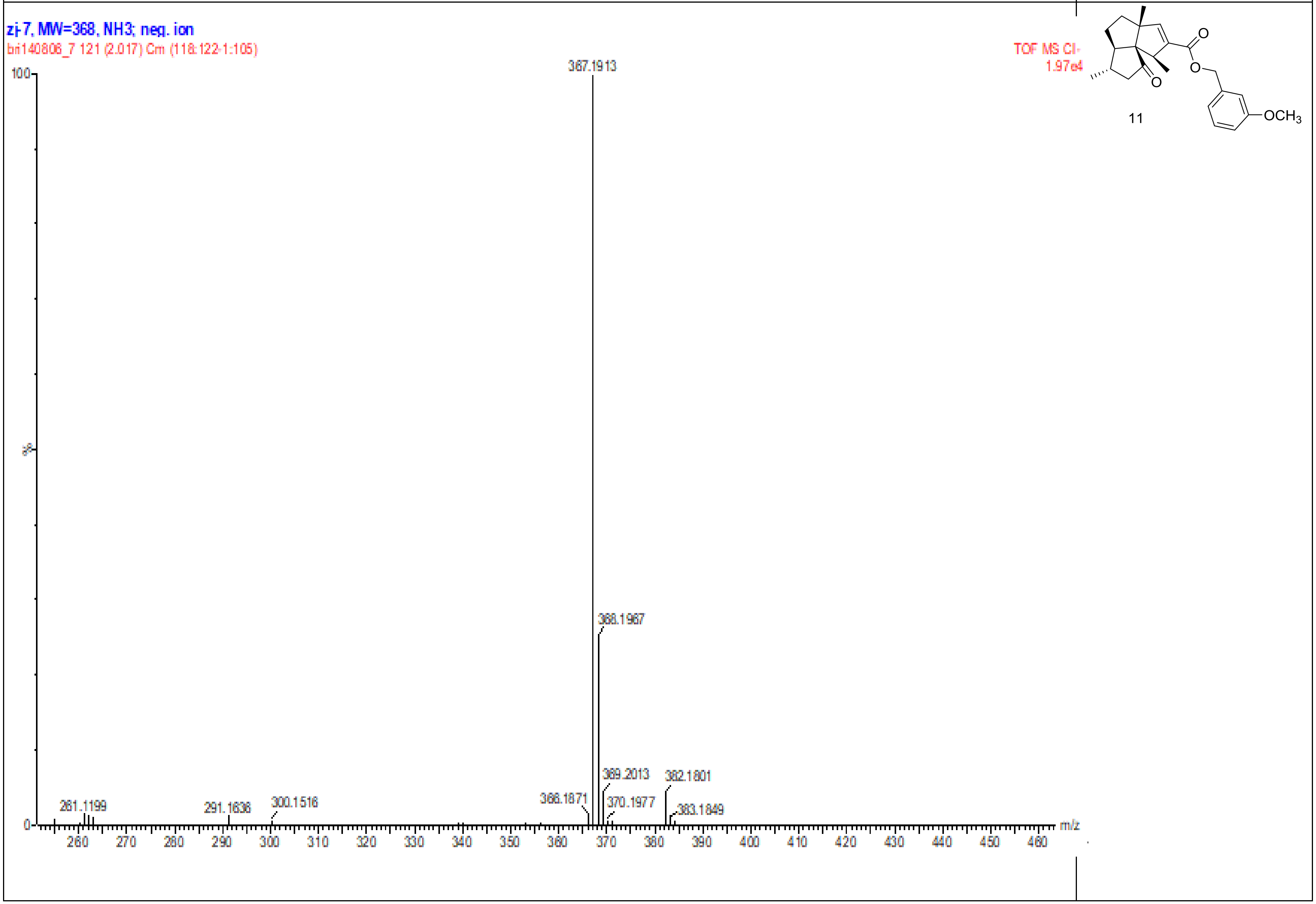

Figure S63. HRMS of compound **13**

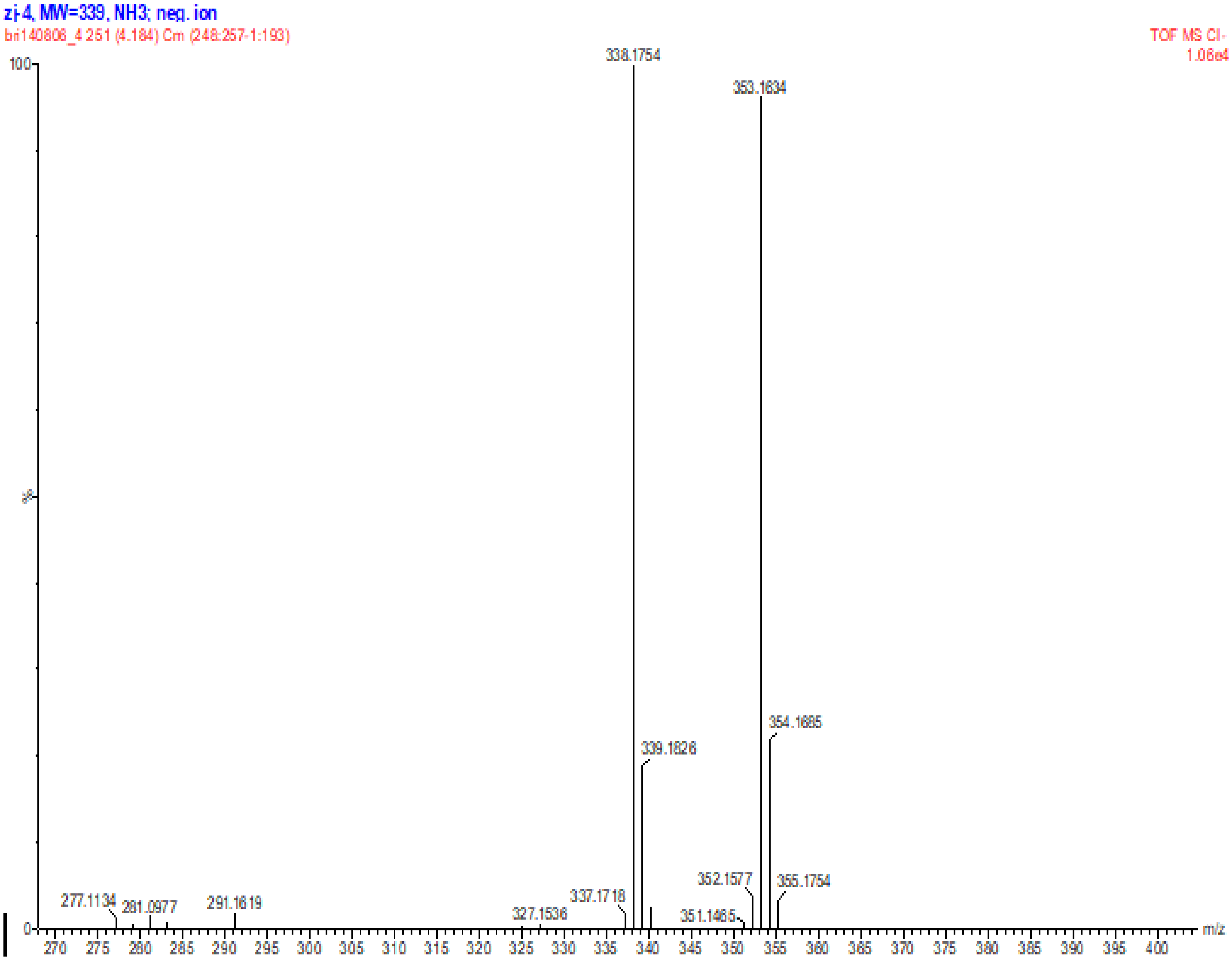

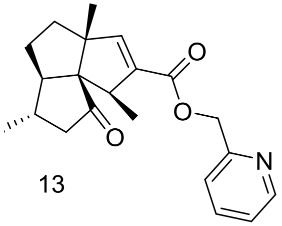

13

Figure S64. HRMS of compound **14**

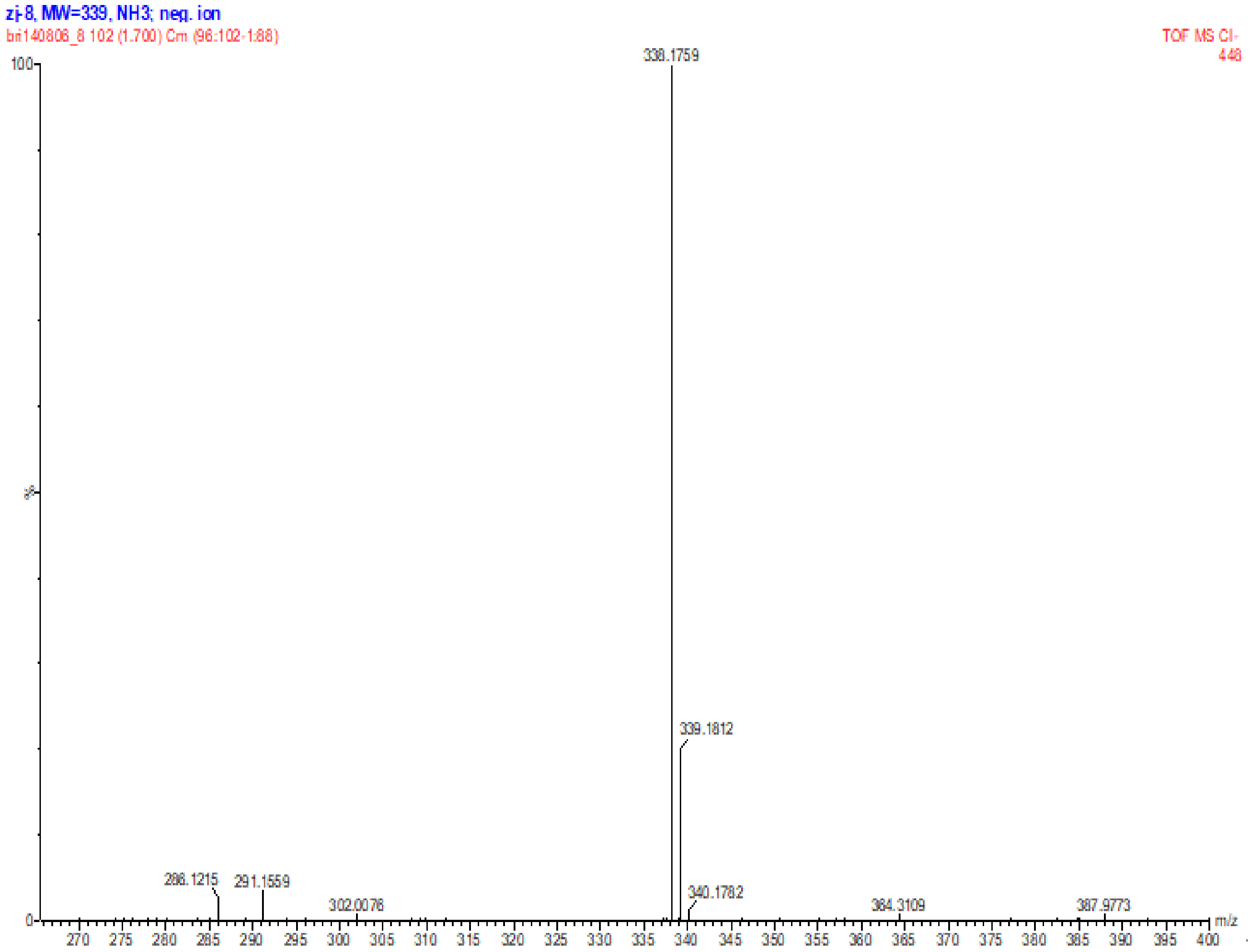

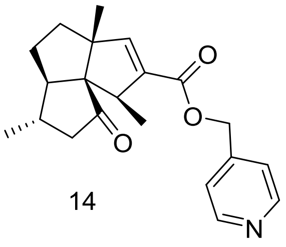

14

Figure S65. HRMS of compound **1**

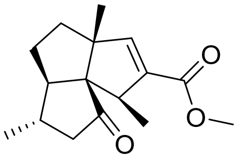

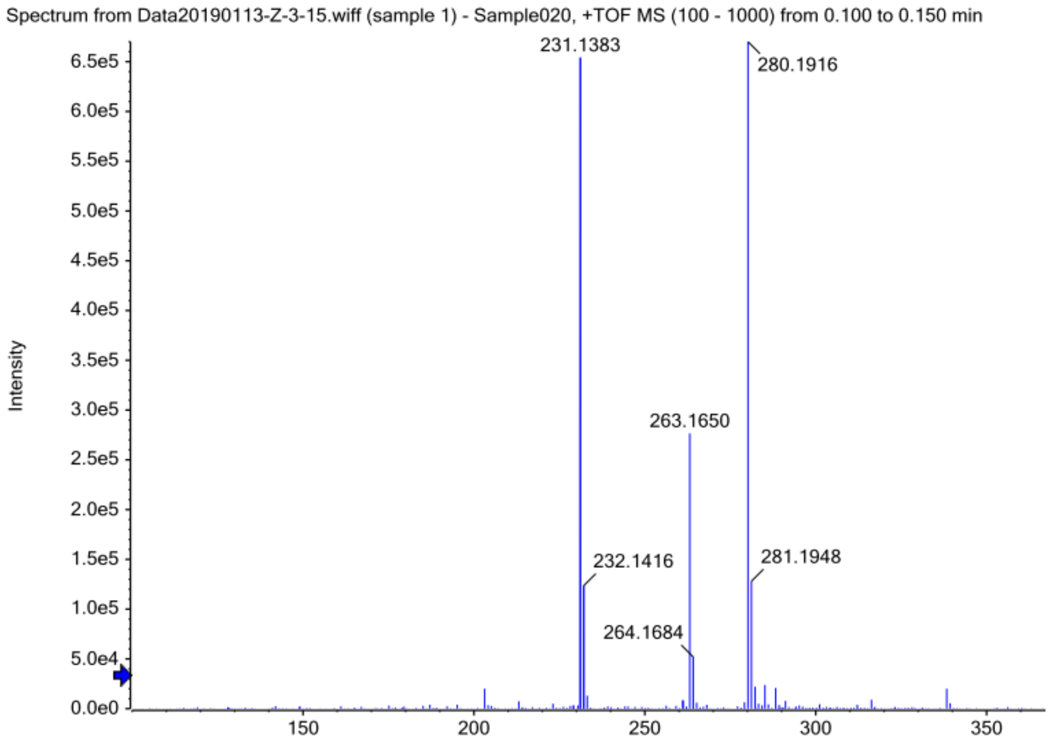

Figure S66. HRMS of compound **2**

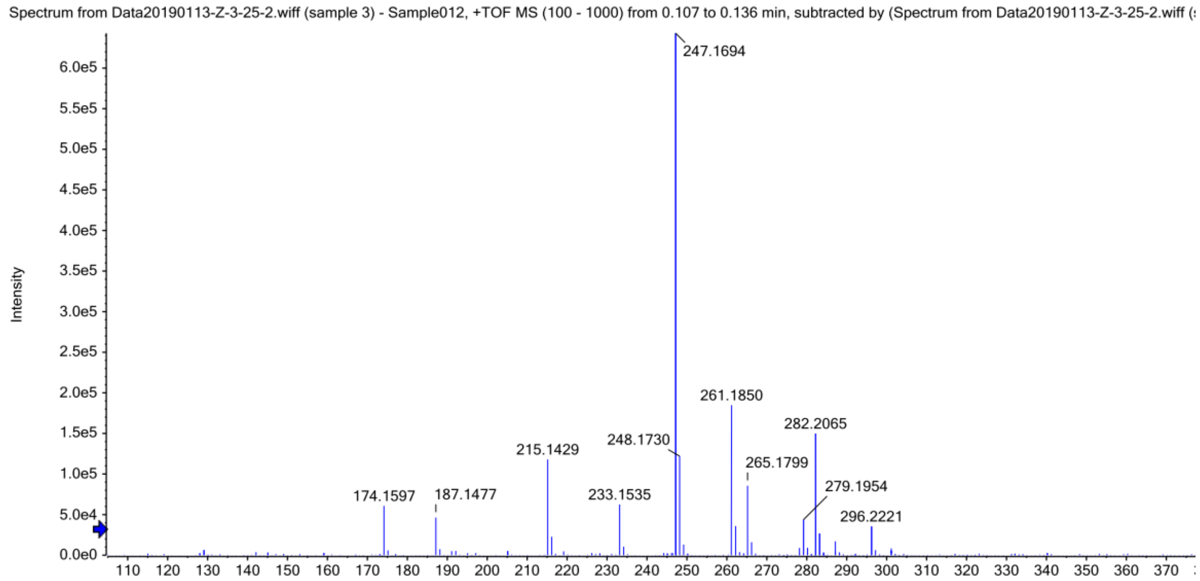

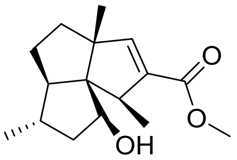

**Figure S67**. HRMS of compound **3**	
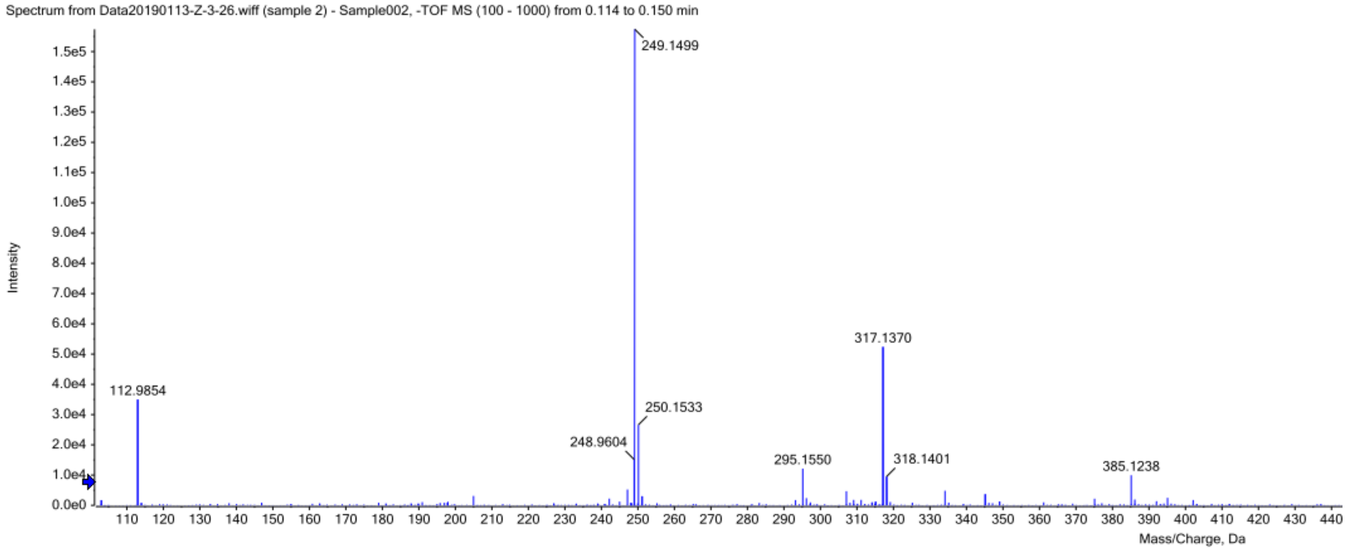	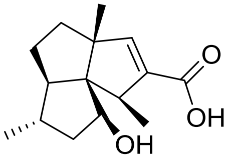

Figure S68. HRMS of compound **6**

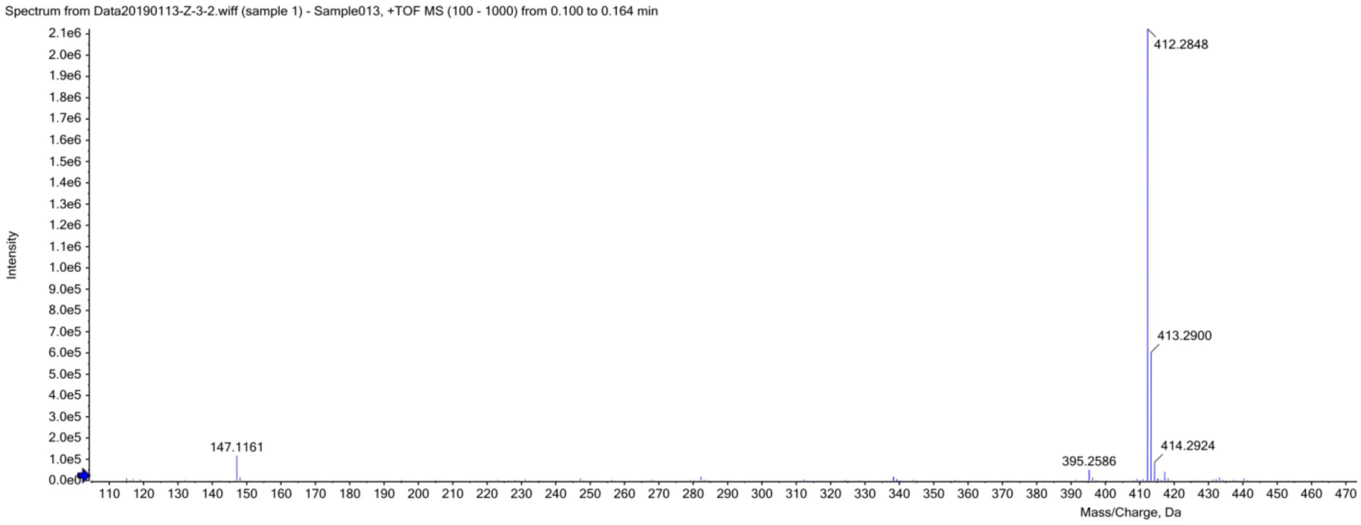

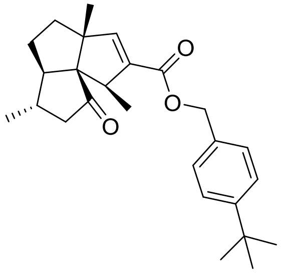

Figure S69. HRMS of compound **7**

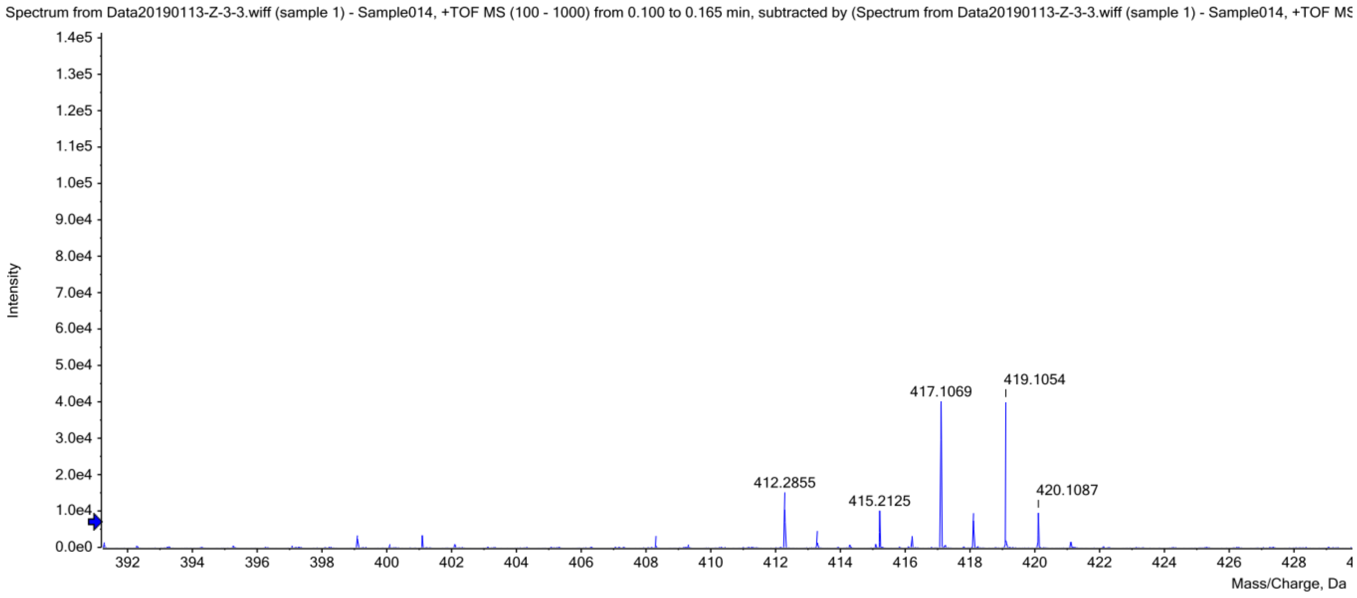

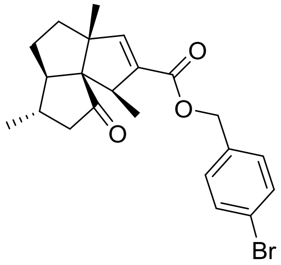

Figure S70. HRMS of compound **9**

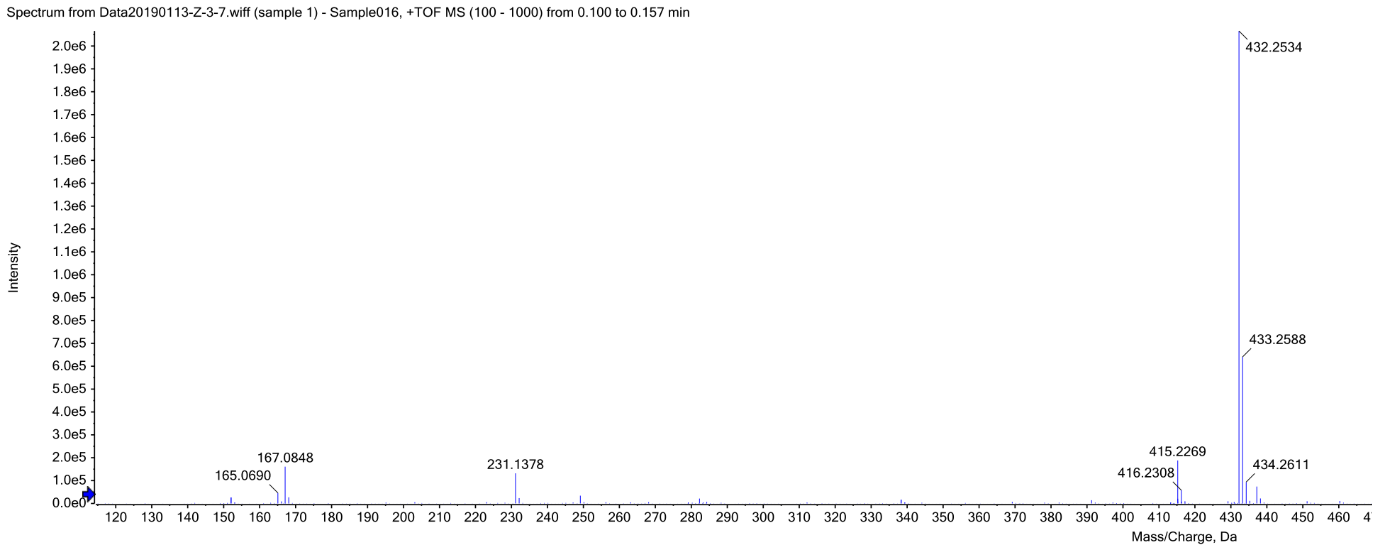

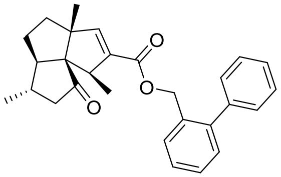

Figure S71. HRMS of compound **12**

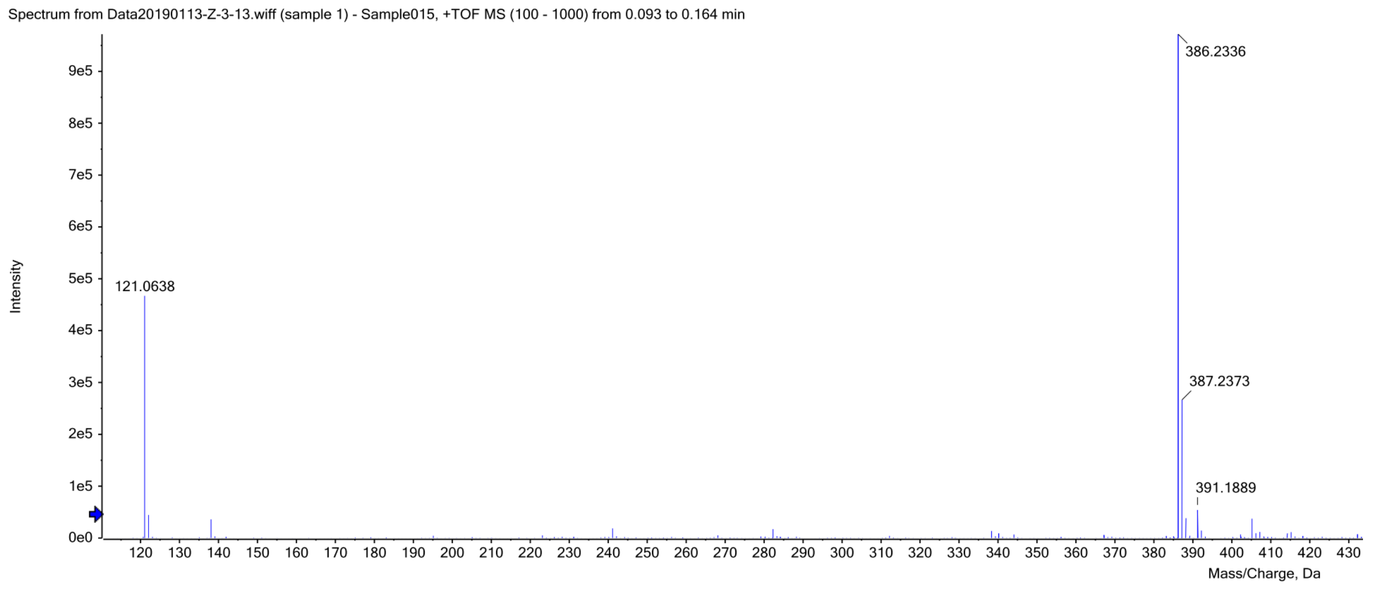

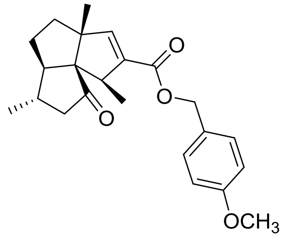

Figure S72. HRMS of compound **15**

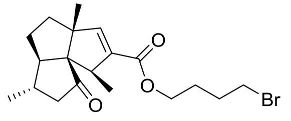

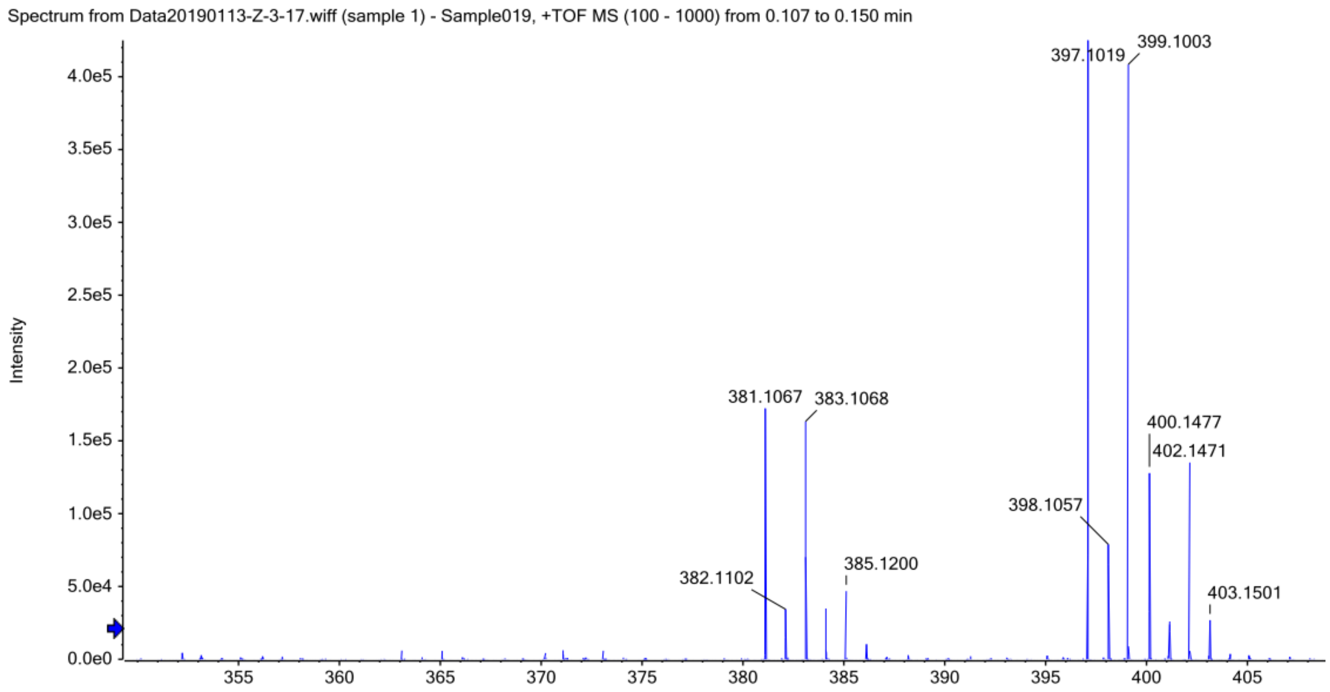

**Figure S73**. HRMS of compound **16**	
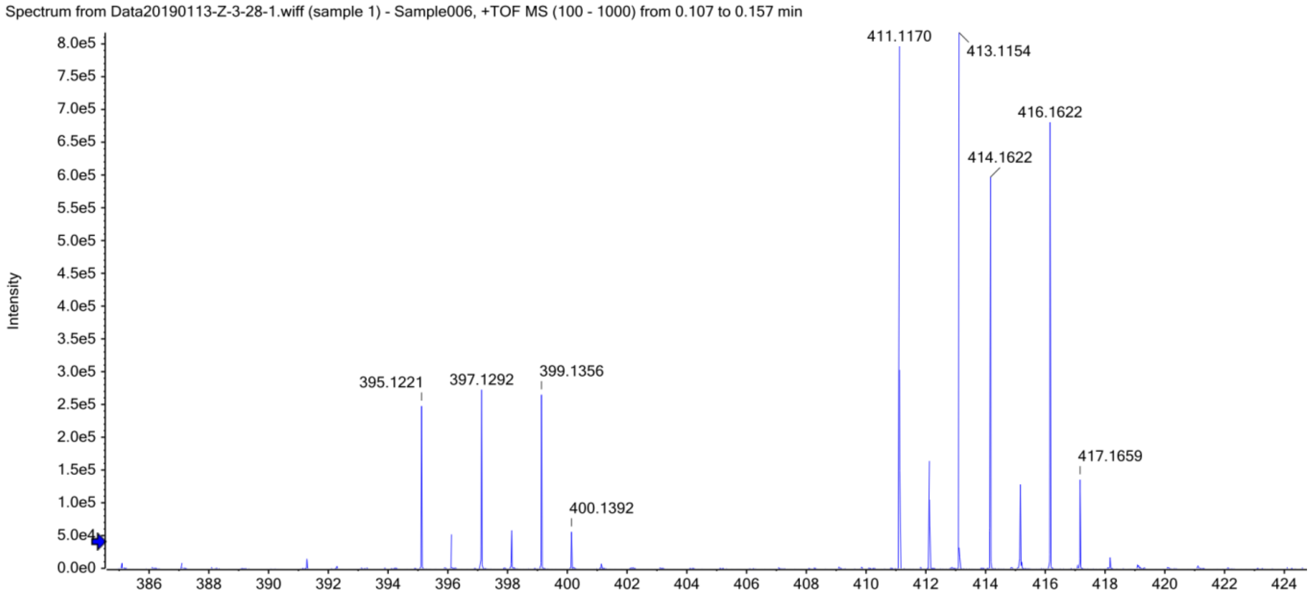	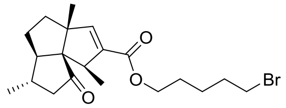

**Figure S74**. HRMS of compound **17**	
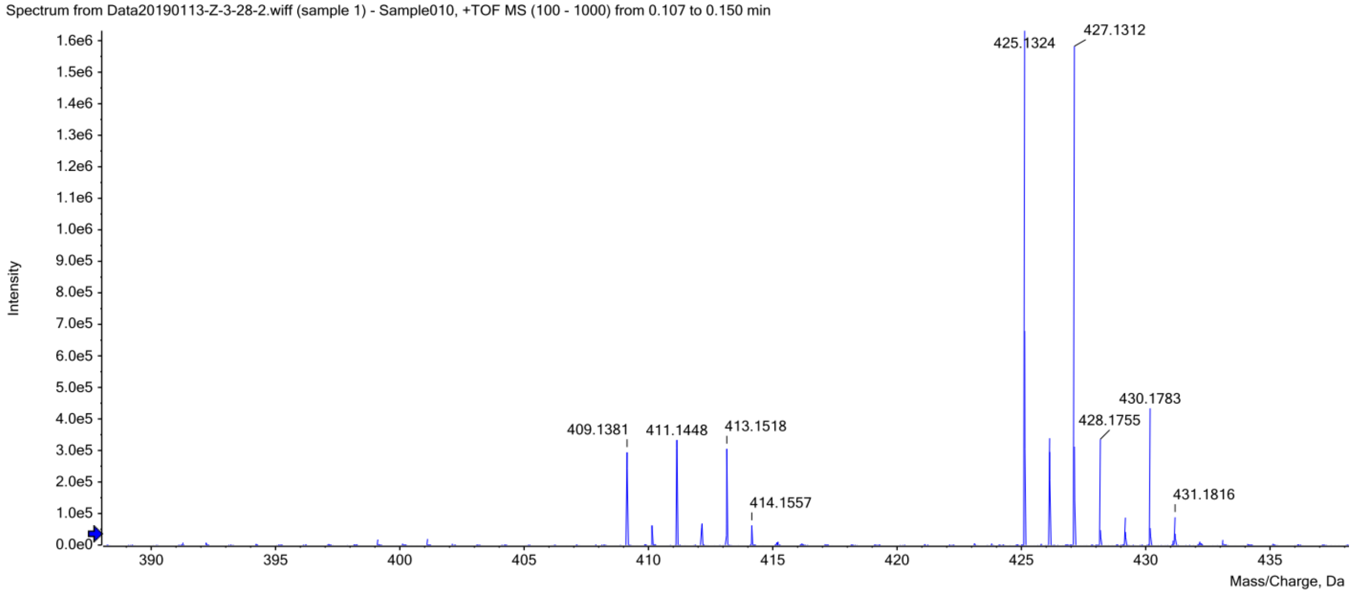	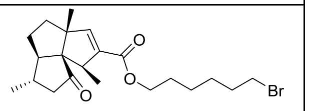

Figure S75. HRMS of compound **18**

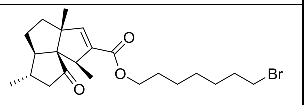

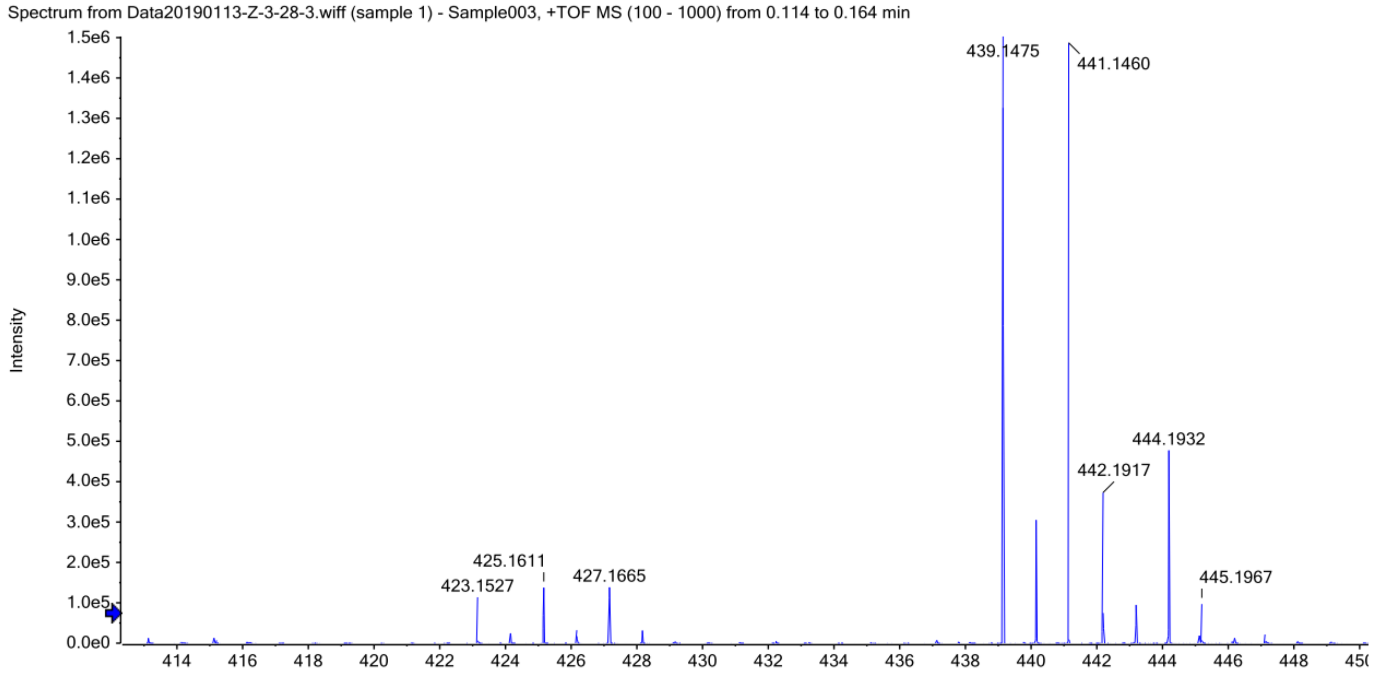

**Figure S76**. HRMS of compound **19**	
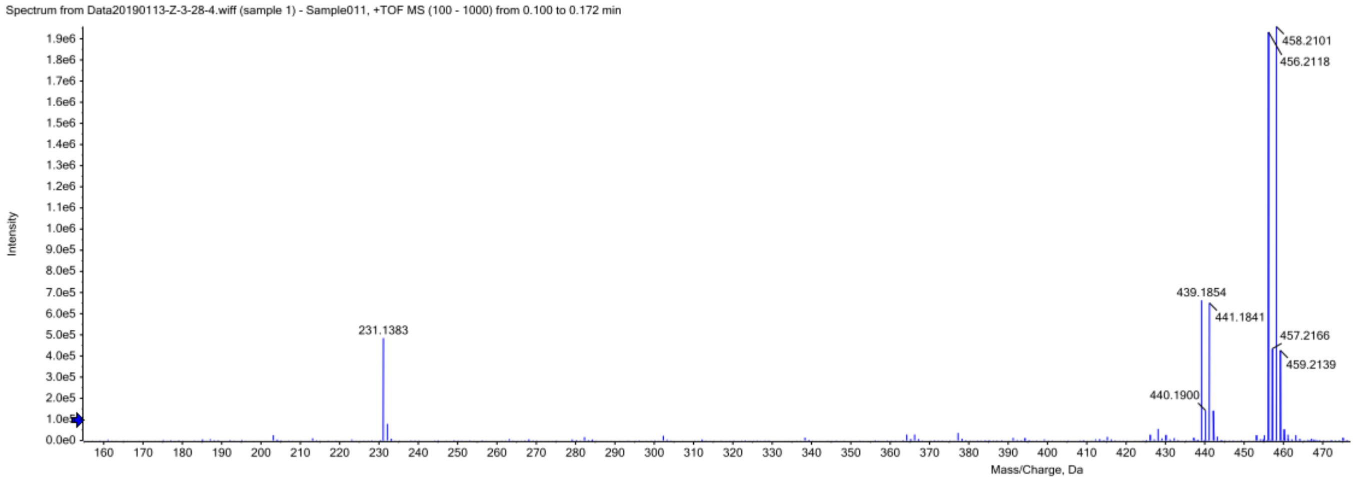	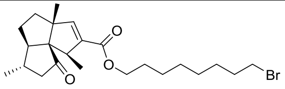

Figure S77. HRMS of compound **20**

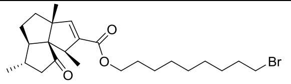

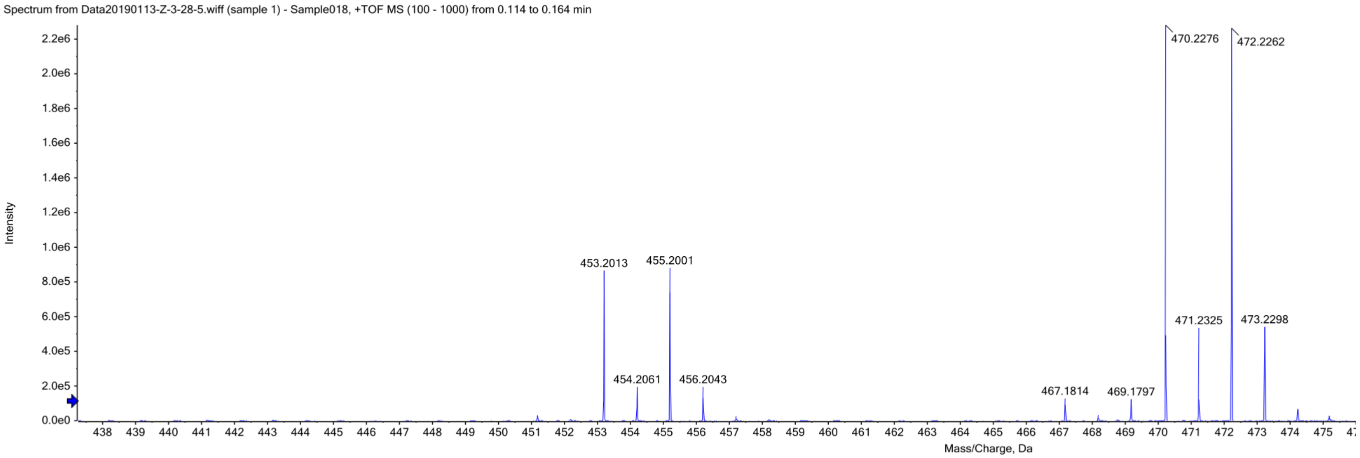

Figure S78. HRMS of compound **21**

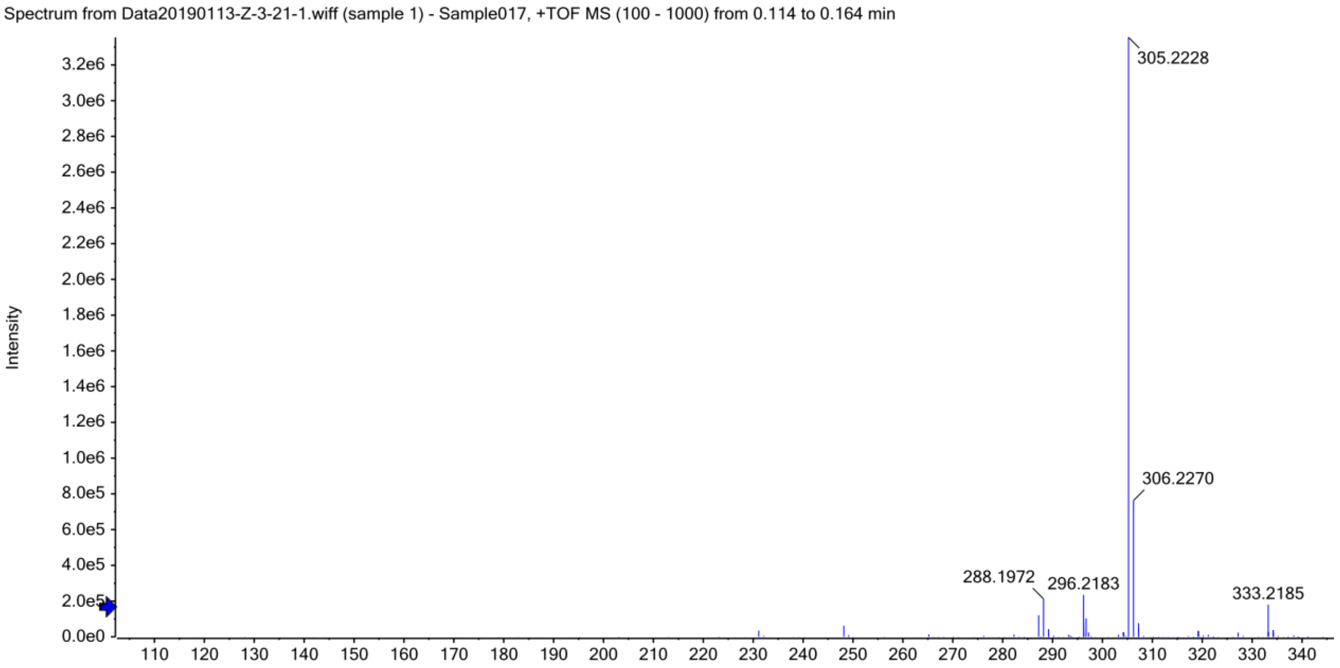

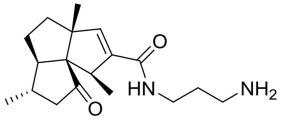

Figure S79. HRMS of compound **22**

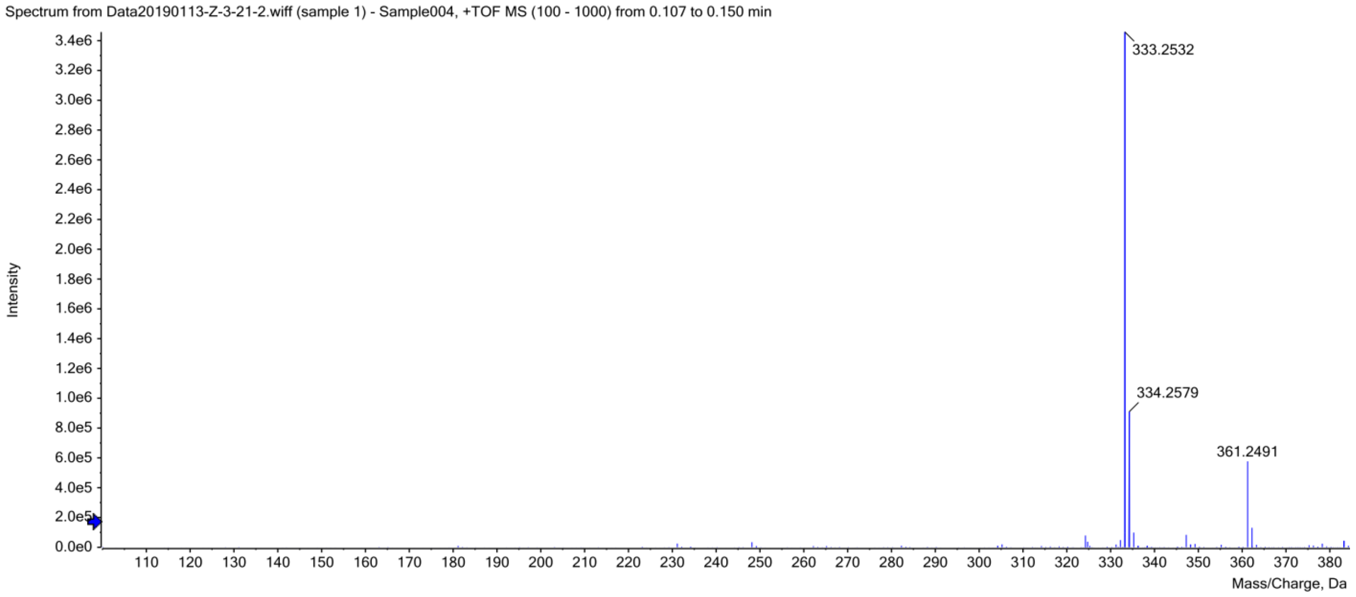

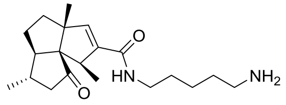

Figure S80. HRMS of compound **23**

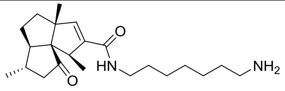

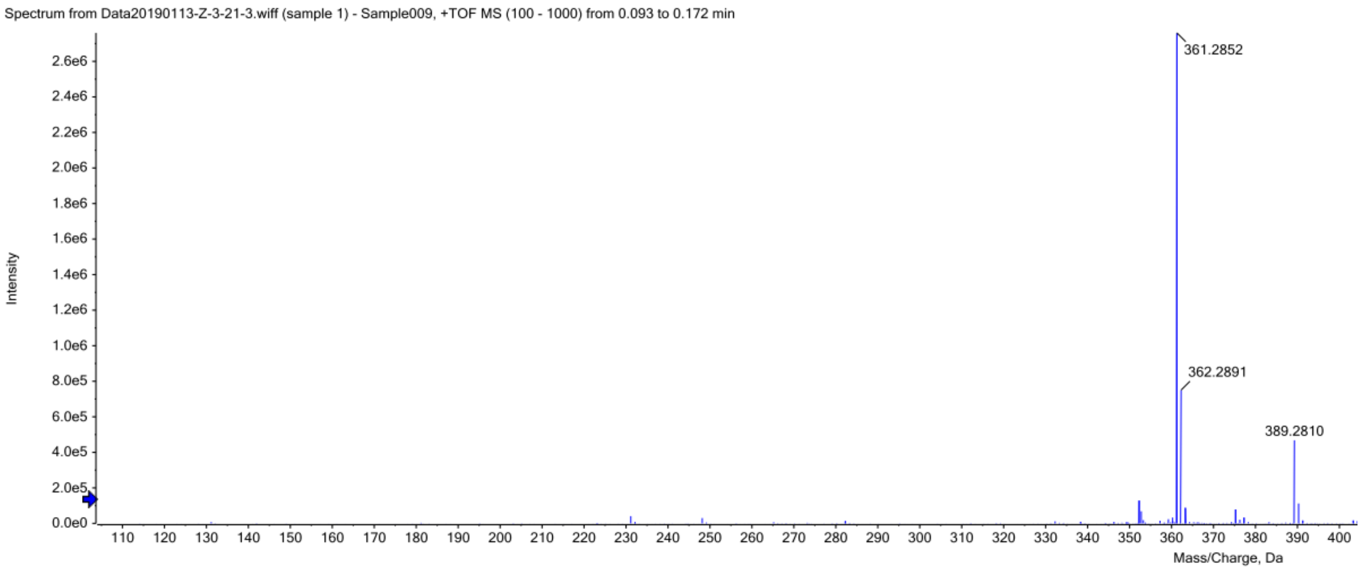

**Figure S81**. HRMS of compound **24**	
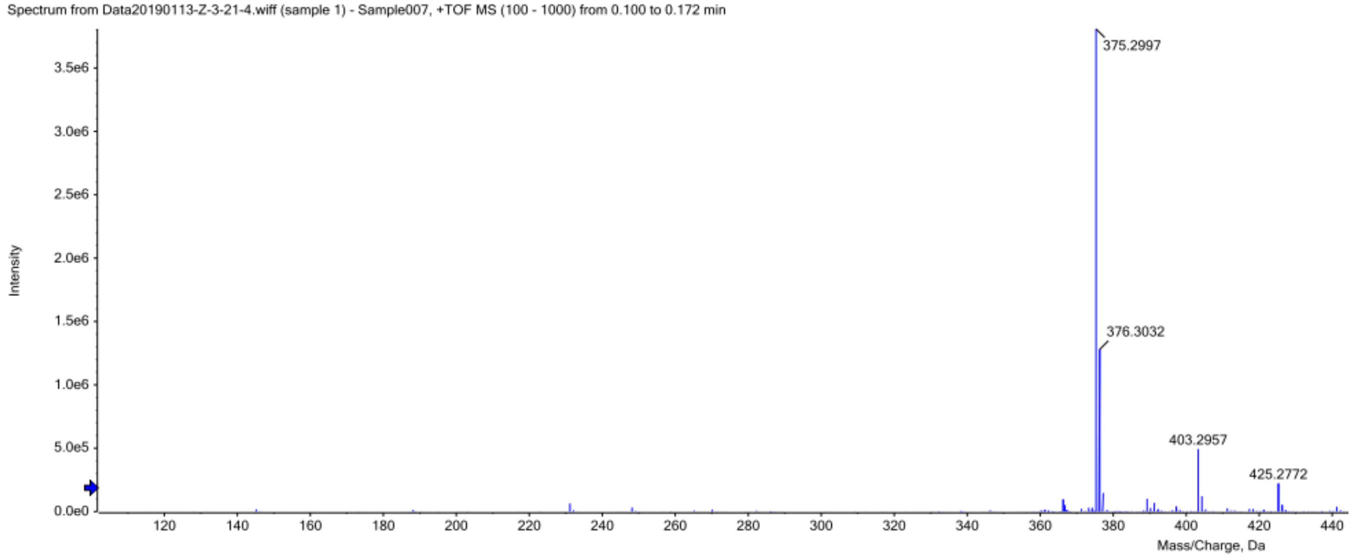	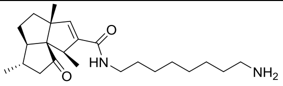

Figure S82. HRMS of compound **25**

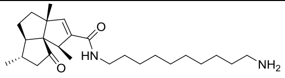

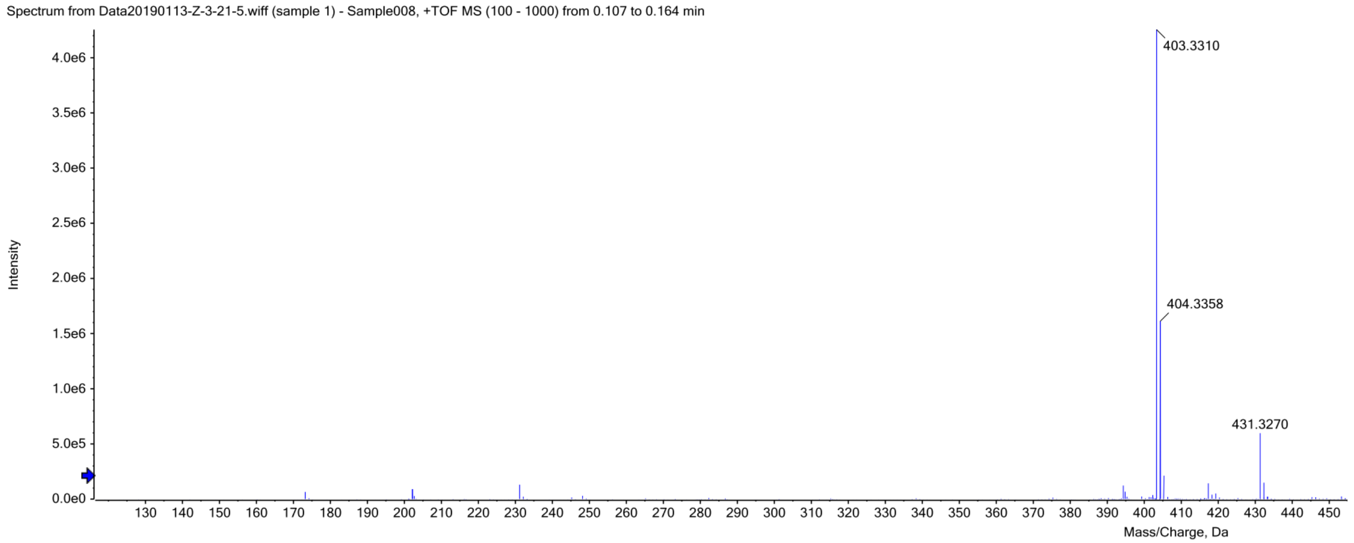

Figure S83. HRMS of compound **26**

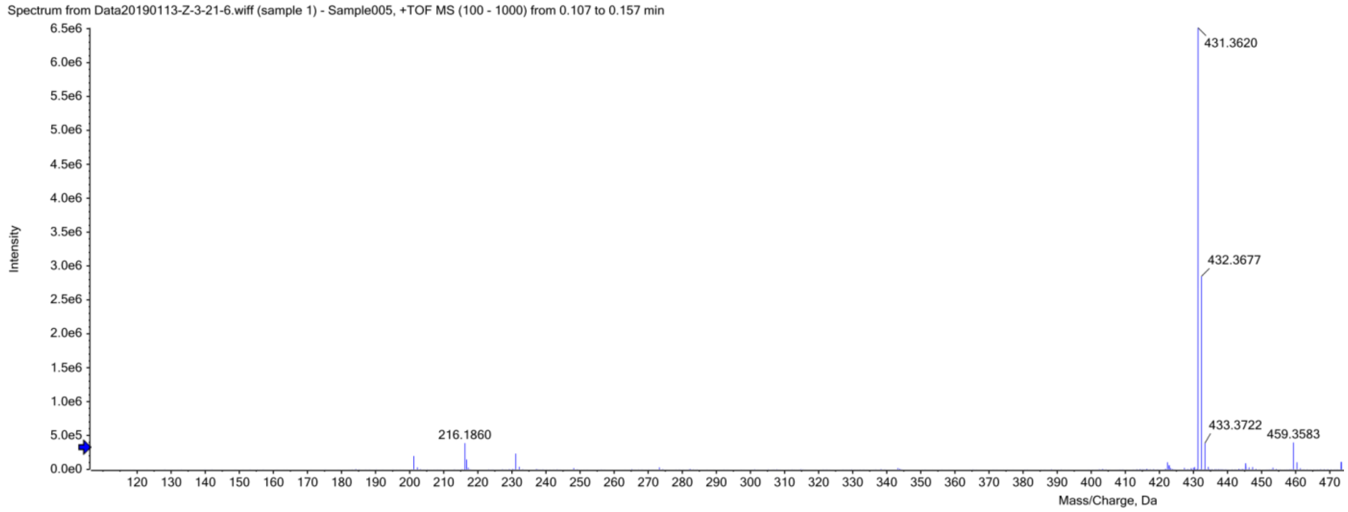

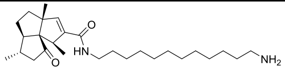

Supplement: Supplementary file 1 [file marinedrugs-17-00101-s001.pdf]
